# Transcriptome Profiling of Peripheral Blood in 22q11.2 Deletion Syndrome Reveals Functional Pathways Related to Psychosis and Autism Spectrum Disorder

**DOI:** 10.1371/journal.pone.0132542

**Published:** 2015-07-22

**Authors:** Maria Jalbrzikowski, Maria T. Lazaro, Fuying Gao, Alden Huang, Carolyn Chow, Daniel H. Geschwind, Giovanni Coppola, Carrie E. Bearden

**Affiliations:** 1 Department of Psychiatry and Biobehavioral Sciences, Semel Institute for Neuroscience and Human Behavior, University of California Los Angeles, Los Angeles, United States of America; 2 Interdepartmental Neuroscience Program, University of California Los Angeles, Los Angeles, United States of America; 3 Department of Neurology, University of California Los Angeles, Los Angeles, United States of America; 4 Department of Psychology, University of California Los Angeles, Los Angeles, United States of America; Maastricht University, NETHERLANDS

## Abstract

**Background:**

22q11.2 Deletion Syndrome (22q11DS) represents one of the greatest known genetic risk factors for the development of psychotic illness, and is also associated with high rates of autistic spectrum disorders (ASD) in childhood. We performed integrated genomic analyses of 22q11DS to identify genes and pathways related to specific phenotypes.

**Methods:**

We used a high-resolution aCGH array to precisely characterize deletion breakpoints. Using peripheral blood, we examined differential expression (DE) and networks of co-expressed genes related to phenotypic variation within 22q11DS patients. Whole-genome transcriptional profiling was performed using Illumina Human HT-12 microarrays. Data mining techniques were used to validate our results against independent samples of both peripheral blood and brain tissue from idiopathic psychosis and ASD cases.

**Results:**

Eighty-five percent of 22q11DS individuals (N = 39) carried the typical 3 Mb deletion, with significant variability in deletion characteristics in the remainder of the sample (N = 7). DE analysis and weighted gene co-expression network analysis (WGCNA) identified expression changes related to psychotic symptoms in patients, including a module of co-expressed genes which was associated with psychosis in 22q11DS and involved in pathways associated with transcriptional regulation. This module was enriched for brain-expressed genes, was not related to antipsychotic medication use, and significantly overlapped with transcriptional changes in idiopathic schizophrenia. In 22q11DS-ASD, both DE and WGCNA analyses implicated dysregulation of immune response pathways. The ASD-associated module showed significant overlap with genes previously associated with idiopathic ASD.

**Conclusion:**

These findings further support the use of peripheral tissue in the study of major mutational models of diseases affecting the brain, and point towards specific pathways dysregulated in 22q11DS carriers with psychosis and ASD.

## Introduction

Identification of replicable risk genes for developmental neuropsychiatric disorders such as autism and schizophrenia has been hampered by the substantial complexity and heterogeneity of these disorders. Although the majority of cases are of unknown etiology (idiopathic), increasing evidence suggests that rare copy number variants have robust and reproducible impacts on disease risk [1–3]. Investigation of highly penetrant genetic subtypes of these disorders offers a complementary strategy that may elucidate more homogeneous biological pathways [4,5]. The 22q11.2 Microdeletion Syndrome (Velocardiofacial/DiGeorge syndrome; OMIM #192430; 22q11DS) is a particularly compelling model, as it represents one of the most common known recurrent genetic risk factors for the development of these disorders[6]. Multiple studies have found that 23–41% of adolescents and adults with 22q11DS meet criteria for a psychotic disorder [7–13]. Furthermore, 14–50% meet criteria for an autism spectrum disorder (ASD) in childhood [14–17]. Genes within the 22q11.2 locus have been implicated in the etiology of idiopathic psychosis and ASD, providing support for the notion that genes within this region may make a broader contribution to disease susceptibility and/or associated intermediate phenotypes [18–24].

In addition to the elevated rates of psychotic disorder and ASD seen in 22q11DS, there are a diverse range of neuropsychiatric conditions observed in this population, including attention-deficit/hyperactivity disorder (ADHD), anxiety disorders, and mood disorders [7,13]. In fact, at any age, 60% of individuals with 22q11DS meet criteria for a psychiatric disorder of some kind [7,12,13,25] and it is common for an individual with 22q11DS to meet criteria for multiple psychiatric diagnoses concurrently [26]. However, the schizophrenia phenotype in 22q11DS is the most specific psychiatric phenotype identified, as it is the only psychiatric disorder found at a greater rate in 22q11DS than in other neurodevelopmental disorders [6,27], While idiopathic forms of ASD and schizophrenia are both characterized by social cognitive deficits [28] and abnormalities in associated neural circuitry [29], preliminary data in 22q11DS suggests that schizophrenia and ASD are two distinct phenotypes in 22q11DS, as ASD symptoms in childhood did not predict psychosis in adulthood [30]. Thus, a logical next step would be to interrogate these two phenotypes further in 22q11DS, to explore whether gene expression profiles differ amongst these two conditions and possibly identify genes uniquely associated with each disorder.

All 22q11DS patients share a 1.5 Mb deletion at the 22q11.2 locus, encompassing approximately 28 genes; in 85% of the carriers the deletion spans 3 Mb, including 90 known genes [31]. Several CNS-relevant genes are encoded in the 1.5 Mb critical region, including genes involved in neuronal migration, myelination and brain development [32,33]. This region is flanked by proximal and distal low-copy-number repeats (LCRs) or segmental duplications, making this region vulnerable to errors in chromosomal recombination [34,35]. Two previous studies using high-resolution comparative genomic hybridization (aCGH) arrays showed significant breakpoint variability within these LCRs in 22q11DS individuals, suggesting that variability of deletion breakpoints may contribute to the variable clinical presentation observed in 22q11DS [36,37]; however, the pathogenetic mechanisms underlying the development of neuropsychiatric disorders in 22q11DS have yet to be elucidated.

The sole prior publication to date to examine blood-based gene expression profiles in 22q11DS confirmed significantly reduced expression of genes within the 22q11.2 locus in 22q11DS patients relative to controls, and found that a number of functional pathways, including some previously associated with idiopathic schizophrenia, were involved [38]. However, due its small sample size (N = 16) the study was unable to evaluate differential expression related to phenotypic variability in 22q11DS.

Additionally, multiple testing presents a challenge for expression microarray studies, which test thousands of transcripts simultaneously. Gene co-expression network analysis offers an alternative approach, allowing the identification of groups of functionally related genes or ‘modules’, whose expression patterns are highly correlated. These modules can then be compared between cases and controls, and analyzed in relation to both categorical and quantitative phenotypes [39,40].

To better characterize how the 22q11DS genotype gives rise to such varied phenotypes, we sought to answer the following questions: 1) Is there variation in deletion breakpoints in 22q11DS?; 2) are there differences in gene expression between 22q11DS patients and controls?; 3) are there networks of co-expressed genes associated with psychotic illness or ASD diagnosis in 22q11DS?;, and 4) can we validate our results against published datasets available from other samples of both peripheral blood and brain tissue from idiopathic psychosis and ASD cases?

## Methods and Materials

### Subjects

Forty-six patients with a molecularly confirmed diagnosis of a 22q11.2 deletion and 66 unaffected healthy controls were recruited from an ongoing longitudinal study at the University of California, Los Angeles. 22q11DS participants were recruited from posts to 22q11DS/Velocardiofacial online foundations and flyers through contacts with local craniofacial or genetics clinics. Controls were recruited from flyers posted at local schools and community centers. Demographic information is reported in Table 1. Psychotropic medication usage for 22q11DS participants is reported in S1 Table.

**Table 1:** Demographic information for 22q11DS participants and controls.

	22qDS (N = 46)	Unrelated Controls (N = 24)	Family Member Controls (N = 42)	*p*-value
Age (+/-SD)	17.3 (11.9)	15.4 (5.2)	41.7 (12.9)	*p* < .001
Gender (N, % female)	23 (50%)	8 (33%)	14 (33%)	*p* = .21
Psychotic Disorder	6 (13%)	NA	NA	
Autism Spectrum Disorder	16 (40%)^*a*^ ^,^ ^*b*^	NA	NA	
WASI IQ (mean, +/-SD)	76.6 (13.4)^*c*^	112.3 (20.9)^d^	NA	*p* < .001
SRS T Score (mean, +/-SD)	69.7 (16.0)	52.7 (14.8)	NA	*p* < .001

^*a*^assessed in 40 participants

^*b*^3 22q11DS participants had comorbid diagnoses of ASD and psychotic disorder

^*c*^assessed in 43 participants

^*d*^assessed in 23 participants.

This study was approved by the UCLA Institutional Review Board and performed in accordance with the Declaration of Helsinki. All subjects or their legal guardians provided written informed consent, after study procedures were fully explained.

### Whole-genome Microarrays

RNA was extracted from whole blood using the PAXgene extraction kit (Qiagen), then stored at -80C for subsequent analysis. RNA quantity was assessed with Nanodrop (Nanodrop Technologies) and quality with the Agilent Bioanalyzer (Agilent Technologies). Whole-genome transcriptional profiling was performed using Illumina Human HT-12 microarrays. 200 ng of total RNA were amplified, biotinylated and hybridized to Illumina Human V4-HT-12 Beadchips, including approximately 47,000 probes, following the manufacturer’s recommendations. Slides were scanned using Illumina BeadStation, and the signal was extracted by using Illumina BeadStudio software.

### Comparative Genomic Hybridization Arrays

To characterize the boundaries of the 22q11.2 deletion, we used a custom NimbleGen 12*135 HX12 array (see S1 Text).

### Outcome Measurements

Master’s or PhD-level clinicians conducted all assessments. Prior to conducting diagnostic interviews, clinicians had to achieve good to excellent reliability with a set of gold standard ratings (kappa coefficients of .90 or greater for categorical diagnoses and intra-class correlations coefficients (ICC’s) ranging from 0.85 to 1.00 for quantitative symptom ratings).

For all participants, DSM-IV psychotic disorder diagnosis was determined through a parental interview using the Computerized Diagnostic Interview Schedule for Children (C-DISC,[41] 6–9 years) and/or the Structured Clinical Interview for DSM-IV Axis I Disorders (SCID) [42,43], with an additional developmental disorders module, administered to the proband and parent (10 years and up). 22q11DS participants were also administered the SIPS/Scale of Prodromal Syndromes (SOPS), a dimensional measure of psychotic symptom severity, including sub-threshold (prodromal) and fully psychotic symptoms [44]. Symptoms on this scale are rated from 0–6, with zero representing an absence of symptoms and six referring to a severe and psychotic level of symptoms. Diagnostic formulations and SIPS symptom ratings for all cases were additionally reviewed in consensus diagnosis meetings led by the study director, a board-certified psychologist (CEB).

Diagnoses of autism and/or ASD were determined using the Autism Diagnostic Observation Schedule (ADOS)[45], administered to the child, and the Autism Diagnostic Interview-Revised (ADI-R)[46], administered to the subject’s parent/primary caretaker. Participants were classified as having ASD, based on the ADI-R, if scores were above threshold for the Reciprocal Social Interaction domain, as well as either Communication Impairment or Repetitive Behaviors and Stereotyped Patterns. Scores from the ADOS and ADI-R were used to determine a consensus diagnosis of ASD, as described in a prior publication [47]. Seven subjects in the 22q11DS group were over the age of 18 and thus were not administered the ADI-R/ ADOS; these subjects and their parents/primary caretakers were administered a SCID interview, with an additional developmental disorders module as described above, in order to determine ASD diagnostic status according to DSM-IV diagnostic criteria. Six adult 22q11DS patients (ages 34–61) did not receive the additional developmental disorders module; thus, they were omitted from the ASD analyses due to the difficulty of making such a diagnosis retrospectively, in the absence of parental information.

### Statistical Analyses

#### Microarray-based gene expression analysis

Raw data were analyzed by using Bioconductor packages in the R statistical environment as previously described. [48] Only samples with an RNA integrity number (RIN) of 7 or greater were included in the analyses [49]. Quality assessment was performed by examining the inter-array Pearson correlation and clustering based on the top variant genes was assessed. We directly removed batch effects using ComBat, which is part of the Bioconductor package in R [46]. Prior to running any analyses, we corrected for age by using a linear model to regress out the participant’s age from each transcript and calculate a residual value.

We then performed the following contrast analyses separately: 1) Controls vs. 22q11DS, 2) 22q11Ds-PSY+ vs 22q11DS-PSY-, and 3) 22q11DS-ASD = vs 22q11DS-ASD-. Analyses of 22q11DS-PSY+ vs. controls and 22q11Ds-PSY- vs. controls are reported in the Supplementary Text. Contrast analysis of differential expression was performed by using the LIMMA package [50]. After linear model fitting, a Bayesian estimate of differential expression was calculated. Similar to previous publications examining differential gene expression [51], transcripts were considered significantly DE at *p* < .005 (uncorrected).

Similar to previous publications which have investigated the statistical significance of the overlap between gene lists[51,52], we then conducted hypergeometric distribution tests [53] using the phyper function in R to determine whether the DE transcripts were significantly enriched for brain-expressed genes. This method tests for significance of over-representation of genes within two comparative samples. Unique gene transcripts examined (28,503) were considered the background list. We identified the overlap between unique names of genes identified as brain-expressed [42] and the DE genes obtained from each of our contrasts and calculated the probability of drawing randomly drawing this many overlapping genes from the background list. For DE genes identified in the 22q11DS vs. control contrast, we also conducted a hypergeometric distribution test to identify whether genes in the TDR were over-represented. Bonferroni correction was used to correct for multiple comparisons (N = 4; *p* = .013).

For these analyses, Gene ontology (GO) annotation was performed using DAVID (http://david.abcc.ncifcrf.gov/) and terms that remained significant after correction for False Discovery Rate (*q* ≤ .05) [54] were considered relevant to the analysis of interest. Pathway analysis was performed using the Functional Analysis Annotation tool in the Ingenuity Pathways Analysis (IPA) software (Ingenuity Systems, www.ingenuity.com). IPA uses literature-based data (scientific publications) to detect over-representation of biologically relevant networks and canonical pathways within the respective set of genes. The algorithm used to determine gene networks and pathways is described in detail in Calvano et al., 2005 [55] and at the following web link: www.ingenuity.com/wp-content/themes/ingenuitytheme/pdf/ipa/IPA-netgen-algorithm-whitepaper.pdf. Once networks are assigned, a right-tailed Fisher’s exact test is used to calculate a p-value that determines the probability that the biological functions/terms assigned to that network was due to chance. A score is computed for each network (-log10 of the *p-*value). A score of 2 (*p*-value = .01) indicates that there is a 1 in 100 chance that the genes found together in the identified network occur due to random chance. Thus, scores greater than 2 have at least 99% confidence of not being due to random chance. For all analyses, we report on the top network identified. Microarray data have been deposited in the NCBI Gene Expression Omnibus database (www.ncbi.nlm.nih.gov/ geo, Accession number: GSE59216).

#### Analysis of Age Effects

In order to identify age-related transcripts, Pearson correlations were used to examine the relationship between gene expression levels with age in both 22q11DS patients and controls.

#### Secondary Gene Expression Analyses

Since a subset of our controls were biological relatives of 22q11DS patients, we conducted a secondary DE analysis comparing 22q11DS patients vs. their unaffected family members in a paired design. To ensure that age differences and relationships between patients and their unaffected relatives were not driving our results, we re-ran the DE analysis including only a subset of age-matched, unrelated controls (N = 39) from our overall control sample. To ensure that possible confounding factors, such as RNA Integrity Number and gender, were not affecting our analyses, we re-ran the DE analysis after covarying for these factors. We then separately calculated Pearson correlations between the fold change scores in the original DE analysis and the fold change scores obtained in these separate analyses.

#### Weighted Gene Co-expression Network Analysis (WGCNA)

We conducted WGCNA, a systems biology approach used to identify networks of co-expressed genes in relation to phenotypic data [56], using R software [39,40]. Correlation coefficients were constructed between expression levels of genes, and a connectivity measure (topological overlap) was calculated for each gene by summing the connection strength with other genes. Genes were then clustered based on their topological overlap, and groups of co-expressed genes (modules) were identified. Each module is assigned a color, and the first principal component (eigengene) of a module is extracted from the module and considered to be representative of the gene expression profiles in a module. The phenotypic trait of interest is then regressed on the eigengene to determine whether there is a significant relationship between the module and the trait. Phenotypic traits examined within 22q11DS participants included: categorical and dimensional indicators of psychosis (i.e. psychosis diagnosis and total SIPS positive symptoms), ASD, IQ, and gender. Age was not included as a phenotypic trait as it was regressed out prior to conducting WGCNA analyses. For modules that showed a statistically significant relationship with a phenotypic trait of interest (*p* < .05, uncorrected), GO analyses and IPA were conducted. We chose to examine modules with a significant association of *p* < .05 since this was the first investigation linking gene expression modules to clinical phenotypes in 22q11DS. For a more detailed explanation of the WGCNA analysis, see S1 Text. R code used to conduct the analysis can be found in the Supporting Information.

Because 3 of our 22q11DS participants had co-morbid diagnoses of ASD and psychosis, we conducted a post-hoc WGCNA module preservation analysis, examining whether our gene expression modules were preserved in the remaining dataset. We re-ran the WGCNA analysis, using the module assignment of the original dataset. To determine whether the original modules remained preserved when these samples were omitted, we calculated a module preservation Z statistic with modulePreservation function of the WGCNA R Library [40,57]. A Z-statistic greater than 5 reflects significant module preservation in the second dataset.

#### Validation with published datasets

To validate our gene expression and WGCNA results, we conducted hypergeometric probability tests (phyper function in R) between our results and publicly available datasets related to idiopathic psychosis [58] and ASD [59]. Using gene lists obtained from the two DE analyses (22q11DS patients with psychosis (22q11DS-PSY+) versus those without a psychotic disorder diagnosis (22q11DS-PSY-), 22q11DS patients with (22q11DS-ASD+) versus those without an ASD diagnosis (22q11DS-ASD-)), we ran separate hypergeometric tests to determine whether there was enrichment of genes associated with idiopathic psychosis and ASD in each analysis, respectively. Additionally, using gene lists obtained from the psychosis and ASD WGCNA modules, we ran separate hypergeometric tests to determine whether these modules showed enrichment of genes associated with idiopathic psychosis and ASD, respectively. We also ran hypergeometric tests between the DE genes and genes identified as brain-expressed [42], as well as between the psychosis and ASD gene module lists and brain-expressed genes[42], to test for enrichment of brain-expressed genes. Bonferroni correction was used to correct for multiple comparisons (N = 9; *p* = .006).

## Results

### CGH analysis

**Fig 1:**
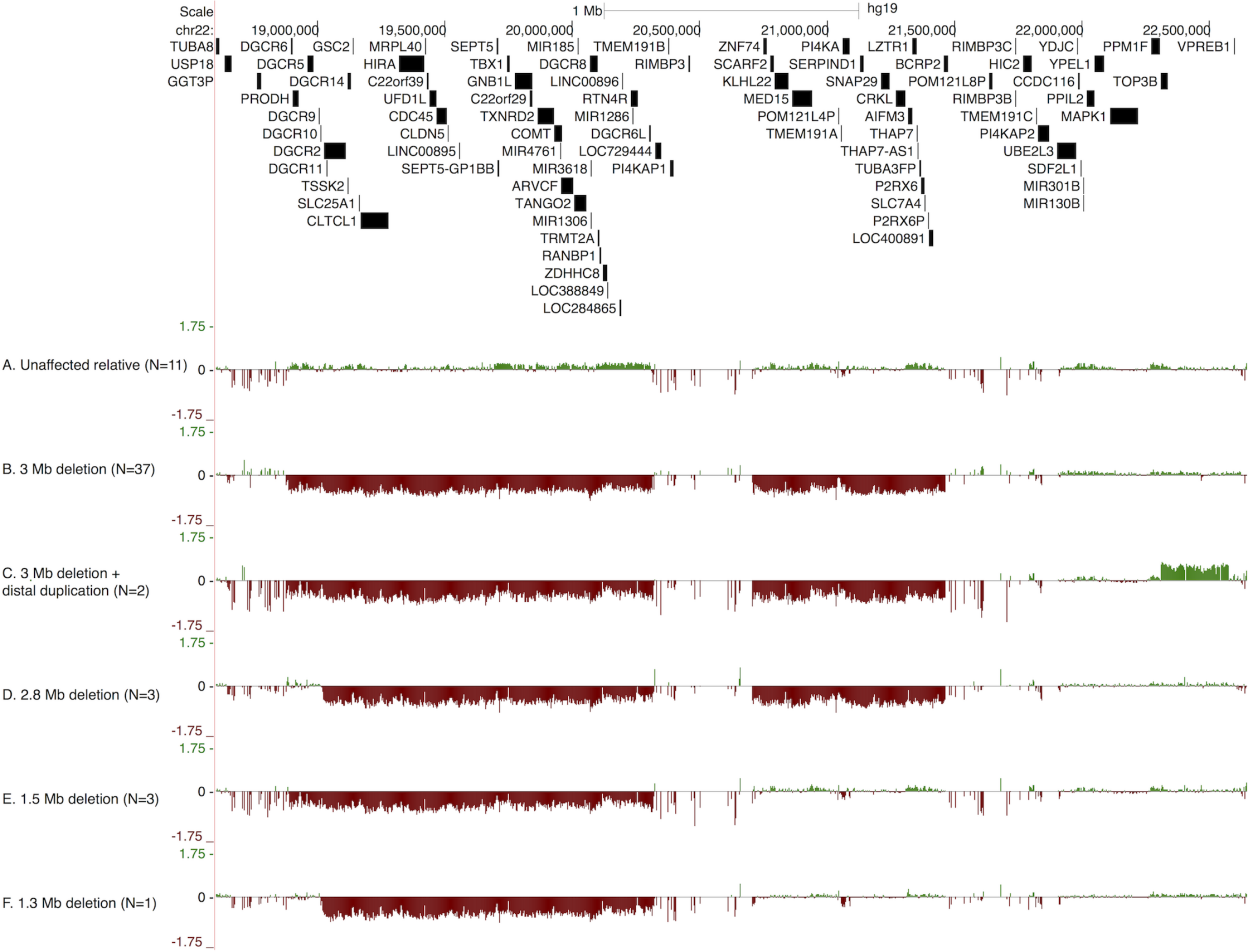
Representative examples of array comparative genomic hybridization (aCGH) using the NimbleGen 12*135 HX12 array, from: A) first-degree relatives of 22q11DS patients (N = 11), B) 22q11DS participant with typical 3 Mb deletion (N = 37); C) 22q11DS participant with 3 Mb deletion and distal duplication, which includes the TOP3B gene (N = 2); D) 22q11DS participant with 2.8 MB deletion (N = 3); E) 22q11DS participant with 1.5 MB deletion (N = 3); and F) 22q11DS participant with 1.3 MB Deletion (N = 1).

Inspection of the intensity plots yielded four distinct subgroups of deletion events in our cohort (Fig 1). Fig 1A shows a characteristic aCGH intensity plot for a first-degree relative. The most frequent aberration observed was the 3MB typical deletion region spanning chr21:18.8–21.5 (N = 37; 80%, Fig 1B). Two individuals (4%) with the 3 MB deletion carried an additional copy-number gain in the region distal to the 3 MB typically deleted region (TDR), which contains the *TOP3B* gene (Fig 1C). Three individuals (7%) carried a 2.8 MB deletion (Fig 1D), three had a 1.5 MB deletion (Fig 1E), and one had a 1.3 MB deletion (Fig 1F). These deletion types have been described previously in the literature [36,60]; however, to our knowledge the copy number gain containing *TOP3B* has not been previously reported. In four individuals (3 with the 2.8 MB deletion and one with the 1.3 MB deletion) the proximal breakpoint was located ~100kb distally from the start of the TDR. Two annotated coding genes, *DGCR6* and *PRODH*, are present in a normal diploid state among these affected individuals.

### Gene Expression Analyses

#### 22q11DS vs. Controls

**Fig 2:**
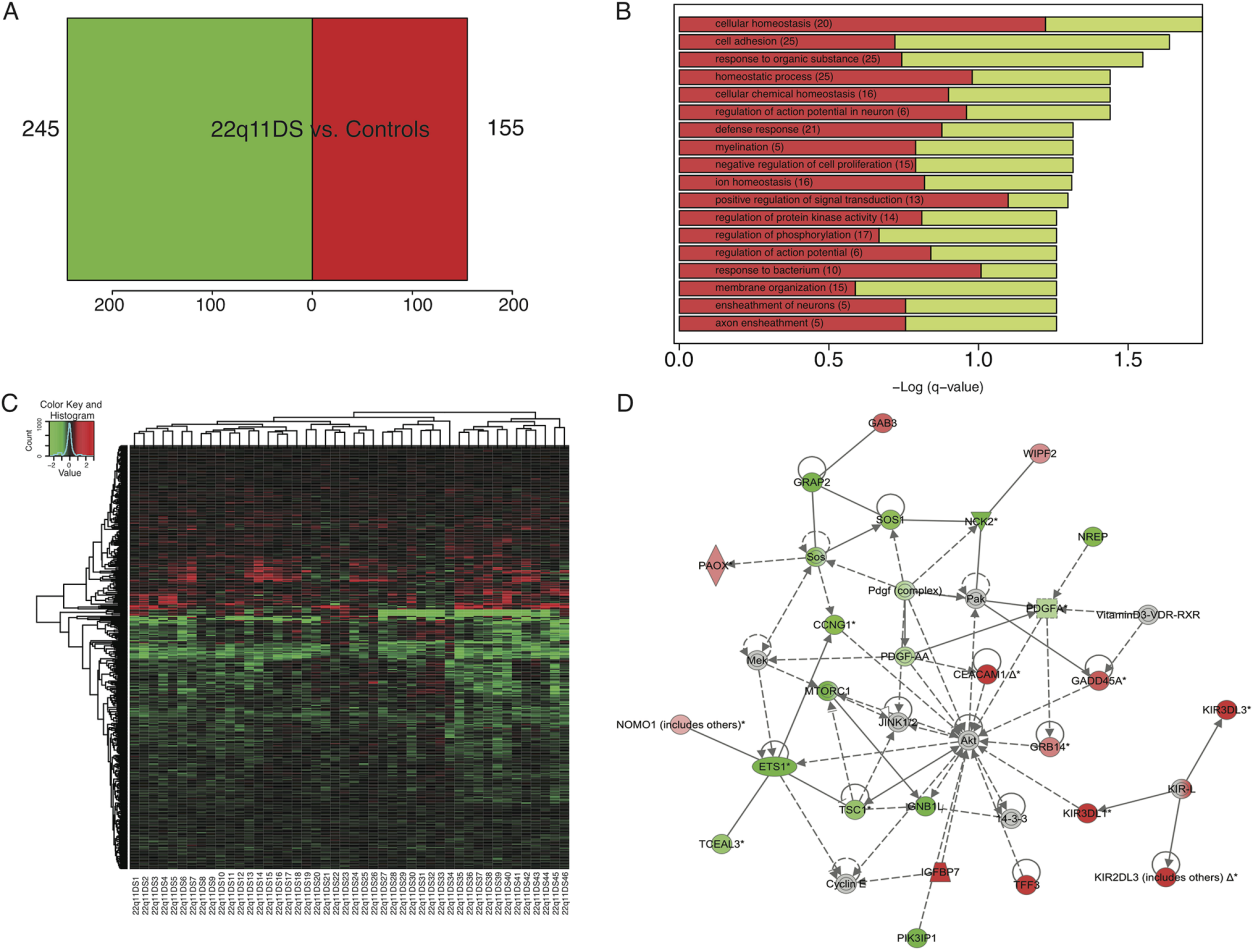
Differential expression (DE) analysis comparing controls to 22q11DS patients. A) Number of DE genes at *p*<0.005. Four hundred probes (245 down-regulated, 155 up-regulated) were DE in 22q11DS vs. controls. B) Gene ontology analysis (*q≤*.05) of DE genes in 22q11DS vs. controls. Top categories included ensheathment of neurons, and regulation of cellular proliferation, signal transduction, and cell communication. Bars represent the–Log of the over-representation *q-*value. Red and green represent the proportion of up and down-regulated genes, respectively. C) Heat map depicting fold changes in peripheral blood samples for each 22q11DS participant. Genes are in rows and samples are in columns. Shades of red indicate up-regulation compared to the control condition, shades of green indicate down-regulation. D) Ingenuity pathway analysis results of a top network associated with significantly DE genes (p < .005) in 22q11DS vs. controls. This network is associated with cellular development, and cellular growth and proliferation. According to IPA, 10 different molecules in this network are associated with axon guidance signaling, including genes *MTORC1*, *GNB1L* and *PDGFA*, which were all down-regulated in 22q11DS patients.

Four hundred probes were DE in 22q11DS vs. controls (*p* < .005, Fig 2A, S2 Table). We observed a significant over-representation of down-regulated probes within the 22q11.2 locus, accounting for 13% of down-regulated genes in 22q11DS (*p* < .0006); however, there was significant variability in expression levels of these genes (S1 Fig). Sixty-five percent of DE genes were brain-expressed [42] (*p* < .00005). GO analyses revealed that the top categories (*q≤*. 05) associated with the DE probes in 22q11DS included regulation of neuronal action potentials, myelination, and axon ensheathment of neurons (Fig 2B). IPA revealed a top network associated with cellular development, and cellular growth and proliferation (Fig 2D). The top canonical pathway associated with this network was axon guidance signaling, with the majority of DE genes down-regulated in 22q11DS patients.

When we removed the genes spanning the 22q11.2 region and re-ran these analyses, our results remained very similar. Eighteen of the 19 reported GO terms overlapped with our original analysis, indicting that the significant GO results were not driven by haploinsufficient genes within the 22q11.2 locus. In addition, three more GO terms were also found to be significant in this second analysis: immune response, regulation phosphate metabolic process, and regulation of epithelial to mesenchymal transition. IPA analyses without the genes spanning the 22q11.2 region showed that the identified top network was still associated with cellular development, and cellular growth and proliferation.

We also conducted secondary analyses comparing 22q11DS-PSY+ vs. controls and 22q11DS-PSY- vs. controls, which can be found in Supporting Information.

#### Psychosis

**Fig 3:**
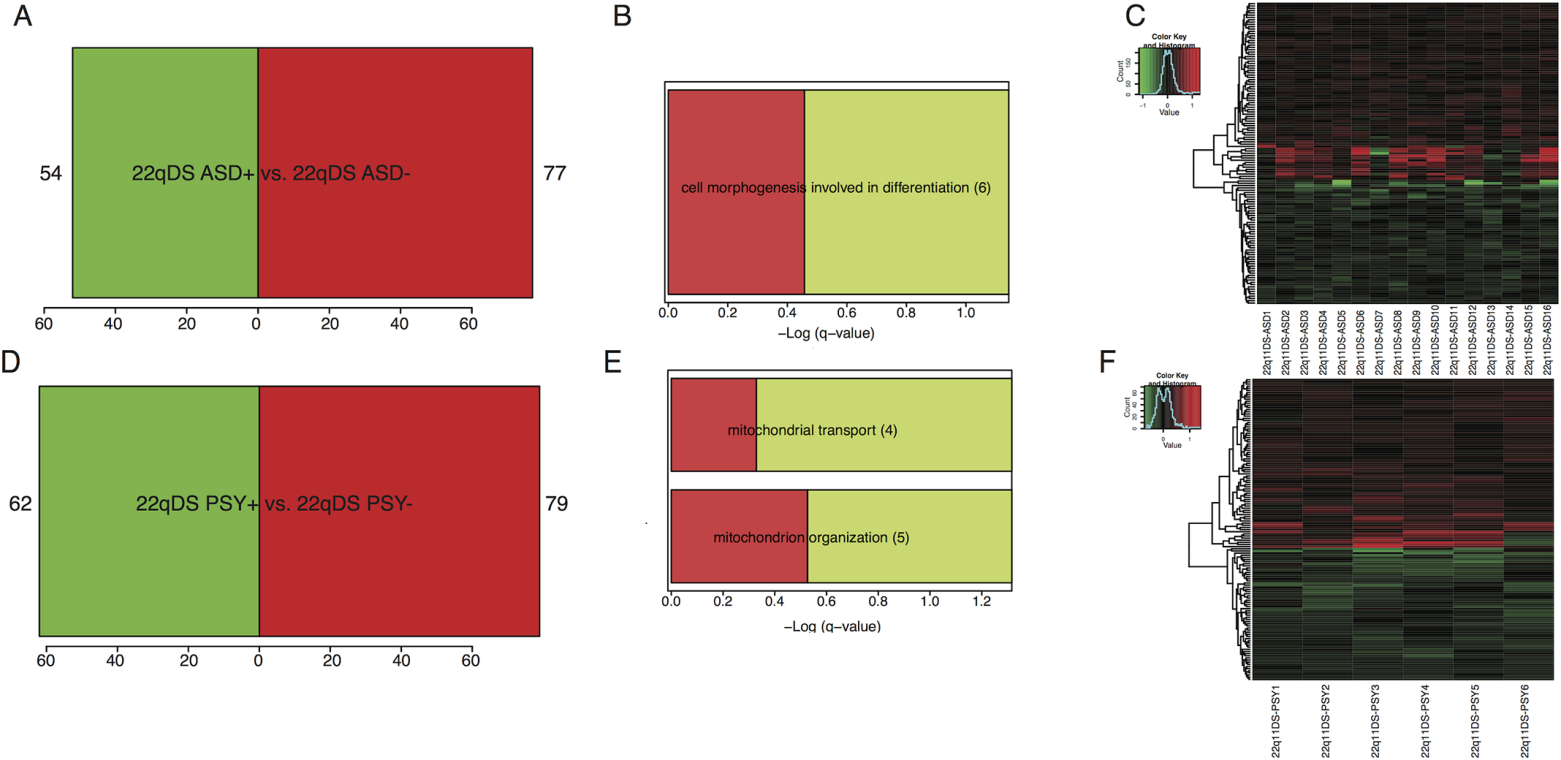
Differential expression (DE) analysis comparing phenotypic expression (i.e., psychosis, autism spectrum disorders) within the 22q11DS sample. A. Number of DE genes at *p*<0.005 for 22q11DS participants with ASD (N = 16) vs. 22q11DS without ASD (N = 34). One hundred thirty one probes (54 down-regulated, 77 up-regulated) were DE in 22q11DS patients with ASD in comparison to 22q11DS patients without ASD. B) Gene ontology analysis (*q≤*.05) of DE genes in 22q11DS with ASD vs. 22q11DS without ASD. The top category was associated with cell morphogenesis involved in differentiation. Bars represent the–Log of the over-representation q-value, as calculated by the DAVID algorithm (http://david.abcc.ncifcrf.gov/). Red and green represent the proportion of up and down-regulated genes, respectively. C) Heatmap depicting fold changes in peripheral blood samples for each 22q11DS participant with ASD. Genes are in rows, and samples are in columns. Shades of red indicate up-regulation compared to the 22q11DS participants without ASD, shades of green indicate down-regulation. D) Number of DE genes at *p*<0.005 for 22q11DS with a psychotic disorder (N = 6) vs. 22q11DS participants without a psychotic disorder (N = 40). One hundred forty one probes (62 down-regulated, 79 up-regulated) were DE in 22q11DS with psychosis in comparison to 22q11DS patients without psychosis. E) Gene ontology analysis (*q≤*.05) of DE genes in 22q11DS with psychosis vs. 22q11DS without psychosis. Top categories were associated with mitochondrial transport and organization. Bars represent the–Log of the over-representation q-value. Red and green represent the proportion of up and down-regulated genes, respectively. F) Heat map depicting fold changes in peripheral blood samples for each 22q11DS participant with psychosis. Genes are in rows, and samples are in columns. Shades of red indicate up-regulation compared to the 22q11DS participants without psychosis, shades of green indicate down-regulation.

Thirteen percent (6/46) of 22q11DS patients had a psychotic disorder diagnosis. 141 probes were significantly DE in 22q11DS-PSY+ relative to 22q11DS-PSY- (Fig 3D and 3F, S3 Table), 51% of which were brain expressed (hypergeometric test, *p* = .44). GO analyses (*q≤*.05) revealed an over-representation of genes associated with mitochondrial organization and transport (Fig 3E). Pathway analyses revealed that the top network of these DE genes was associated with embryonic development and cellular assembly and organization (S2 Fig).

#### Autism Spectrum Disorder

Comparing gene expression profiles of 22q11DS patients with (N = 16) and without (N = 24) an ASD diagnosis, we identified 131 DE probes in 22q11DS-ASD+ relative to 22q11DS-ASD- (Fig 3A & 3C, S4 Table). Forty-five percent of DE genes were considered to be brain expressed [42] (hypergeometric test, *p* = .56). The top GO category (*q≤*.05) associated with the DE genes was cell morphogenesis involved in differentiation (Fig 3B). The top IPA network associated with DE genes in 22q11DS-ASD+ was related to immune response (S3 Fig).

For both DE analyses of psychosis and ASD diagnoses within 22q11DS, none of the DE probes were within the 22q11.2 locus and none of these probes overlapped with the DE genes in the overall (22q11DS vs. control) analysis.

#### Age Relationships

Age effects on gene expression profiles were consistent with those previously documented in other tissue types (S1 Text, S4 Fig).

#### Results from Secondary Gene Expression Analyses

Results remained similar when the DE analyses were re-run on subsets of 22q11DS and control participants (S1 Text). Specifically, we found highly significant fold change correlation coefficients between results from our overall analyses and those for 22q11DS vs. unaffected relatives only, 22q11DS vs. age-matched controls only (*r* = 0.975 and *r* = 0.91, respectively).

### Weighted Gene Co-Expression Analysis

**Fig 4:**
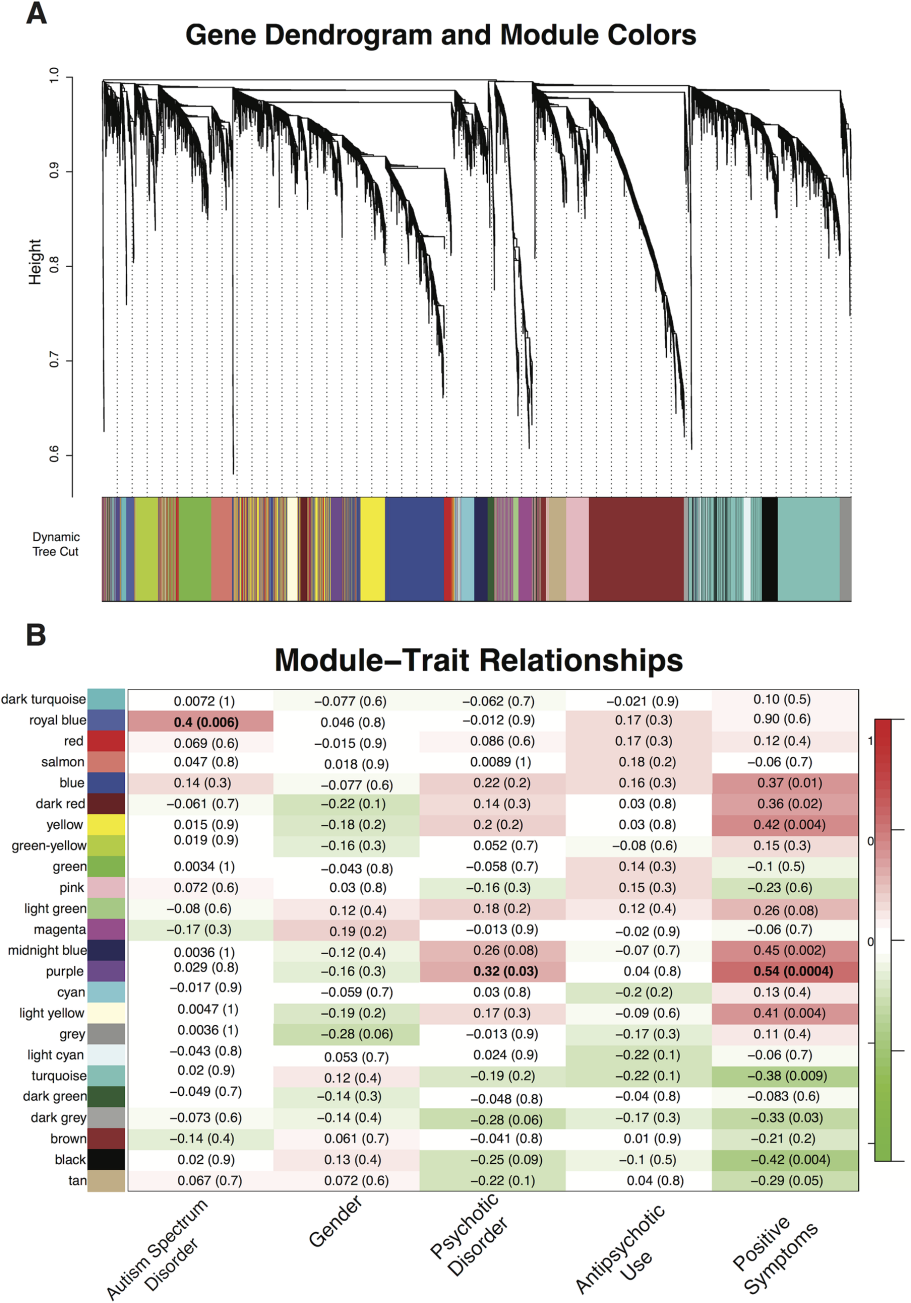
Network construction of co-expressed genes in relation to psychosis and autism spectrum disorders in 22q11DS. A) Dendogram of modules identified through Weighted Gene Co-expression Analysis, created through linkage hierarchical clustering of genes. B) Module-Trait relationships for age, gender, psychotic disorder diagnosis, autism spectrum disorder diagnosis, and positive symptoms, as measured by the SIPs. Numbers shown represent Pearson correlations between the modules and traits. P-values are in parentheses. Numbers on the color bar refer to the strength of the correlation in the table (red = 1, green = -1).

Hierarchical clustering based on topological overlap identified 24 groups of co-expressed genes, or modules (Fig 4A), each comprising between 45 and 1195 genes. Modules identified and the significance values of their relationship with the phenotypic traits of interest are shown in Fig 4B.

#### Psychosis

**Fig 5:**
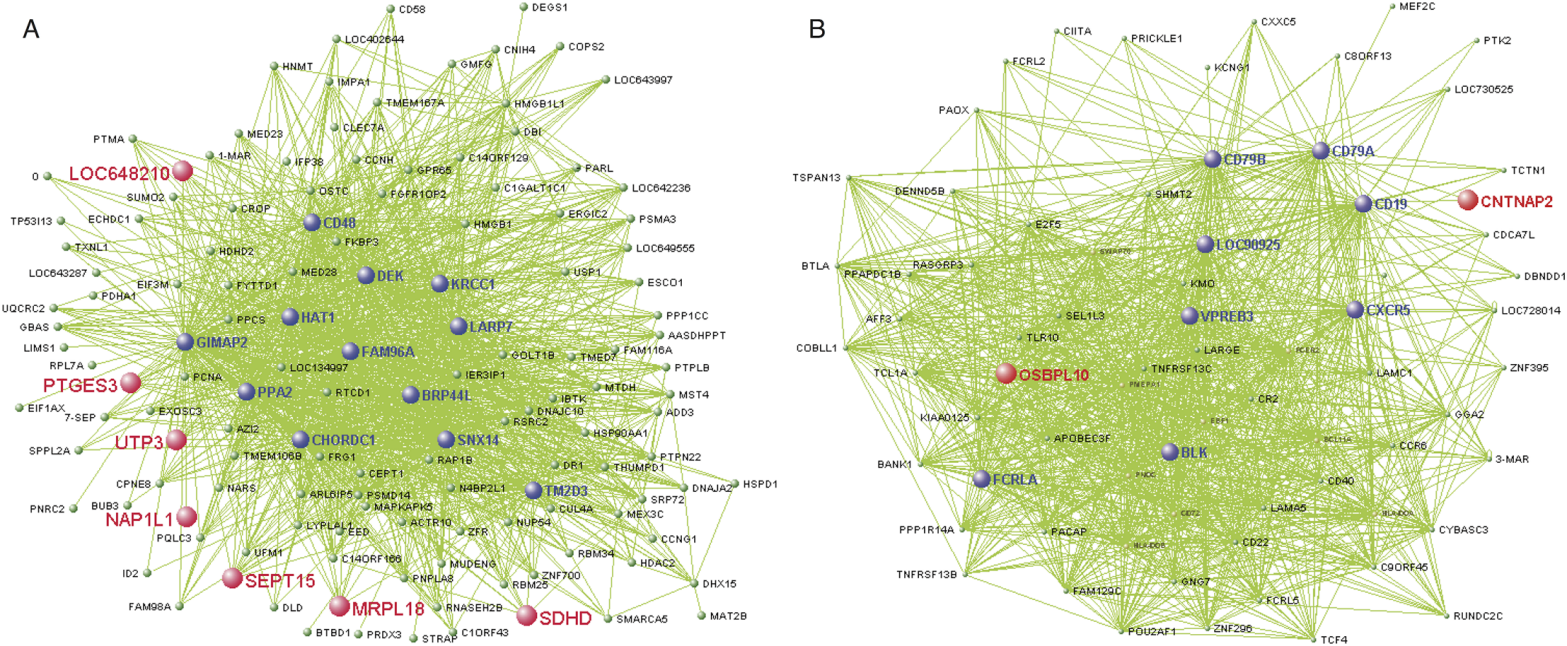
A) Visant plot of top 1500 gene connections in 22q11DS Purple module, which is significantly associated with a psychotic disorder (*p* = .03) and positive symptoms (*p* = 5.0e-8). Genes that are the most strongly connected are listed in a larger font than less connected genes. Genes found in the Purple module that overlap with genes in the Salmon module, which was validated in a set of antipsychotic naive schizophrenia patients module [51] are highlighted in red. B) Visant plot of top 1500 gene connections in 22q11DS Royal Blue module, which is significantly associated with an ASD diagnosis. Genes that are most strongly connected are listed in larger font than other, less connected genes. Genes found in the Royal Blue module that overlap with genes in M12 autism-related brain expressed module [52] are highlighted in red.

We identified one WGCNA module (Purple, 237 probes; Fig 5A, S5 Table) that was associated with psychosis, using both categorical (diagnosis status, *r* = .32, *p* = .03) and dimensional measures of psychotic symptomatology (total SIPS positive symptoms, *r* = .54, *p* = 1.0e-4). Genes co-expressed within this module were predominantly up-regulated in 22q11DS-PSY+, and in 22q11DS subjects with greater positive symptom severity. This module was not associated with antipsychotic medication use (*r* = .04, *p* = .80). Seventy-five percent of the probes in this module were brain-expressed (hypergeometric test, *p* = 1.92e-19) [42]. GO categories (*q≤*.05) associated with this module were related to protein folding, acetyl-co metabolic processes, and aerobic respiration (Fig A in S5 Fig). Pathway analyses revealed that the top network associated with this module was RNA post-transcriptional modification (Fig B in S5 Fig).

#### Autism Spectrum Disorder

One WGCNA module (Royal Blue, 86 probes, Fig 5B, S6 Table) was associated with ASD diagnosis in 22q11DS (*r* = 0.4, *p* = 0.006). Genes in this module were predominantly up-regulated in 22q11DS-ASD+, and were primarily related to immunological processes and functions, according to GO (*q≤*.05) and IPA (S6 Fig). Sixty-two percent of the probes in this module were brain-expressed (hypergeometric test, *p* = .002) [42].

IQ was not significantly associated with the ASD (*r* = .038, *p* = .80) or psychosis modules (*r* = -.26, *p* = .09).

Gender was not significantly associated with the ASD (*r* = 0.046, *p* = 0.8) or psychosis modules (*r* = -0.16, *p* = 0.3)

See S1 Text and S7 Fig for results of the module preservation analysis.

### Validation with published datasets

We observed significant overlap between our psychosis WGCNA module and multiple modules associated with idiopathic schizophrenia in whole blood obtained in a large Dutch sample (Fig 5A,) [58]. One module that significantly overlapped with our Purple module (*p* = 2.7e-07), was also observed in an independent antipsychotic-naïve schizophrenia dataset [58]. Permutation test showed that our observed overlap lies 9 standard deviations from the median of the observed values within the empirical null distribution.

Additionally, we identified overlapping genes between the Royal Blue module and genes related to idiopathic ASD (hypergeometric test, *p* = .01) [61]. Four genes in our ASD module (i.e., *CNTNAP2*, *KMO*, *MEF2C*, *PRICKLE*1) have been previously associated with idiopathic ASD in humans [61]. There was also a trend toward overlap between this module and an autism-related ‘neuronal’ module (M12) from post-mortem brain transcriptome data obtained from idiopathic autism cases (hypergeoemtric test, *p* = 0.10, Fig 5B) [59]. *CNTNAP2*—a gene consistently implicated in the etiology of ASD and specific language impairment [62]-was again identified in both our ASD module and in the M12 module.

## Discussion

In this comprehensive investigation of genomic profiles in 22q11DS patients, we examined structural variability within the 22q11.2 locus, compared peripheral blood gene expression profiles of 22q11DS participants to unaffected controls, and utilized a systems biology approach, WGCNA, to identify genes associated with variability in phenotypic expression in 22q11DS. This is the largest study to date of genome-wide gene expression in phenotypically well-characterized patients with 22q11DS, and the first to utilize an unbiased network-based approach to investigate disrupted functional pathways in 22q11DS. We identified distinct gene expression profiles associated with psychosis and ASD in 22q11DS, which showed significant overlap with genes associated with idiopathic psychosis and ASD. These findings provide support for the notion that investigation of highly penetrant genetic variants of these disorders can guide exploration for disrupted genes and functional pathways relevant to idiopathic forms of ASD and psychosis.

### Structural variability: Deletion breakpoints

Our aCGH results confirmed that the 3 MB deletion is the most common form of the deletion[36,60,63], but also identified additional structural variability within and proximal to the deleted region. Two 22q11DS individuals with the typical 3 MB deletion also had a distal duplication that encompassed one gene, *TOP3B*, a eukaryotic DNA topoisomerase. *TOP3B* is involved in RNA metabolism through its interaction with FMRP [64], which codes for the fragile X mental retardation protein, and inhibits the translation of neuronal mRNAs [65]. Two studies have shown that single nucleotide and copy number variants of *TOP3B* are enriched in individuals with schizophrenia and ASD [66,67]. Recently, deletions of *TOP3B* were shown to be associated with both schizophrenia and intellectual disability [64]. Given the variability documented within our sample, and in the two prior studies to apply high-resolution tiling arrays to the 22q11.2 region [35,36], larger samples are needed to adequately address whether disruption of the region encompassing *TOP3B* is associated with phenotypic variability within 22q11DS.

### Differential Expression Findings

#### 22q11DS vs. Controls

As anticipated, genes within the 22q11.2 locus of deleted patients were significantly down-regulated relative to controls. There was, nevertheless, substantial variability in gene expression levels within 22q11DS patients, which may be partially explained by genetic variability in functional alleles within or proximal to the 22q11.2 locus, and/or regulatory regions [27]. For example, recent findings show that, while there is an approximately 50% reduction in COMT mRNA, protein, and enzyme activity levels in 22q11DS patients overall, COMT haplotypes containing SNPs in the 3’ untranslated region modulate the effect of the Val158Met genotype on COMT expression and enzyme activity [68]. Furthermore, we found that genes that were DE in 22q11DS patients vs. controls showed significant enrichment for brain-expressed genes, and top genetic networks were associated with axon guidance signaling pathways. This finding complements human neuroimaging studies reporting disrupted white matter integrity in 22q11DS, possibly driven by reduced axonal coherence [69,70]. These brain-related alterations may be associated with characteristic phenotypes of 22q11DS (e.g., social cognition and visual-spatial processing deficits) [71].

#### Psychosis

Our GO analyses revealed an over-representation of genes associated with mitochondrial organization and transport in genes DE in 22q11DS patients with a psychotic disorder. Recent studies have implicated mitochondrial dysfunction in the pathophysiology of (idiopathic) schizophrenia [72], as transcriptome alterations in genes associated with mitochondrial functioning has been found in postmortem brain tissue of individuals with schizophrenia [73,74]. However, it is unknown whether mitochondrial dysfunction in schizophrenia is due to a genetic liability, a secondary response to neurotransmitter dysfunction, or a consequence of environmental toxins[75]. Furthermore, there is evidence that mitochondrial dysfunction is also involved in other psychiatric disorders [76]. These findings, paired with the fact that a subset of the genes within the 22q11 region encode mitochondrial proteins [77], suggest that the relationship between mitochondrial dysfunction and psychiatric disorders in those with 22q11DS should be investigated further.

### Weighted Gene Coexpression Network Findings

It is noteworthy that we did not find a significant enrichment of brain expressed genes when we looked at genes differentially expressed in 22q11DS patients with ASD and psychosis, respectively, but the WGCNA modules associated with the clinical phenotypes were significantly enriched for brain expressed genes. This discrepancy is likely due to the different analytic methods used. WGCNA examines the level of co-expression between genes and identifies transcripts that are not up- or down- regulated in the respective psychiatric conditions. Thus, it appears that WGCNA able to identify more brain-related genes associated with the clinical phenotype than the DE analysis.

#### Psychosis

Our results indicate distinct genome-wide transcriptional changes relevant to phenotypic expression of psychotis in 22q11DS patients. We identified a module of functionally connected genes that was associated with categorical and dimensional measures of psychosis. This module showed significant overlap with modules previously associated with idiopathic schizophrenia and was not related to antipsychotic medication use. The top network associated with this module was RNA post-transcriptional modification. Alterations of microRNA expression, which is involved in post-transcription regulation of gene expression, have been identified in post-mortem prefrontal cortical brain tissue of schizophrenia patients. [78,79] Additionally, disruptions in RNA metabolism are a molecular signature common to many neurodevelopmental disorders [80].

Given the emerging body of evidence linking immune system pathways to schizophrenia and psychosis vulnerability [81], it should be noted that we originally identified significant enrichment of genes associated with immune response in the WGCNA psychosis module; however, these terms did not survive correction for multiple comparisons (*q*-value range: .07-.08). Indeed our psychosis module included several immune-related genes, including *TLR7*, which is part of a class of genes called toll-like receptors, whose molecules activate an inflammation response and have been shown to be elevated in peripheral blood of those with psychotic disorders [82]. Recently, suggestive evidence for neuroinflammation in idiopathic schizophrenia has been provided by novel free water diffusion imaging methods indicating increased extracellular volume in patients in a first episode of schizophrenia [83]. Thus, the role of inflammatory processes in the etiology of psychosis, both in 22q11DS and in the broader population, will be important to further investigate in future studies.

#### Autism Spectrum Disorder

Both DE analysis and WGCNA showed converging evidence that alterations in immune-related pathways are associated with ASD. We identified a module of co-expressed genes, significantly associated with ASD, which also contained multiple genes that have previously been associated with idiopathic ASD (*CNTNAP2*, *KMO*, *MEF2C*, *PRICKLE1*[84–87]). Post-mortem studies have identified increased micro- and astro-glial reactions in multiple brain regions in idiopathic ASD [88–90], coupled with up-regulation of pro- and anti-inflammatory cytokines [88]. Together, these findings suggest that ASD pathology may be related to an innate immune system response to neuronal disturbances.

There is debate over whether the ASD and psychosis phenotypes within 22q11DS are considered to be distinct phenomena. [27,30,91]. Our findings of independent gene expression modules associated with psychosis and ASD in 22q11DS support the notion that these two disorders are distinct phenomena. However, an inherent limitation to our cross-sectional study is that we do not know which 22q11DS participants will subsequently develop a psychotic disorder, given the age range of our sample. Inclusion of such ‘false negative’ cases in our non-psychotic group would presumably add noise to our data, reducing our ability to obtain significant results rather than leading to spurious statistically significant findings. Nevertheless, prospective, longitudinal studies are necessary to fully address this question.

It should be noted that there is debate over the prevalence of ASD diagnosis in 22q11DS. Though a high prevalence of ASD in 22q11DS has been reported in multiple publications (~14–50%, [14–17]), others have suggested that the incidence of ASD (strictly defined) is not elevated [92]. Some researchers have proposed that early social dysfunction in 22q11DS is associated with premorbidity to schizophrenia, and that these social impairments may result in a misdiagnosis of an ASD [27,91]. However, other studies indicate that a probable childhood diagnosis of ASD does *not* predict later psychotic disorder [30]. Certain methodological approaches, such as reliance only upon collateral report, may inflate the actual number of individuals diagnosed with ASD [92]. Direct comparison of children with idiopathic ASD versus youth with 22q11DS and an ASD diagnosis (using the ADI-R), found that certain features characteristic of idiopathic autism, such as idiosyncratic speech and social reciprocity deficits, were spared in 22q11DS-ASD+ [93]. However, there were also many similarities between the two groups, as they both exhibited impaired non-verbal social communication, inability to use make-believe during play, motor stereotypies and repetitive use of objects, rituals, and difficulties with peer relationships [93]. It is most likely that children with 22q11DS share some phenotypic features with idiopathic ASD, but have a unique behavioral phenotype that does not fit neatly into defined diagnostic boundaries [25]. Future prospective studies that use information from multiple sources (participants and collateral) and examine the relationships between identified ASD symptoms and other psychopathology over time are necessary to determine the validity and stability of ASD diagnoses in 22q11DS.

### Limitations

Several limitations to the current study should be noted. Although brain tissue from early stages of disease might be optimal, it is not available in patients with 22q11DS, and most pathological specimens are from deceased individuals with long disease duration. Whole-blood gene expression profiles have been found to share significant similarities, but not perfect correspondence, with those of multiple CNS tissues [94–97]. Given that there are no publicly available 22q11DS post-mortem databanks, using peripheral blood to examine gene expression is an important first step. Although peripheral blood cannot replace CNS tissue, it may provide a suitable surrogate, particularly when post-mortem brain tissue is not available. Finally, more consistent and reproducible effects on gene expression are likely to be obtained in patients with homogeneous genetic etiology(4) like 22q11DS.

The number of subjects in our dataset with psychosis in particular was small and severel of the analyses were not corrected for multiple comparisons; additional datasets of peripheral blood and other tissues from 22q11DS patients are needed to confirm these findings. To maximize the sample size for our primary analyses, we chose to include all available 22q11DS patients and healthy controls. Thus, not all of our samples were independent of each other. However, results of a secondary analysis (22q11DS cases vs. a subset of age-matched, unrelated controls; N = 39) was highly comparable to the overall analysis, indicating that our primary results were not attributable to non-independence in the control group. However, age effects on gene expression, both within and outside of the 22q11.2 locus, warrant further systematic investigation.Future analyses may also benefit from using more sensitive technology (i.e., RNA sequencing) to identify splicing events, non-coding RNAs, and novel transcripts [98,99].

### Conclusion

Collectively, these findings provide a first step toward understanding the functional gene networks disrupted by the 22q11.2 deletion, which may relate to variable phenotypic expression of the disorder. Future investigations in human *in vitro* cellular models and in animal models of 22q11.2 mutations are necessary to link the affected genetic pathways to molecular mechanisms, which may be targeted for biologically informed interventions.

## Supporting Information

S1 Text Methods for CGH and WGCNA analyses, results of secondary analyses conducted. (DOC)

S1 R Code R code for WGCNA analysis. (DOCX)

S1 Fig Box plots of genes of genes in the 22q11.2 locus with significant differential expression levels in 22q11DS patients relative to controls. (DOCX)

S2 Fig Top network associated with differentially expressed genes in 22q11DS-PSY+ (*p* < .005). (DOCX)

S3 Fig Top network associated with differentially expressed genes in 22q11DS-ASD+ (*p* < .005). (DOCX)

S4 Fig Relationship between COMT gene expression level and age in controls (*r* = .27, *p* = .027). (DOCX)

S5 Fig Gene ontology (GO) analysis and Ingenuity Pathway Analysis for the Purple module. (DOCX)

S6 Fig Gene ontology analysis and Ingenuity Pathway Analysis for the Royal Blue module. (DOCX)

S7 Fig Module preservation analysis. (DOCX)

S8 Fig Top network associated with differentially expressed genes in 22q11DS-PSY- vs. controls (*p* < .005). (DOCX)

S9 Fig Top network associated with differentially expressed genes in 22q11DS-PSY+ vs. controls (*p* < .005). (DOCX)

S1 Table Psychotropic medication information for 22q11DS patients. (DOCX)

S2 Table Differentially expressed probes (significant at 5% FDR) in 22q11DS (N = 46) vs. controls (N = 66). (DOCX)

S3 Table Differentially expressed probes (significant at 5% FDR) in 22q11DS-PSY+ (N = 6) vs. 22q11DS-PSY- (N = 40). (DOCX)

S4 Table Differentially expressed probes significant (at 5% FDR) in 22q11DS-ASD+ (N = 15) vs. 22q11DS-ASD- (N = 25). (DOCX)

S5 Table List of genes in the Purple module, which was significantly associated with psychotic disorder diagnosis in 22q11DS. (DOCX)

S6 Table List of genes in the Royal Blue module, which was significantly associated with an ASD diagnosis in 22q11DS. (DOCX)
