# Supplementary material for: Transcriptome Profiling of Peripheral Blood in 22q11.2 Deletion Syndrome Reveals Functional Pathways Related to Psychosis and Autism Spectrum Disorder
Source: PLoS One. 2015 Jul 22;10(7):e0132542. doi: 10.1371/journal.pone.0132542 (PMC4511766; doi:10.1371/journal.pone.0132542)
Supplement: S1 Text — (DOC) [file pone.0132542.s001.doc]

**S1 Text**

**Methods**

**Comparative Genomic Hybridization Arrays**

The region from chr22:14.7-23.5 MB, which includes the typical 3-MB deletion as well as LCRs, was tiled at high density with a mean probe spacing of ~50bp (total number of probes in this region: 110,148). The rest of the probes on the array spanned the entire chromosome, resulting in a final tiling path of about 1167bp. Only probes that mapped a maximum of 10 times to the hg19 reference build were included, and no replicate probes were used in the design.

A total of fifty-seven samples (46 22q11DS, and 11 of their unaffected first-degree relatives; see Table S1 for demographics) were submitted to Roche NimbleGen for aCGH service. Patient genomic DNA was fragmented and labeled with Cy3, and hybridized to the custom array alongside a Cy5 labeled, pooled female reference from NimbleGen. Samples from the same family were assayed on the same array (Table S2). Calling of CNV regions was performed using the segMNT algorithm included in NimbleScan v. 2.6 software. All called CNV regions were manually inspected and were found to be consistent amongst technical replicates.

The typically deleted 3MB region on chr22 is bookended by four LCRs denoted as LCR A-D [1]. Although to our knowledge, the density our tiling array design is the highest of any reported in the literature, the nature of these LCR regions prohibits a precise breakpoint delineation that is inherent to the technology. Therefore, in order to unequivocally classify the samples into distinct deletion categories, all non-uniquely aligning probes were removed prior to assessment.

**WGCNA**

Prior to running the WGCNA analysis, the data was normalized, corrected for batch effects, and the effects of age were regressed out. We calculated the standard deviation for each transcript and selected the 8000 most variable genes for WGCNA analysis. Using the R function picksoftThreshold, we conducted an analysis of scale free topology[2]. The soft threshold was identified as the lowest beta value (power of 12) that resulted in an approximation of scale free topology. Using this power, we calculated a weighted adjacency matrix of a signed network (R function adjacency). After calculating the topological overlap (R function TOMsimilarity), we then calculated a dissimilarity matrix. Hierarchical clustering was conducted using the R package flashClust. After specifying a minimum module size of 30, we used the dynamic tree cutting method in the cuttreeDynamic R function [3] to identify modules. Using the R function moduleEigengenes, we calculated the first principal component of each module (module eigengene). Finally, we conducted Pearson correlations between the module eigengenes and phenotypic traits of interest. The R code used for the WGCNA analysis can be found in S1 R Code.

**Results**

**Age Relationships**

Using a threshold of *p*<.05, 13% of the probes were significantly associated with age in each group (controls: 4997, 22q11DS participants: 5051)in these two groups. Of note, there was a significant correlation between COMT gene expression levels and age in controls (*r*=.27, *p*=.03, Figure S2), with COMT gene expression levels increasing with age. This finding replicates a previous study reporting that COMT enzyme activity in the prefrontal cortex increases across postnatal human development (from neonate to adulthood) in postmortem brain tissue, which supports the investigation of gene expression profiles in peripheral blood as a proxy for brain tissue [4].

**Secondary gene expression analyses**

**22q11DS vs. Unaffected Relatives Only**

Since a subset of our controls were biological relatives of 22q11DS patients, we compared patients vs. their unaffected family members in a paired design, and obtained highly overlapping results to those obtained in our overall analysis (fold change correlation coefficient between analysis with all controls vs. family members only: *r*=.975 , *p*=2.2e-16).

**22q11DS vs. Age-Matched Controls Only**

We also conducted an additional analysis in which we included only the subset of age-matched controls (N=39) from our overall control sample. We re-ran the DE analysis and obtained highly overlapping results to those found in our original analysis, with a fold-change correlation coefficient between the original analysis and the age-matched control analysis of r=.91 (p<2.2e-16). Additionally, 90% of the genes that were DE in the original analysis were also DE in the analysis comparing 22q11DS patients to age-matched controls only.

**22q11DS vs. Controls: Correcting for RNA Integrity Number (RIN)**

We also conducted an analysis in which we regressed out RIN and then re-ran the DE. We obtained highly consistent results to those found in our original analysis, with a fold change correlation coefficient between the two analyses of *r*=.98 (*p*<2.2e-16).

**22q11DS vs. Controls: Correcting for Gender**

We also conducted an analysis in which we regressed out gender and then re-ran the DE. We obtained highly consistent results to those found in our original analysis, with a fold change correlation coefficient between the two analyses of *r*=.77 (*p*<2.2e-16).

**22q11DS-PSY- vs. Controls**

Three hundred ninety-nine probes were DE (254 down-regulated, 145 up-regulated) in 22q11DS without psychosis (22q11DS-PSY-) vs. controls (*p*<.005, uncorrected). There was a high degree of overlap between results from this analysis and the original DE analysis of the overall 22q11DS sample vs. controls: 314 (79%) of the probes DE in the 22q11DS-PSY- vs. controls analysis were also DE in the entire 22q11DS sample vs. controls. Not surprisingly, GO analyses of genes DE in 22q11DS-PSY- vs. controls were also very similar, with top GO categories (*q≤.* 05) including of regulation of neuronal action potentials, myelination, and axon ensheathment of neurons. The top IPA network of these DE genes was associated with cell death and survival (S8 Figure).

**22q11DS-PSY+ vs. Controls**

One hundred eighty probes were DE (98 down-regulated, 82 up-regulated) in 22q11DS patients with psychosis (22q11DS-PSY+) vs. controls (p<.005, uncorrected). Sixty-three probes (35%) that were DE in the above analysis were also DE expressed in the DE analysis of the overall 22q11DS sample vs. controls. No GO terms survived after correction for multiple comparisons (*q≤*.05). IPA revealed that the top network of these DE genes was associated with immunological disease and cellular movement (S9 Figure).

**Module preservation results**

After removing the 3 22q11DS participants with diagnoses of both a psychotic disorder and ASD, we ran module preservation statistics. Z-statistic values indicated that all modules were preserved when WGCNA was re-run without these individuals (S9 Fig). For the Purple (psychosis) and Royal Blue (ASD) modules, the Z-statistic summary values were 26 and 19, respectively, indicating that both modules were highly preserved.

**Supplementary References**

1. Edelmann L, Pandita RK, Morrow BE (1999) Low-copy repeats mediate the common 3-Mb deletion in patients with velo-cardio-facial syndrome. Am J Hum Genet 64: 1076–1086.

2. Zhang B, Horvath S (2005) A general framework for weighted gene co-expression network analysis. Stat Appl Genet Mol Biol 4: Article17. doi:10.2202/1544-6115.1128.

3. Langfelder P, Horvath S (2008) WGCNA: an R package for weighted correlation network analysis. BMC Bioinformatics 9: 559. doi:10.1186/1471-2105-9-559.

4. Tunbridge EM, Weickert CS, Kleinman JE, Herman MM, Chen J, et al. (2007) Catechol-o-methyltransferase enzyme activity and protein expression in human prefrontal cortex across the postnatal lifespan. Cereb Cortex 17: 1206–1212. doi:10.1093/cercor/bhl032.
