# Supplementary material for: Transcriptome Profiling of Peripheral Blood in 22q11.2 Deletion Syndrome Reveals Functional Pathways Related to Psychosis and Autism Spectrum Disorder
Source: PLoS One. 2015 Jul 22;10(7):e0132542. doi: 10.1371/journal.pone.0132542 (PMC4511766; doi:10.1371/journal.pone.0132542)
Supplement: S1 R Code — (DOCX) [file pone.0132542.s002.docx]

**R Code for WGCNA Analysis**

#these libraries need to be installed prior to running the code

library(WGCNA)

library(cluster)

library (limma)

library(car)

#set your working directory where you want the data to be saved

setwd("/Users/mariaj/Dropbox/R/Bearden_22q11DS_Microarray1/WGCNA/patients/age_out/ageout_sd")

#read in sample data (total sample)

samples<-read.delim("/Users/mariaj/Dropbox/R/Bearden_22q11DS_Microarray1/Samples.gg.age.txt", header=T, sep="\t")

colnames(samples)

#want to extract out patients only

patient_sample<-samples[samples$Diagnosis =="patient",]

dim(patient_sample)

#WGCNA patients only

#read in data that has outliers removed, technical replicates averaged, normalized, corrected for batch effects

dat1<-read.delim("/Users/mariaj/Dropbox/R/Bearden_22q11DS_Microarray1/WGCNA/patients/age_out/patients_ageout_residuals.txt",header=T,sep=" ")

#calculate overall SD for each probe

dat_sd<-apply(dat1,1,sd)

#add SD to data

dat2<-cbind(dat_sd,dat1)

#now put them in order with most variable at top

dat3<-dat2[order(dat2$dat_sd, decreasing=TRUE),]

#take out the sorting column

dat4<-dat3[,2:47]

#select 8000 top variable genes

dat5<-dat4[1:8000,]

#get dimensions of data set

dim(dat5)

#transpose the data because WGCNA needs the data with probes in columns and samples in rows

datExpr= data.frame(t(dat5))

dim(datExpr)

#choose soft-thresholding power

pdf("bearden_power1.pdf", height=10, width=18)

# Choose a set of soft-thresholding powers

powers = c(c(1:10), seq(from = 12, to=20, by=2))

# Call the network topology analysis function

#we want to pick a soft threshold that satisfies scale-free topology

sft = pickSoftThreshold(datExpr, powerVector = powers, verbose = 5)#networkType="signed"

# Plot the results:

#two graphs side by side

par(mfrow = c(1,2));

cex1 = 0.9;

# Scale-free topology fit index as a function of the soft-thresholding power

plot(sft$fitIndices[,1], -sign(sft$fitIndices[,3])*sft$fitIndices[,2],

xlab="Soft Threshold (power)",ylab="Scale Free Topology Model Fit,signed R^2",type="n",

main = paste("Scale independence"));

text(sft$fitIndices[,1], -sign(sft$fitIndices[,3])*sft$fitIndices[,2],

labels=powers,cex=cex1,col="red");

# this line corresponds to using an R^2 cut-off of h

abline(h=0.90,col="red")

# Mean connectivity as a function of the soft-thresholding power

plot(sft$fitIndices[,1], sft$fitIndices[,5],

xlab="Soft Threshold (power)",ylab="Mean Connectivity", type="n",

main = paste("Mean connectivity"))

text(sft$fitIndices[,1], sft$fitIndices[,5], labels=powers, cex=cex1,col="red")

dev.off()

#based on above plot we chose

softPower = 12;

#creates connection strengths between pairs of genes

#calculates (either correlation or distance) network adjacency from given expression data

#input gene expression data (datExpr)

#power means to specify your soft thresholding power

#type= network type, it can be usigned, signed, or signed hybrid

#in correlation network, the adjacency is contructed from the correlations (values between -1 and 1, high numbers meaning high similarity)

adjacency = adjacency(datExpr, power = softPower, type = "signed");

#this is your correlation matrix

dim(adjacency)

# Turn adjacency into topological overlap

#this is a measurement of the true interconnectedness

TOM = TOMsimilarity(adjacency);

dissTOM = 1-TOM

geneTree = flashClust(as.dist(dissTOM), method = "average");

#plot it

pdf("1-Gene clustering.pdf", height=10, width=15)

#sizeGrWindow(12,9)

plot(geneTree, xlab="", sub="", main = "Gene clustering on TOM-based dissimilarity",

labels = FALSE, hang = 0.04);

dev.off()

#specify minimum module size

minModuleSize = 30;

dynamicMods = cutreeDynamic(dendro = geneTree, distM = dissTOM, cutHeight = 0.995,

deepSplit = 2, pamRespectsDendro = FALSE,minClusterSize = minModuleSize, verbose = 2);

table(dynamicMods)

# Convert numeric lables into colors

dynamicColors = labels2colors(dynamicMods)

table(dynamicColors)

# Plot the dendrogram and colors underneath

pdf("2-Gene dendrogram and module colors.pdf", height=10, width=15)

plotDendroAndColors(geneTree, dynamicColors, "Dynamic Tree Cut",

dendroLabels = FALSE, hang = 0.03,

addGuide = TRUE, guideHang = 0.05,

main = "Gene dendrogram and module colors")

dev.off()

# Calculate eigengenes

MEList = moduleEigengenes(datExpr, colors = dynamicColors,softPower= softPower, nPC=1)

MEs = MEList$eigengenes

# Calculate dissimilarity of module eigengenes

MEDiss = 1-cor(MEs);

# Cluster module eigengenes

METree = flashClust(as.dist(MEDiss), method = "average");

# Plot the result

pdf("3-Clustering of module eigengene.pdf", height=10, width=15)

plot(METree, main = "Clustering of module eigengenes",

xlab = "", sub = "")

# We choose a height cut of 0.2, corresponding to correlation of 0.8

MEDissThres = 0.2

# Plot the cut line into the dendrogram

abline(h=MEDissThres, col = "red")

dev.off()

MEDissThres = 0.05

# Call an automatic merging function to merge any close modules that should be joined as one

#in this example none of our modules were so similar that they needed to be merged together

merge = mergeCloseModules(datExpr, dynamicColors, cutHeight = MEDissThres, verbose = 3)

# merged module colors

mergedColors = merge$colors;

# igengenes of the new merged modules:

mergedMEs = merge$newMEs;

pdf("4-Clustering of module eigengene.pdf", height=10, width=15)

plotDendroAndColors(geneTree, cbind(dynamicColors, mergedColors),

c("Dynamic Tree Cut", "Merged dynamic"),dendroLabels = FALSE, hang = 0.03,

addGuide = TRUE, guideHang = 0.05)

dev.off()

# Rename mergedColors to moduleColors

moduleColors = mergedColors

#starting with grey please make a list of 50 standard colors

colorOrder = c("grey", standardColors(50));

#now assign a numerical value to each color, except don't do it for grey

moduleLabels = match(moduleColors, colorOrder)-1;

moduleLabels

#mergedMEs now defined as ME

MEs=mergedMEs;

#now we want to calculate the intramodular connectivity, how well specific genes are connected to other genes within same module

#need our adjacency matrix (adjacency) & the list of colors (moduleColors)

Alldegrees1=intramodularConnectivity(adjacency, moduleColors)

head(Alldegrees1)

rownames(MEs)<-rownames(datExpr)

write.table(MEs, file="patient_ME_output.csv",sep=",")

#read in annotation file

#load annotation file

annot <- read.delim(file="/Users/mariaj/Dropbox/R/Bearden_22q11DS_Microarray1/HumanHT-12_V4_0_R2_15002873_B.txt", header=T)

dim(annot)

colnames(annot)

#need to match up the annotation info we need

rownames(dat5)

match_probes<-match (rownames(dat5), annot$Probe_Id)

match_probes

annot_8000<-annot[match_probes,]

geneInfo0<-data.frame(Probe=rownames(dat5),

Accession=annot_8000$Accession,

Symbol=annot_8000$Symbol,

Definition=annot_8000$Definition,

Chromosome=annot_8000$Chromosome,

Cytoband=annot_8000$Cytoband,

moduleColor=moduleColors,

meanExpr=apply(datExpr,2,mean),

Alldegrees1)

write.csv(geneInfo0, file = "patient_ke_Info.csv")

#this shows how correlated the modules are with each other

pdf("5-eigengenes.pdf",height=10,width=18)

par(cex = 0.7)

plotEigengeneNetworks(MEs, "", marDendro = c(0,4,1,2), marHeatmap = c(3,4,1,2), cex.adjacency = 0.3 , cex.preservation = 0.3, plotPreservation = "standard")

dev.off()

#write out final modules

write.table(table(moduleColors), file="final_modules.csv", sep=",")

PCvalues<-MEs #exclude grey

modules<-colnames(PCvalues)

#look for the first two characters "ME" and take it out of the module names

colorsMod<-gsub("ME","",modules)

#need sample names

namesSamp<-as.character(rownames(datExpr))

pdf(file="patient_PC.pdf",width=15,height=10)

plot(smooth.spline(PCvalues[,1],spar=0.4), xlab="", type="n", ylim=c(-0.4, 0.4),ylab="First Principal Component", axes=F, main=" Modules")

for (mod in 1:length(PCvalues)) {

lines(smooth.spline(PCvalues[,mod],spar=0.4), col=colorsMod[mod], lwd=4)

}

abline(h=0)

axis(1,at=1:length(namesSamp),labels=namesSamp,cex.axis=0.4,las=3)

axis(2, at=seq(-0.4, 0.4, 0.1))

box()

legend("bottomright", colorsMod,fill=colorsMod, cex=0.5)

dev.off()

#single plots

pdf(file="patient_singlePC.pdf",width=25,height=13)

par(mfrow=c(5,6))

for (mod in 1:length(PCvalues)) {

plot(smooth.spline(PCvalues[,mod],spar=0.4), xlab="", type="n", ylim=c(-0.4, 0.4),ylab="First Principal Component", axes=F, main= colorsMod[mod])

polygon(x=c(12,27,27,12),y=c(-0.4,-0.4,0.4,0.4), col="lavender",border="lavender")

#polygon(x=c(152,230,230,152),y=c(-0.4,-0.4,0.4,0.4), col="lavender",border="lavender")

abline(h=0)

lines(smooth.spline(PCvalues[,mod],spar=0.4), col=colorsMod[mod], lwd=4)

axis(1,at=1:length(namesSamp),labels=namesSamp,cex.axis=0.7,las=3)

axis(2, at=seq(-0.4, 0.4, 0.1))

#box()

}

dev.off()

##heat

library(gplots)

library(sma)

treat<-c(rep("magenta",11), rep("blue",16))

pdf(file="black.pdf",width=21,height=12)

#setting up variable

whichmodule="black"

#create a matrix of plots, in 2 rows and 2 columns

par(mfrow=c(2,1))

#setting the margins of the plot

par(mar=c(3.5,3,2.5,3))

#gives outer margins of plot

par(oma=c(4,0,2,0))

#we want the columns of the data that have the module color we specified

#module colors is a list of each genes module that they are in

datcombined=datExpr[,moduleColors==whichmodule]

plot.mat(t(scale(datcombined)),main="black")

names=dimnames(datExpr)[[1]]

datsv=MEs$MEblack

whichmodule="black"

xpos=seq(0.5,26.5,1.0)

barplot(datsv,col=whichmodule, xlim=c(1.1,26),width=0.85,space=0.174)

axis(side=1,at=xpos,labels=F,tick=T)

box()

mtext(names,1,at=xpos,cex=0.7, col=as.vector(treat), adj=1.2,las=3)

dev.off()

#reading in trait data

traitdata<-patient_sample

dim(traitdata)

colnames(traitdata)

#only select the traits I'm interested in

traitdata2<-traitdata[,c(9,10,16,18,20,21)]

#recode M and F as numbers so they can run thru WGCNA

traitdata2$Sex<-recode(traitdata2$Sex,"'M'=1; 'F'=0;", as.factor.result=FALSE)

#doing the actual correlations

nGenes = ncol(datExpr);

nSamples = nrow(datExpr);

moduleTraitCor = cor(MEs, traitdata2, use = "p");

moduleTraitPvalue = corPvalueStudent(moduleTraitCor, nSamples);

colnames(moduleTraitPvalue) = paste("p.value.", colnames(moduleTraitCor), sep="");

out3<-cbind(Module=rownames(moduleTraitCor ), moduleTraitCor, moduleTraitPvalue)

dim(out3)

#write out correlations

write.table(out3, "patient_moduleTraitCor.csv", sep=",",row.names=F)

#plot table of module trait relationships

pdf("module-trait_relationships.pdf", height=10, width=15)

textMatrix = paste(signif(moduleTraitCor, 2), "\n(",

signif(moduleTraitPvalue, 1), ")", sep = "")

dim(textMatrix) = dim(moduleTraitCor)

par(mar = c(6, 8.5, 3, 3))

# Display the correlation values within a heatmap plot

labeledHeatmap(Matrix = moduleTraitCor, xLabels = names(traitdata2),

yLabels = names(MEs), ySymbols = names(MEs),

colorLabels =FALSE,colors=greenWhiteRed(50),textMatrix=textMatrix,

setStdMargins = FALSE, cex.text = 0.5, zlim = c(-1,1),

main = paste("Module-trait relationships"))

dev.off()
