# Supplementary material for: Transcriptome Profiling of Peripheral Blood in 22q11.2 Deletion Syndrome Reveals Functional Pathways Related to Psychosis and Autism Spectrum Disorder
Source: PLoS One. 2015 Jul 22;10(7):e0132542. doi: 10.1371/journal.pone.0132542 (PMC4511766; doi:10.1371/journal.pone.0132542)
Supplement: S3 Fig — (DOCX) [file pone.0132542.s005.docx]

**S3 Fig. Top network associated with differentially expressed genes in 22q11DS-ASD+ (*p*<.005).** Ingenuity Pathway analysis revealed a significant network related to immune response in differentially expressed genes in 22q11DS-ASD+ (*p*<.005). Genes in red are up-regulated and genes in green are down-regulated.
