# Supplementary material for: Transcriptome Profiling of Peripheral Blood in 22q11.2 Deletion Syndrome Reveals Functional Pathways Related to Psychosis and Autism Spectrum Disorder
Source: PLoS One. 2015 Jul 22;10(7):e0132542. doi: 10.1371/journal.pone.0132542 (PMC4511766; doi:10.1371/journal.pone.0132542)
Supplement: S4 Fig — (DOCX) [file pone.0132542.s006.docx]

**S4 Fig. Relationship between COMT gene expression level and age in controls (*r*=.27, *p*=.027).**
