# Supplementary material for: Transcriptome Profiling of Peripheral Blood in 22q11.2 Deletion Syndrome Reveals Functional Pathways Related to Psychosis and Autism Spectrum Disorder
Source: PLoS One. 2015 Jul 22;10(7):e0132542. doi: 10.1371/journal.pone.0132542 (PMC4511766; doi:10.1371/journal.pone.0132542)
Supplement: S5 Fig — (DOCX) [file pone.0132542.s007.docx]

**S5 Fig. Gene ontology (GO) analysis and Ingenuity Pathway Analysis for the Purple module.** A) GO analysis (*q*<.05) for Purple (psychosis) module, indicating the GO categories associated with this module are related to protein folding, acetyl-co metabolic processes, and aerobic respiration. B) Ingenuity Pathway analysis revealed a significant over-representation of genes relevant to gene expression and RNA post-transcriptional modification in this module. Genes co-expressed within this module were predominantly up-regulated in those with 22q11DS and a psychotic disorder diagnosis.

A**
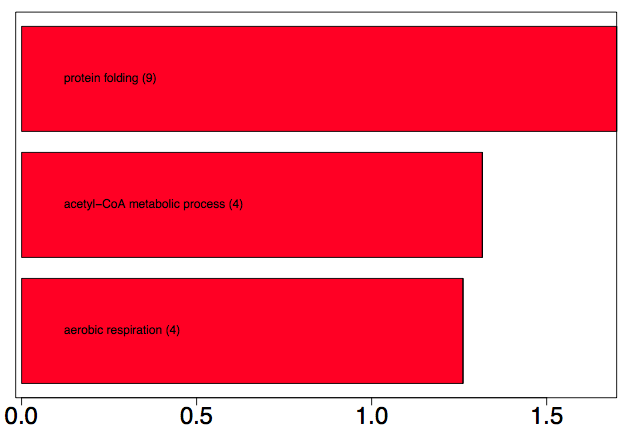
**

**-Log (*q*-value)**

**
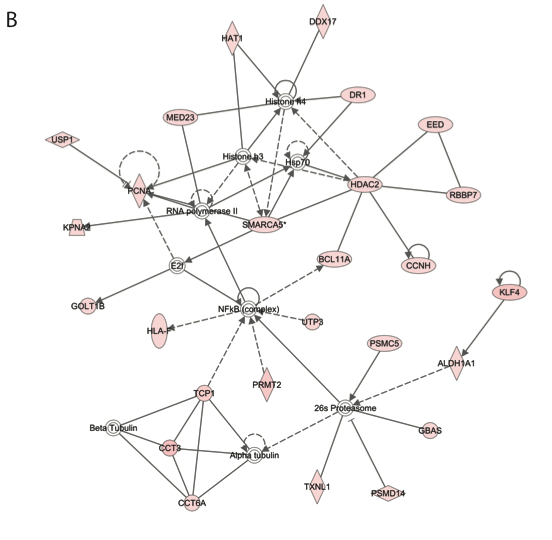
**
