# Supplementary material for: Transcriptome Profiling of Peripheral Blood in 22q11.2 Deletion Syndrome Reveals Functional Pathways Related to Psychosis and Autism Spectrum Disorder
Source: PLoS One. 2015 Jul 22;10(7):e0132542. doi: 10.1371/journal.pone.0132542 (PMC4511766; doi:10.1371/journal.pone.0132542)
Supplement: S6 Fig — (DOCX) [file pone.0132542.s008.docx]

**S6 Fig**. **Gene ontology analysis and Ingenuity Pathway Analysis for the Royal Blue module.** A) Gene ontology analysis (*q*<.05) for Royal Blue (ASD) module, B) Ingenuity Pathway analysis revealed significant over-representation of genes involved with immunological processes, protein synthesis, and cell-to-cell signaling in this module. Genes co-expressed within this module were predominantly up-regulated in those with 22q11DS and an ASD.

A.

-Log (*q*-value)

**
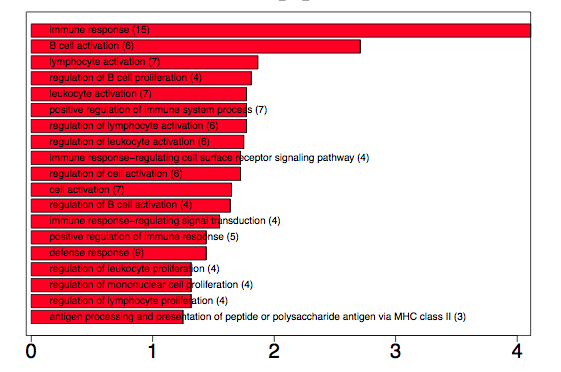
**

**
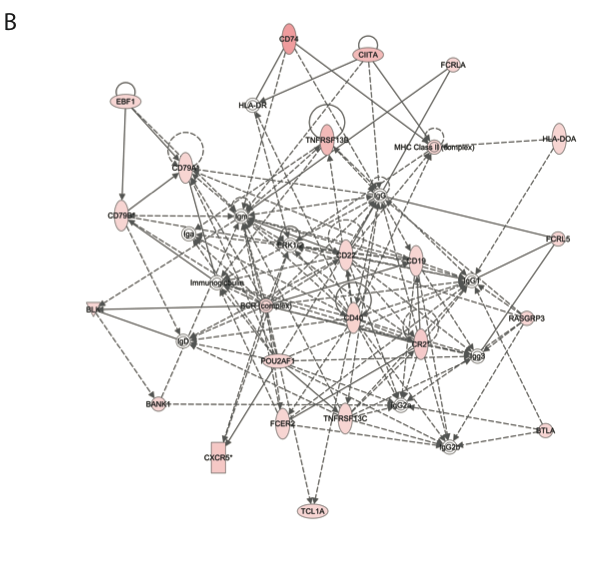
**
