# Supplementary material for: Transcriptome Profiling of Peripheral Blood in 22q11.2 Deletion Syndrome Reveals Functional Pathways Related to Psychosis and Autism Spectrum Disorder
Source: PLoS One. 2015 Jul 22;10(7):e0132542. doi: 10.1371/journal.pone.0132542 (PMC4511766; doi:10.1371/journal.pone.0132542)

**S7 Fig. Module preservation analysis.** Preservation of modules between the original WGCNA dataset of 22q11DS patients (N=46) and subset of this dataset, with 22q11DS patients with comorbid diagnoses of psychosis and ASD removed (N=3). A co-expression network for the subset (N=43) was constructed, using module assignments of the original dataset. The preservation Z-statistic is substantially larger than 5 for all modules (dotted line), indicating significant module preservation between datasets.


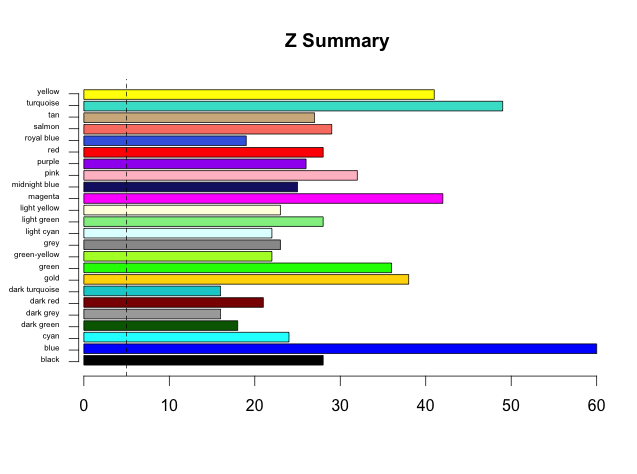

Supplement: S7 Fig — (DOCX) [file pone.0132542.s009.docx]
