# Supplementary material for: Transcriptome Profiling of Peripheral Blood in 22q11.2 Deletion Syndrome Reveals Functional Pathways Related to Psychosis and Autism Spectrum Disorder
Source: PLoS One. 2015 Jul 22;10(7):e0132542. doi: 10.1371/journal.pone.0132542 (PMC4511766; doi:10.1371/journal.pone.0132542)

**S8 Fig. Top network associated with differentially expressed genes in 22q11DS-PSY- vs. controls (*p*<.005).** Ingenuity Pathway analysis revealed a significant network related to cell death and survival in differentially expressed genes in 22q11DS-PSY- (*p*<.005). Genes in red are up-regulated and genes in green are down-regulated.


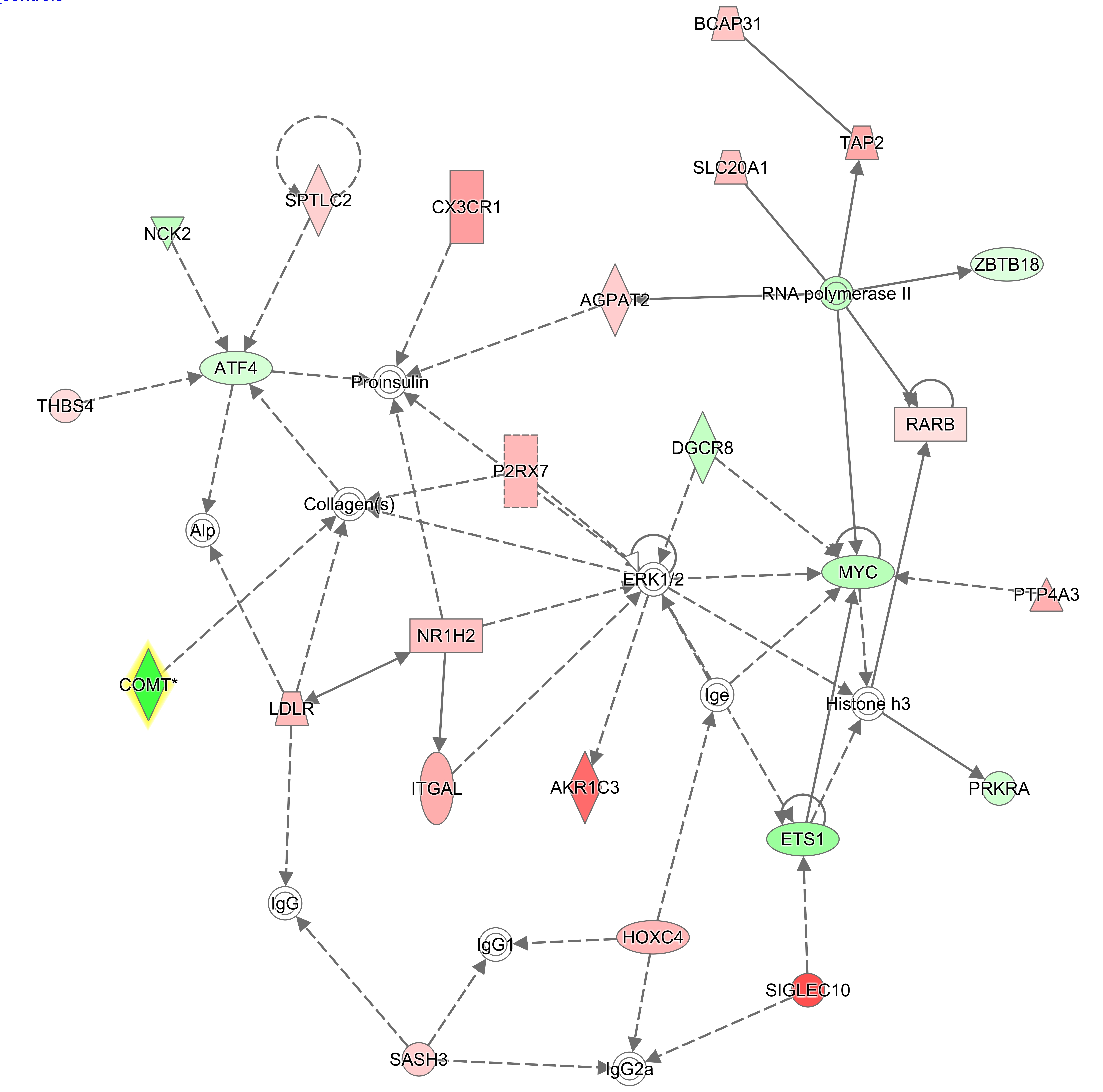

Supplement: S8 Fig — (DOCX) [file pone.0132542.s010.docx]
