# Supplementary material for: Transcriptome Profiling of Peripheral Blood in 22q11.2 Deletion Syndrome Reveals Functional Pathways Related to Psychosis and Autism Spectrum Disorder
Source: PLoS One. 2015 Jul 22;10(7):e0132542. doi: 10.1371/journal.pone.0132542 (PMC4511766; doi:10.1371/journal.pone.0132542)
Supplement: S1 Table — (DOCX) [file pone.0132542.s012.docx]

**S1 Table. Psychotropic medication information for 22q11DS patients.**

| **Medication** | **22q11DS Patients, N (%)** |
| --- | --- |
| No psychotropic medications | 33 (72%) |
| Antipsychotic | 3 (6.5%) |
| Antidepressant | 3 (6.5%) |
| Stimulant | 2 (4%) |
| Two or more different classes of psychotropic medications | 5 (11%) |
| Total Individuals taking antipsychotic medications | 5 (11%) |
