# Supplementary material for: Transcriptome Profiling of Peripheral Blood in 22q11.2 Deletion Syndrome Reveals Functional Pathways Related to Psychosis and Autism Spectrum Disorder
Source: PLoS One. 2015 Jul 22;10(7):e0132542. doi: 10.1371/journal.pone.0132542 (PMC4511766; doi:10.1371/journal.pone.0132542)
Supplement: S2 Table — (DOCX) [file pone.0132542.s013.docx]

**S2 Table. Differentially expressed probes (significant at 5% FDR) in 22q11DS (N=46) vs. controls (N=66).** Column A: Illumina probe name; B: Gene Symbol; C: Gene definition; D: Chromosome location; E: Log2 fold change (22q11DS vs. Controls) in shades of red (51-90^th^ percentile of DE genes, with darkest red =90^th^ percentile) are changes >0.2 (corresponding to an absolute fold change of 1.15), in shades of green (10-49^th^ percentile of DE genes, with darkest green=10^th^ percentile) indicate changes < -0.2 (corresponding to an absolute fold change of 0.87); F) p-value after correction for false discovery rate (FDR) and G) A “**✔** ” is placed next to genes that are considered to be brain expressed(4). Probes that are highlighted in bold and have an asterisk after the Gene Symbol are within the 22q11.2 typically deleted region.

| Probe | Symbol | Gene Name | Chromo-some | Log Ratio | P-Value | Brain Expressed |
| --- | --- | --- | --- | --- | --- | --- |
| **ILMN_1789627** | **SEPT5*** | **septin 5** | **22** | **-0.884** | **<0.000001** | **✔** |
| ILMN_1750636 | RPS26L | PREDICTED: 40S ribosomal protein S26-like |  | -0.655 | 0.00076 |  |
| **ILMN_1789492** | **ZDHHC8*** | **zinc finger, DHHC-type containing 8** | **22** | **-0.63** | **<0.000001** |  |
| **ILMN_2109156** | **RANBP1*** | **RAN binding protein 1** | **22** | **-0.604** | **<0.000001** |  |
| **ILMN_1690122** | **CRKL*** | **v-crk sarcoma virus CT10 oncogene homolog** | **22** | **-0.601** | **<0.000001** | **✔** |
| **ILMN_1705390** | **KLHL22*** | **kelch-like 22** | **22** | **-0.601** | **<0.000001** | **✔** |
| ILMN_1677697 | LOC441377 | PREDICTED: similar to 40S ribosomal protein S26 |  | -0.591 | 0.00037 |  |
| **ILMN_1791396** | **DGCR6*** | **DiGeorge syndrome critical region gene 6** | **22** | **-0.579** | **<0.000001** |  |
| **ILMN_1805161** | **LZTR1*** | **leucine-zipper-like transcription regulator 1** | **22** | **-0.573** | **<0.000001** | **✔** |
| ILMN_2310703 | RPS26L | 40S ribosomal protein S26-like | 13 | -0.555 | 0.0016 |  |
| **ILMN_1730084** | **COMT*** | **catechol-O-methyltransferase** | **22** | **-0.535** | **<0.000001** | **✔** |
| ILMN_2404850 | RPL14 | ribosomal protein L14 | 3 | -0.523 | 0.00219 | ✔ |
| ILMN_1726647 | LOC650646 | PREDICTED: similar to 40S ribosomal protein S26 |  | -0.522 | 0.00222 |  |
| **ILMN_1655177** | **PIK4CA*** | **phosphatidylinositol 4-kinase, catalytic, alpha polypeptide** | **22** | **-0.511** | **<0.000001** |  |
| ILMN_1767281 | PPBP | pro-platelet basic protein | 4 | -0.504 | 0.00065 | ✔ |
| ILMN_2180866 | RPS26P11 | ribosomal protein S26 pseudogene 11 | X | -0.491 | 0.0045 |  |
| **ILMN_1663685** | **DGCR6*** | **DiGeorge syndrome critical region gene 6** | **22** | **-0.488** | **<0.000001** |  |
| ILMN_1678522 | LOC644934 | PREDICTED: similar to 40S ribosomal protein S26 | 15 | -0.475 | 0.00401 |  |
| **ILMN_1721457** | **RANBP1*** | **RAN binding protein 1** | **22** | **-0.472** | **<0.000001** |  |
| **ILMN_1659857** | **SNAP29*** | **synaptosomal-associated protein, 29kDa** | **22** | **-0.427** | **<0.000001** | **✔** |
| **ILMN_1713301** | **DGCR2*** | **DiGeorge syndrome critical region gene 2** | **22** | **-0.418** | **<0.000001** | **✔** |
| **ILMN_1657893** | **TXNRD2*** | **thioredoxin reductase 2** | **22** | **-0.402** | **<0.000001** | **✔** |
| **ILMN_1658950** | **LOC400890*** | **PREDICTED: hypothetical LOC400890** | **22** | **-0.4** | **<0.000001** |  |
| ILMN_1916256 |  | full-length cDNA clone CS0CAP005YH21 | 14 | -0.373 | 0.00004 |  |
| **ILMN_1789405** | **C22orf25*** | **chromosome 22 open reading frame 25** | **22** | **-0.368** | **0.00005** | **✔** |
| **ILMN_1728197** | **CLDN5*** | **claudin 5** | **22** | **-0.35** | **<0.000001** | **✔** |
| **ILMN_1813671** | **SLC25A1*** | **solute carrier family 25** | **22** | **-0.35** | **<0.000001** | **✔** |
| ILMN_1773650 | LRRN3 | leucine rich repeat neuronal 3 | 7 | -0.348 | 0.00242 | ✔ |
| ILMN_1737991 | LOC650298 | PREDICTED: similar to 40S ribosomal protein S26 |  | -0.343 | 0.00182 |  |
| ILMN_1715991 | SDPR | serum deprivation response | 2 | -0.341 | 0.00106 | ✔ |
| ILMN_2213136 | LEF1 | lymphoid enhancer-binding factor 1 | 4 | -0.328 | 0.00008 | ✔ |
| ILMN_2302757 | FCGBP | Fc fragment of IgG binding protein | 19 | -0.324 | 0.00039 | ✔ |
| **ILMN_1759595** | **C22orf29*** | **chromosome 22 open reading frame 29** | **22** | **-0.317** | **<0.000001** | **✔** |
| ILMN_1709237 | EPHX2 | epoxide hydrolase 2, cytoplasmic | 8 | -0.3 | 0.00001 | ✔ |
| **ILMN_1668748** | **MED15*** | **mediator complex subunit 15** | **22** | **-0.287** | **<0.000001** | **✔** |
| ILMN_1726460 | RPL14 | ribosomal protein L14 | 3 | -0.281 | 0.00206 | ✔ |
| ILMN_1679185 | LEF1 | lymphoid enhancer-binding factor 1 | 4 | -0.28 | 0.00179 | ✔ |
| ILMN_1657950 | RPS26P10 | PREDICTED: ribosomal protein S26 pseudogene 10 | 8 | -0.28 | 0.00104 |  |
| **ILMN_2383871** | **ZNF74*** | **zinc finger protein 74** | **22** | **-0.277** | **<0.000001** | **✔** |
| **ILMN_1719232** | **DGCR14*** | **DiGeorge syndrome critical region gene 14** | **22** | **-0.266** | **<0.000001** | **✔** |
| ILMN_1694432 | CRIP2 | cysteine-rich protein 2 | 14 | -0.265 | 0.00012 | ✔ |
| ILMN_2048591 | LRRN3 | leucine rich repeat neuronal 3 | 7 | -0.253 | 0.00223 | ✔ |
| ILMN_1655595 | SERPINE2 | serpin peptidase inhibitor, clade E, member 2 | 2 | -0.252 | 0.00004 |  |
| ILMN_2122103 | ETS1 | v-ets erythroblastosis virus E26 oncogene homolog 1 | 11 | -0.248 | 0.00067 | ✔ |
| ILMN_2327860 | MAL | mal, T-cell differentiation protein | 2 | -0.248 | 0.00103 | ✔ |
| ILMN_1661346 | LOC648210 | PREDICTED: similar to Heterogeneous nuclear ribonucleoprotein A1 |  | -0.244 | 0.00058 |  |
| **ILMN_1810941** | **COMT*** | **catechol-O-methyltransferase** | **22** | **-0.241** | **<0.000001** | **✔** |
| ILMN_1873034 |  | T cell receptor alpha locus, mRNA | 14 | -0.235 | 0.00038 |  |
| ILMN_2175131 | TMEM14C | transmembrane protein 14C | 6 | -0.234 | 0.00138 | ✔ |
| ILMN_1719986 | PIK3IP1 | phosphoinositide-3-kinase interacting protein 1 | 22 | -0.23 | 0.00081 | ✔ |
| ILMN_1724480 | AXIN2 | axin 2 | 17 | -0.229 | 0.00034 | ✔ |
| ILMN_1812191 | C12orf57 | chromosome 12 open reading frame 57 | 12 | -0.225 | 0.00453 | ✔ |
| **ILMN_1652486** | **THAP7*** | **THAP domain containing 7** | **22** | **-0.224** | **<0.000001** |  |
| **ILMN_1656184** | **PI4KAP1*** | **phosphatidylinositol 4-kinase, catalytic, alpha pseudogene 1** | **22** | **-0.221** | **<0.000001** |  |
| ILMN_1655126 | PI4KAP2 | phosphatidylinositol 4-kinase, catalytic, alpha polypeptide pseudogene 2 |  | -0.22 | 0.00002 |  |
| ILMN_1809040 | LDLRAP1 | low density lipoprotein receptor adaptor protein 1 | 1 | -0.215 | 0.00080 | ✔ |
| ILMN_1685538 | LOC158345 | PREDICTED: similar to ribosomal protein L4 | 9 | -0.215 | 0.00095 |  |
| **ILMN_1674768** | **LOC220686*** | **hypothetical protein LOC220686** | **22** | **-0.214** | **<0.000001** |  |
| ILMN_1849013 |  | primary neuroblastoma cDNA, clone:Nbla10111 | 5 | -0.211 | 0.00348 |  |
| ILMN_1810577 | RPS4X | ribosomal protein S4, X-linked | X | -0.207 | 0.00128 |  |
| ILMN_2342579 | IL7R | interleukin 7 receptor | 5 | -0.206 | 0.00354 | ✔ |
| ILMN_2387952 | FAM134B | family with sequence similarity 134, member B | 5 | -0.201 | 0.00061 | ✔ |
| **ILMN_1761044** | **GNB1L*** | **guanine nucleotide binding protein, beta polypeptide 1-like** | **22** | **-0.201** | **0.00001** |  |
| ILMN_1752591 | LEPROTL1 | leptin receptor overlapping transcript-like 1 | 8 | -0.201 | 0.00079 | ✔ |
| ILMN_1715947 | LOC648210 | PREDICTED: similar to Heterogeneous nuclear ribonucleoprotein A1 |  | -0.201 | 0.00235 |  |
| ILMN_1757186 | GIMAP1 | GTPase, IMAP family member 1 | 7 | -0.196 | 0.00068 | ✔ |
| **ILMN_1804884** | **C22orf39*** | **chromosome 22 open reading frame 39** | **22** | **-0.191** | **<0.000001** | **✔** |
| ILMN_2370091 | NGFRAP1 | nerve growth factor receptor | X | -0.191 | 0.00382 | ✔ |
| ILMN_1718128 | PABPC3 | poly(A) binding protein, cytoplasmic 3 | 13 | -0.191 | 0.00129 | ✔ |
| ILMN_1814526 | ADD3 | adducin 3 | 10 | -0.19 | 0.00336 | ✔ |
| ILMN_2125675 | LOC728643 | heterogeneous nuclear ribonucleoprotein A1 pseudogene | 10 | -0.189 | 0.00187 |  |
| ILMN_1767322 | EDAR | ectodysplasin A receptor | 2 | -0.188 | <0.000001 |  |
| ILMN_1665761 | BCL11B | B-cell CLL/lymphoma 11B | 14 | -0.185 | 0.00252 | ✔ |
| ILMN_1730572 | HNRPDL | heterogeneous nuclear ribo-nucleoprotein-like | 4 | -0.185 | 0.00001 | ✔ |
| ILMN_1726928 | TCEA3 | transcription elongation factor A (SII), 3 | 1 | -0.184 | 0.0026 | ✔ |
| ILMN_1657857 | TMEM14C | transmembrane protein 14C | 6 | -0.184 | 0.00028 | ✔ |
| ILMN_2405628 | TOP1MT | topoisomerase (DNA) I | 8 | -0.184 | 0.0013 | ✔ |
| ILMN_2166831 | RPS4X | ribosomal protein S4, X-linked | X | -0.18 | 0.00467 |  |
| ILMN_1785570 | SUSD3 | sushi domain containing 3 | 9 | -0.18 | 0.00092 | ✔ |
| **ILMN_1687266** | **TMEM191B*** | **PREDICTED: transmembrane protein 191B** | **22** | **-0.18** | **<0.000001** |  |
| ILMN_1785095 | ATP6V0E2 | ATPase, H+ transporting V0 subunit e2 | 7 | -0.179 | 0.00393 | ✔ |
| ILMN_1757872 | DKFZp761P0423 | PREDICTED: hypothetical protein DKFZp761P0423 | 8 | -0.177 | 0.00081 |  |
| ILMN_1787378 | ADD3 | adducin 3 | 10 | -0.176 | 0.00008 | ✔ |
| ILMN_2171384 | CXCL5 | chemokine ligand 5 | 4 | -0.176 | 0.00267 | ✔ |
| ILMN_1693452 | GAL3ST4 | galactose-3-O-sulfotransferase 4 | 7 | -0.176 | 0.00011 | ✔ |
| ILMN_1740493 | TRAF5 | TNF receptor-associated factor 5 | 1 | -0.176 | 0.00009 | ✔ |
| ILMN_1778143 | GRAP2 | GRB2-related adaptor protein 2 | 22 | -0.175 | 0.00001 | ✔ |
| ILMN_2110908 | MYC | v-myc myelocytomatosis viral oncogene homolog | 8 | -0.174 | 0.00204 | ✔ |
| ILMN_1736184 | GSTM3 | glutathione S-transferase M3 | 1 | -0.173 | 0.00024 | ✔ |
| ILMN_2401779 | FAM102A | family with sequence similarity 102, member A | 9 | -0.171 | 0.00132 | ✔ |
| ILMN_1659766 | BAG3 | BCL2-associated athanogene 3 | 10 | -0.169 | 0.00017 | ✔ |
| **ILMN_1754643** | **DGCR14*** | **DiGeorge syndrome critical region gene 14** | **22** | **-0.169** | **<0.000001** | **✔** |
| ILMN_1691949 | LOC728554 | PREDICTED: similar to THO complex 3 | 5 | -0.169 | 0.00327 |  |
| ILMN_1724718 | NCK2 | NCK adaptor protein 2 | 2 | -0.169 | 0.00005 | ✔ |
| ILMN_1712888 | HSPH1 | heat shock 105kDa/110kDa protein 1 | 13 | -0.168 | 0.00081 | ✔ |
| ILMN_1788607 | RPL23A | ribosomal protein L23a | 17 | -0.167 | 0.00215 |  |
| ILMN_1782057 | ATP8B2 | ATPase, class I, type 8B, member 2 | 1 | -0.166 | 0.00044 | ✔ |
| ILMN_1768958 | RASGRP1 | RAS guanyl releasing protein 1 (calcium and DAG-regulated) | 15 | -0.166 | 0.00315 | ✔ |
| ILMN_1782938 | SLC16A10 | solute carrier family 16, member 10 (aromatic amino acid transporter) | 6 | -0.166 | 0.00037 | ✔ |
| ILMN_1745112 | FAM102A | family with sequence similarity 102, member A | 9 | -0.165 | 0.00163 | ✔ |
| **ILMN_1682894** | **TMEM191B*** | **PREDICTED: transmembrane protein 191B** | **22** | **-0.165** | **0.00001** |  |
| ILMN_1788701 | PSIP1 | PC4 and SFRS1 interacting protein 1 | 9 | -0.164 | 0.00144 | ✔ |
| ILMN_2104877 | CMPK1 | cytidine monophosphate (UMP-CMP) kinase 1, cytosolic | 1 | -0.162 | 0.00287 | ✔ |
| ILMN_1671568 | ECHDC2 | enoyl Coenzyme A hydratase domain containing 2 | 1 | -0.162 | 0.00344 | ✔ |
| ILMN_2337974 | PKIA | protein kinase (cAMP-dependent, catalytic) inhibitor alpha | 8 | -0.16 | 0.00288 | ✔ |
| ILMN_2051684 | LOC401152 | HCV F-transactivated protein 1 | 4 | -0.158 | 0.00015 |  |
| ILMN_1801109 | NARG1L | NMDA receptor regulated 1-like | 13 | -0.158 | 0.00011 |  |
| ILMN_1863099 |  | DB113199 THYMU2 cDNA clone THYMU2017679 5 | 14 | -0.157 | 0.00021 |  |
| **ILMN_1694223** | **DGCR8*** | **DiGeorge syndrome critical region gene 8** | **22** | **-0.153** | **0.00025** | **✔** |
| ILMN_1664176 | FBLN5 | fibulin 5 | 14 | -0.153 | 0.00225 | ✔ |
| ILMN_1682572 | KIAA0528 | KIAA0528 | 12 | -0.152 | 0.00141 | ✔ |
| ILMN_1702301 | DOCK10 | dedicator of cytokinesis 10 | 2 | -0.15 | 0.00325 | ✔ |
| ILMN_1659845 | KIAA0355 | KIAA0355 | 19 | -0.149 | 0.0001 | ✔ |
| ILMN_1703279 | CXorf57 | chromosome X open reading frame 57 | X | -0.148 | 0.00132 | ✔ |
| ILMN_1694589 | PAQR8 | progestin and adipoQ receptor family member VIII | 6 | -0.148 | 0.00304 | ✔ |
| ILMN_1712719 | MAP7 | microtubule-associated protein 7 | 6 | -0.146 | 0.00146 | ✔ |
| ILMN_2055156 | PAG1 | phosphoprotein associated with glycosphingolipid microdomains 1 | 8 | -0.146 | 0.00334 | ✔ |
| ILMN_1677138 | POLR2J3 | polymerase (RNA) II (DNA directed) polypeptide J3 | 7 | -0.144 | 0.00468 |  |
| ILMN_2094106 | HSD17B12 | hydroxysteroid (17-beta) dehydrogenase 12 | 11 | -0.143 | 0.00006 | ✔ |
| ILMN_1890134 |  | T cell receptor alpha variable 20 | 14 | -0.142 | 0.00001 |  |
| ILMN_1711514 | COCH | coagulation factor C homolog, cochlin | 14 | -0.14 | 0.00162 | ✔ |
| ILMN_1680738 | C5orf13 | chromosome 5 open reading frame 13 | 5 | -0.139 | 0.00037 | ✔ |
| ILMN_1731610 | ABLIM1 | actin binding LIM protein 1 | 10 | -0.138 | 0.00091 | ✔ |
| ILMN_2311041 | MRPL52 | mitochondrial ribosomal protein L52 | 14 | -0.138 | 0.00199 |  |
| ILMN_1776327 | LOC727820 | PREDICTED: hypothetical protein LOC727820 | 1 | -0.137 | 0.00171 |  |
| ILMN_2362581 | FNDC3A | fibronectin type III domain containing 3A | 13 | -0.134 | 0.00099 | ✔ |
| ILMN_1761131 | PECI | peroxisomal D3,D2-enoyl-CoA isomerase | 6 | -0.133 | 0.00424 |  |
| ILMN_1688971 | NOL11 | nucleolar protein 11 | 17 | -0.131 | 0.00237 | ✔ |
| ILMN_2176768 | SEPHS1 | selenophosphate synthetase 1 | 10 | -0.131 | 0.00142 | ✔ |
| ILMN_1693341 | SNRPN | small nuclear ribonucleoprotein polypeptide N | 15 | -0.131 | 0.0013 | ✔ |
| ILMN_1678004 | TMEM41B | transmembrane protein 41B | 11 | -0.131 | 0.00431 |  |
| ILMN_1677785 | ZNF559 | zinc finger protein 559 | 19 | -0.131 | 0.00047 | ✔ |
| ILMN_1669831 | C6orf192 | chromosome 6 open reading frame 192 | 6 | -0.129 | 0.00189 | ✔ |
| **ILMN_2186108** | **DGCR6*** | **DiGeorge syndrome critical region gene 6** | **22** | **-0.129** | **0.00012** |  |
| ILMN_1776000 | EID3 | EP300 interacting inhibitor of differentiation 3 | 12 | -0.129 | 0.00014 | ✔ |
| ILMN_2395474 | REV1 | REV1 homolog | 2 | -0.126 | 0.00032 | ✔ |
| ILMN_1694491 | CCNG1 | cyclin G1 | 5 | -0.124 | 0.00461 | ✔ |
| ILMN_1780141 | TMEM66 | transmembrane protein 66 | 8 | -0.123 | 0.00391 | ✔ |
| ILMN_1728230 | ZNF30 | zinc finger protein 30 | 19 | -0.122 | 0.00139 |  |
| ILMN_1780806 | ANKRD36B | ankyrin repeat domain | 2 | -0.121 | 0.00362 | ✔ |
| ILMN_1843100 |  | T-cell receptor alpha chain-like protein mRNA | 14 | -0.121 | 0.00172 |  |
| ILMN_1755405 | FRAG1 | FGF receptor activating protein 1 | 11 | -0.12 | 1.00E-04 |  |
| ILMN_2399016 | MMP28 | matrix metallopeptidase 28 | 17 | -0.12 | 0.00177 | ✔ |
| ILMN_1771120 | TMEM45B | transmembrane protein 45B | 11 | -0.12 | 0.00102 | ✔ |
| ILMN_2154053 | FVT1 | follicular lymphoma variant translocation 1 | 18 | -0.119 | 0.00472 |  |
| ILMN_1694778 | LOC646723 | PREDICTED: similar to Keratin, type I cytoskeletal 18 | 5 | -0.119 | 0.00002 |  |
| **ILMN_1750203** | **THAP7*** | **THAP domain containing 7** | **22** | **-0.119** | **0.00003** |  |
| ILMN_2194828 | C16orf53 | chromosome 16 open reading frame 53 | 16 | -0.118 | 0.00057 | ✔ |
| ILMN_1856315 |  | cDNA FLJ34428 fis, clone HLUNG2000761 |  | -0.118 | 0.00382 |  |
| ILMN_1767135 | SOS1 | son of sevenless homolog 1 | 2 | -0.115 | 0.00253 | ✔ |
| ILMN_1758474 | PRKRA | protein kinase, interferon-inducible double stranded RNA dependent activator | 2 | -0.113 | 0.00278 | ✔ |
| ILMN_2403852 | CYTH1 | cytohesin 1 | 17 | -0.112 | 0.00176 | ✔ |
| ILMN_2118229 | NAPEPLD | N-acyl phosphatidylethanolamine phospholipase D | 7 | -0.112 | 0.00137 | ✔ |
| ILMN_1683740 | TOP1MT | PREDICTED: topoisomerase (DNA) I |  | -0.112 | 0.00047 | ✔ |
| ILMN_2324561 | SLC7A6 | solute carrier family 7 (cationic amino acid transporter, y+ system), member 6 | 16 | -0.111 | 0.00297 | ✔ |
| ILMN_1800311 | HSF2 | heat shock transcription factor 2 | 6 | -0.11 | 0.00212 | ✔ |
| ILMN_1785170 | ARMCX2 | armadillo repeat containing, X-linked 2 | X | -0.109 | 0.00372 | ✔ |
| **ILMN_1655941** | **DKFZp434-N035*** | **hypothetical protein DKFZp434N035** | **22** | **-0.109** | **0.00004** |  |
| ILMN_1694514 | ZDHHC11 | zinc finger, DHHC-type containing 11 | 5 | -0.109 | 0.0015 | ✔ |
| ILMN_1741957 | RABEPK | Rab9 effector protein with kelch motifs | 9 | -0.108 | 0.00052 |  |
| ILMN_2358457 | ATF4 | activating transcription factor 4 (tax-responsive enhancer element B67) | 22 | -0.107 | 0.00036 | ✔ |
| ILMN_1669366 | IFT88 | intraflagellar transport 88 homolog | 13 | -0.107 | 0.00045 | ✔ |
| ILMN_1746784 | SLAIN1 | SLAIN motif family, member 1 | 13 | -0.107 | 0.00295 | ✔ |
| ILMN_1665066 | C4orf14 | chromosome 4 open reading frame 14 | 4 | -0.106 | 0.00151 | ✔ |
| ILMN_1822333 |  | 602507809F1 NIH_MGC_79 cDNA clone IMAGE:4604835 5 | 2 | -0.106 | 0.00491 |  |
| ILMN_2198239 | HGD | homogentisate 1,2-dioxygenase | 3 | -0.105 | 0.004 |  |
| ILMN_1741477 | SMAD4 | SMAD family member 4 | 18 | -0.105 | 0.00392 | ✔ |
| ILMN_1676893 | ADCY3 | adenylate cyclase 3 | 2 | -0.102 | 0.00215 | ✔ |
| ILMN_1762002 | CSTF3 | cleavage stimulation factor, 3' pre-RNA, subunit 3, 77kDa | 11 | -0.102 | 0.00472 | ✔ |
| ILMN_1671217 | LOC440733 | PREDICTED: similar to 40S ribosomal protein S15 | 1 | -0.102 | 0.001 |  |
| ILMN_1721316 | TNFRSF10A | tumor necrosis factor receptor superfamily, member 10a | 8 | -0.102 | 0.00369 |  |
| ILMN_2293374 | TOP1MT | topoisomerase (DNA)I | 8 | -0.102 | 0.00387 | ✔ |
| ILMN_1655137 | ZCCHC11 | zinc finger, CCHC domain containing 11 | 1 | -0.102 | 0.00415 | ✔ |
| ILMN_1784269 | AASDH | aminoadipate-semialdehyde dehydrogenase | 4 | -0.101 | 0.00287 | ✔ |
| ILMN_1704452 | BCL9 | B-cell CLL/lymphoma 9 | 1 | -0.101 | 0.00341 | ✔ |
| ILMN_1722809 | NRCAM | neuronal cell adhesion molecule | 7 | -0.101 | 0.00061 | ✔ |
| ILMN_2246510 | TSC1 | tuberous sclerosis 1 | 9 | -0.101 | 0.00099 | ✔ |
| ILMN_1737360 | TSPYL3 | PREDICTED: TSPY-like 3 |  | -0.1 | 0.00014 |  |
| ILMN_1749478 | TCEAL3 | transcription elongation factor A (SII)-like 3 | X | -0.099 | 0.00402 |  |
| ILMN_1656134 | CNOT7 | CCR4-NOT transcription complex, subunit 7 | 8 | -0.098 | 0.00104 | ✔ |
| ILMN_1698019 | LGMN | legumain | 14 | -0.098 | 0.00284 | ✔ |
| ILMN_1688299 | RCAN3 | RCAN family member 3 | 1 | -0.098 | 0.00201 | ✔ |
| ILMN_1715407 | LOC644404 | PREDICTED: hypothetical protein LOC644404 | 1 | -0.097 | 0.00043 |  |
| ILMN_1803180 | PRDX6 | peroxiredoxin 6 | 1 | -0.097 | 0.0048 | ✔ |
| ILMN_1670801 | MTR | 5-methyltetrahydrofolate-homocysteine methyltransferase | 1 | -0.096 | 0.0033 | ✔ |
| ILMN_1701514 | TRAF3IP2 | TRAF3 interacting protein 2 | 6 | -0.096 | 0.00405 | ✔ |
| ILMN_1898071 |  | Human mRNA for T-cell receptor alpha-chain J segment, partial cds, clone WADM36A | 14 | -0.095 | 0.00030 |  |
| ILMN_2147993 | ZNF23 | zinc finger protein 23 | 16 | -0.093 | 0.00231 | ✔ |
| ILMN_1803429 | CD44 | CD44 molecule | 11 | -0.091 | 0.00404 | ✔ |
| ILMN_1681972 | TMEM69 | transmembrane protein 69 | 1 | -0.091 | 0.00327 | ✔ |
| ILMN_1746175 | TNFSF4 | tumor necrosis factor (ligand) superfamily, member 4 | 1 | -0.09 | 0.00294 | ✔ |
| **ILMN_1665737** | **UFD1L*** | **ubiquitin fusion degradation 1 like** | **22** | **-0.09** | **0.00049** | **✔** |
| ILMN_1775085 | ZNF232 | zinc finger protein 232 | 17 | -0.09 | 0.00112 | ✔ |
| ILMN_1858271 |  | EST7714 human nasopharynx cDNA | 14 | -0.088 | 0.00453 |  |
| ILMN_1679582 | LOC727735 | PREDICTED: similar to TBC1 domain family member 3 | 17 | -0.086 | 0.00197 |  |
| ILMN_1804737 | RAVER2 | ribonucleoprotein, PTB-binding 2 | 1 | -0.086 | 0.00328 | ✔ |
| ILMN_1698470 | SYAP1 | synapse associated protein 1, SAP47 homolog | X | -0.086 | 0.00435 | ✔ |
| ILMN_1902146 |  | T-cell receptor alpha precursor | 14 | -0.085 | 0.00385 |  |
| ILMN_1658083 | ABT1 | activator of basal transcription 1 | 6 | -0.084 | 0.00196 | ✔ |
| ILMN_1829989 |  | cDNA: FLJ23004 fis, clone LNG00391 | 10 | -0.084 | 0.00181 |  |
| ILMN_1724699 | ACAD8 | acyl-Coenzyme A dehydrogenase family, member 8 | 11 | -0.083 | 0.00217 | ✔ |
| ILMN_2357193 | DDX59 | DEAD (Asp-Glu-Ala-Asp) box polypeptide 59 | 1 | -0.083 | 0.00374 | ✔ |
| ILMN_1718984 | FCGBP | PREDICTED: Fc fragment of IgG binding protein |  | -0.083 | 0.00489 | ✔ |
| **ILMN_1706200** | **MED15*** | **mediator complex subunit 15** | **22** | **-0.083** | **0.00195** | **✔** |
| ILMN_1669876 | CUX2 | cut-like homeobox 2 | 12 | -0.082 | 0.00055 |  |
| ILMN_2055609 | GPR174 | G protein-coupled receptor 174 | X | -0.081 | 0.00113 |  |
| ILMN_1714741 | LOC346887 | PREDICTED: similar to solute carrier family 16, member 14 |  | -0.081 | 0.0039 |  |
| ILMN_2079508 | ZNF204 | zinc finger protein 204 pseudogene | 6 | -0.081 | 0.00089 | ✔ |
| **ILMN_1694584** | **CLTCL1*** | **clathrin, heavy chain-like 1** | **22** | **-0.08** | **0.00187** | **✔** |
| **ILMN_2367458** | **UFD1L*** | **ubiquitin fusion degradation 1 like** | **22** | **-0.08** | **0.00077** | **✔** |
| ILMN_2404917 | AFAP1L2 | actin filament associated protein 1-like 2 | 10 | -0.079 | 0.00183 | ✔ |
| ILMN_1763999 | LOC441282 | PREDICTED: similar to aldo-keto reductase family 1, member B10, transcript variant 7 |  | -0.078 | 0.00043 |  |
| **ILMN_2310075** | **TRMT2A*** | **TRM2 tRNA methyltransferase 2 homolog A** | **22** | **-0.078** | **0.00055** | **✔** |
| ILMN_1739624 | UGT2B15 | UDP glucuronosyltransferase 2 family, polypeptide B15 | 4 | -0.076 | 0.00066 | ✔ |
| ILMN_1708059 | USP13 | ubiquitin specific peptidase 13 (isopeptidase T-3) | 3 | -0.076 | 0.00177 | ✔ |
| ILMN_1810185 | LOC150356 | PREDICTED: hypothetical protein BC012882 | 22 | -0.075 | 0.00078 |  |
| ILMN_1749586 | LOC642914 | PREDICTED: similar to zinc finger protein 135 | 8 | -0.075 | 0.00374 |  |
| ILMN_1723333 | SCGB2A2 | secretoglobin, family 2A, member 2 | 11 | -0.075 | 0.00197 |  |
| ILMN_1698739 | UNC45B | unc-45 homolog B | 17 | -0.075 | 0.00042 | ✔ |
| ILMN_1662935 | C1QTNF7 | C1q and tumor necrosis factor related protein 7 | 4 | -0.074 | 0.0006 | ✔ |
| ILMN_1766675 | CDH6 | cadherin 6, type 2, K-cadherin | 5 | -0.073 | 0.00205 | ✔ |
| ILMN_2082865 | PLLP | plasma membrane proteolipid | 16 | -0.072 | 0.0045 | ✔ |
| ILMN_2263086 | NTM | neurotrimin | 11 | -0.071 | 0.00357 | ✔ |
| ILMN_2159311 | RFPL2 | ret finger protein-like 2 | 22 | -0.07 | 0.00285 |  |
| ILMN_1782881 | DLEU7 | deleted in lymphocytic leukemia, 7 | 13 | -0.069 | 0.00291 | ✔ |
| ILMN_2332964 | LGMN | legumain | 14 | -0.069 | 0.00457 | ✔ |
| ILMN_2098726 | LOC440350 | similar to nuclear pore complex interacting protein | 16 | -0.069 | 0.00399 |  |
| ILMN_1662294 | LOC650114 | PREDICTED: hypothetical protein LOC650113 |  | -0.069 | 0.00453 |  |
| ILMN_1785156 | CASRL1 | PREDICTED: calcium-sensing receptor like 1 | 3 | -0.068 | 0.00467 |  |
| ILMN_2287941 | CYLD | cylindromatosis | 16 | -0.068 | 0.00371 | ✔ |
| ILMN_1786080 | HTR4 | 5-hydroxytryptamine (serotonin) receptor 4 | 5 | -0.068 | 0.00472 | ✔ |
| ILMN_1807126 | LOC392979 | PREDICTED: similar to ribosomal protein L18 |  | -0.068 | 0.00338 |  |
| ILMN_1798940 | LOC644131 | PREDICTED: similar to chaperonin containing TCP1, subunit 8 | 1 | -0.068 | 0.00362 |  |
| ILMN_2329171 | SPDYE1 | speedy homolog E1 | 7 | -0.068 | 0.00113 |  |
| ILMN_1824016 |  | 602349056F1 NIH_MGC_90 cDNA clone IMAGE:4444260 5 | X | -0.068 | 0.0013 |  |
| ILMN_1750288 | FLJ39739 | FLJ39739 protein | 1 | -0.066 | 0.00418 |  |
| ILMN_1665630 | LOC643872 | PREDICTED: similar to Nonhistone chromosomal protein HMG-17 | 14 | -0.066 | 0.00424 |  |
| ILMN_1668990 | LOC646249 | PREDICTED: similar to Protein FAM113A |  | -0.066 | 0.00188 |  |
| ILMN_2342695 | PDGFA | platelet-derived growth factor alpha polypeptide | 7 | -0.065 | 0.00228 |  |
| ILMN_2105797 | SLC6A3 | solute carrier family 6 member 3 | 5 | -0.065 | 0.00246 | ✔ |
| ILMN_1902112 |  | ai24c04.s1 Soares_testis_NHT cDNA clone 1343718 3 | 10 | -0.063 | 0.00251 |  |
| ILMN_2296011 | BRWD1 | bromodomain and WD repeat domain containing 1 | 21 | -0.061 | 0.00328 | ✔ |
| ILMN_1663155 | ZNF238 | zinc finger protein 238 | 1 | -0.06 | 0.00257 | ✔ |
| ILMN_1680659 | C11orf63 | chromosome 11 open reading frame 63 | 11 | -0.058 | 0.00443 | ✔ |
| ILMN_1744815 | PVRL1 | poliovirus receptor-related 1 | 11 | -0.057 | 0.0046 | ✔ |
| ILMN_2380779 | DLGAP1 | discs, large homolog-associated protein 1 | 18 | 0.051 | 0.00378 | ✔ |
| ILMN_2093203 | HTR3D | 5-hydroxytryptamine (serotonin) receptor 3 family member D | 3 | 0.053 | 0.00303 | ✔ |
| ILMN_1667796 | HBA2 | hemoglobin, alpha 2 | 16 | 0.055 | 0.00357 |  |
| ILMN_2126461 | OR4K2 | olfactory receptor, family 4, subfamily K, member 2 | 14 | 0.055 | 0.0038 |  |
| ILMN_1775549 | PRSS27 | protease, serine 27 | 16 | 0.055 | 0.00291 | ✔ |
| ILMN_1705372 | THEG | Theg homolog | 19 | 0.055 | 0.00185 | ✔ |
| ILMN_1778657 | LOC730092 | RRN3 RNA polymerase I transcription factor homolog (S. cerevisiae) pseudogene |  | 0.056 | 0.00499 |  |
| ILMN_1680941 | ACTR3B | PREDICTED: ARP3 actin-related protein 3 homolog B |  | 0.057 | 0.00311 | ✔ |
| ILMN_1735704 | NMBR | neuromedin B receptor | 6 | 0.057 | 0.00282 | ✔ |
| ILMN_1661971 | ARHGAP25 | Rho GTPase activating protein 25 | 2 | 0.058 | 0.0019 | ✔ |
| ILMN_1651763 | TFDP3 | transcription factor Dp family, member 3 | X | 0.058 | 0.00409 |  |
| ILMN_1669609 | LOC644767 | PREDICTED: hypothetical protein LOC644767 | 17 | 0.06 | 0.00369 |  |
| ILMN_1814311 | LOC653559 | PREDICTED: similar to Rho-associated protein kinase 1 | 18 | 0.06 | 0.00305 |  |
| ILMN_1695005 | C1orf62 | chromosome 1 open reading frame 62 | 1 | 0.061 | 0.00342 |  |
| ILMN_1796475 | GPR6 | G protein-coupled receptor 6 | 6 | 0.061 | 0.00242 | ✔ |
| ILMN_1746232 | KITLG | KIT ligand | 12 | 0.061 | 0.00415 | ✔ |
| ILMN_1891380 |  | RC3-HT0883-020800-021-e12 HT0883 | 10 | 0.061 | 0.00257 |  |
| ILMN_1709650 | LOC652815 | PREDICTED: similar to FK506-binding protein 9 precursor |  | 0.062 | 0.00387 |  |
| ILMN_1748141 | AMOTL1 | angiomotin like 1 | 11 | 0.064 | 0.00236 | ✔ |
| ILMN_1680367 | C10orf90 | chromosome 10 open reading frame 90 | 10 | 0.064 | 0.0024 | ✔ |
| ILMN_1654899 | CT47A6 | cancer/testis antigen family 47, member A6 | X | 0.065 | 0.00131 |  |
| ILMN_1808650 | CYP39A1 | cytochrome P450, family 39, subfamily A, polypeptide 1 | 6 | 0.065 | 0.00471 | ✔ |
| ILMN_1817479 |  | cDNA FLJ26380 fis, clone HRT06687 | 7 | 0.065 | 0.00499 |  |
| ILMN_2399694 | NOMO2 | NODAL modulator 2 | 16 | 0.066 | 0.00303 |  |
| ILMN_2376553 | SLC23A1 | solute carrier family 23 (nucleobase transporters), member 1 | 5 | 0.066 | 0.00236 | ✔ |
| ILMN_2262644 | KCNIP3 | Kv channel interacting protein 3, calsenilin | 2 | 0.067 | 0.00167 | ✔ |
| ILMN_1699723 | RARB | retinoic acid receptor, beta | 3 | 0.068 | 0.00365 | ✔ |
| ILMN_1665167 | RORA | RAR-related orphan receptor A | 15 | 0.068 | 0.00144 | ✔ |
| ILMN_1714218 | LOC646631 | PREDICTED: hypothetical protein LOC646631 | 14 | 0.069 | 0.00123 |  |
| ILMN_1658111 | MGC34800 | PREDICTED: hypothetical protein MGC34800 |  | 0.069 | 0.00041 |  |
| ILMN_1799020 | MUC12 | PREDICTED: mucin 12, cell surface associated |  | 0.07 | 0.00145 |  |
| ILMN_2068122 | TMEM65 | transmembrane protein 65 | 8 | 0.07 | 0.00486 | ✔ |
| ILMN_1654473 | ZP1 | zona pellucida glycoprotein 1 | 11 | 0.07 | 0.00101 | ✔ |
| ILMN_1898268 |  | AF150386 Human mRNA from cd34+ stem cells cDNA clone CBMAPB12 | 13 | 0.07 | 0.00209 |  |
| ILMN_1783383 | DCDC1 | doublecortin domain containing 1 | 11 | 0.076 | 0.00308 | ✔ |
| ILMN_1733116 | UBXD7 | PREDICTED: UBX domain containing 7, transcript variant 4 |  | 0.076 | 0.00269 |  |
| ILMN_1778408 | FLJ35424 | hypothetical protein FLJ35424 | 4 | 0.077 | 0.00313 |  |
| ILMN_1774211 | FLJ45032 | similar to F40B5.2b | 3 | 0.077 | 0.00254 |  |
| ILMN_1875269 |  | tz62a11.x1 NCI_CGAP_Ov35 cDNA clone IMAGE:2293148 3 similar to contains L1.t3 L1 repetitive element |  | 0.077 | 0.00416 |  |
| ILMN_1706413 | C1orf66 | chromosome 1 open reading frame 66 | 1 | 0.078 | 0.00473 |  |
| ILMN_2047354 | C1orf97 | chromosome 1 open reading frame 97 |  | 0.078 | 0.00046 | ✔ |
| ILMN_1763561 | OSBPL6 | oxysterol binding protein-like 6 | 2 | 0.078 | 0.00455 | ✔ |
| ILMN_1821531 |  | AV681673 GKB cDNA clone GKBABD06 5 | 12 | 0.078 | 0.00162 |  |
| ILMN_2412336 | AKR1C2 | aldo-keto reductase family 1, member C2 | 10 | 0.079 | 0.00423 | ✔ |
| ILMN_1812688 | C2orf18 | chromosome 2 open reading frame 18 | 2 | 0.079 | 0.00434 | ✔ |
| ILMN_1772239 | LOC400406 | PREDICTED: similar to ADAM metallopeptidase with thrombospondin type 1 motif, 7 preproprotein | 15 | 0.079 | 0.00081 |  |
| ILMN_1715309 | LOC650885 | PREDICTED: hypothetical protein LOC650885 |  | 0.08 | 0.00225 |  |
| ILMN_1705141 | CACYBP | calcyclin binding protein | 1 | 0.081 | 0.0027 | ✔ |
| ILMN_1786381 | AQP6 | aquaporin 6, kidney specific | 12 | 0.083 | 0.00021 |  |
| ILMN_1704795 | MARK3 | MAP/microtubule affinity-regulating kinase 3 | 14 | 0.085 | 0.00201 | ✔ |
| ILMN_1815666 | ATP2A2 | ATPase, Ca++ transporting, cardiac muscle, slow twitch 2 | 12 | 0.086 | 0.0039 | ✔ |
| ILMN_1722330 | GK2 | glycerol kinase 2 | 4 | 0.086 | 0.00417 | ✔ |
| ILMN_2348243 | STRADA | STE20-related kinase adaptor alpha | 17 | 0.086 | 0.00485 | ✔ |
| ILMN_1690822 | VAPA | VAMP (vesicle-associated membrane protein)-associated protein A, 33kDa | 18 | 0.086 | 0.00382 | ✔ |
| ILMN_1775171 | WIPF2 | WAS/WASL interacting protein family, member 2 | 17 | 0.089 | 0.00372 |  |
| ILMN_1739450 | NFE2L1 | nuclear factor (erythroid-derived 2)-like 1 | 17 | 0.091 | 0.00162 | ✔ |
| ILMN_1892895 |  | UI-E-EJ1-ajq-h-17-0-UI.s1 UI-E-EJ1 cDNA clone UI-E-EJ1-ajq-h-17-0-UI 3 | 5 | 0.091 | 0.0041 |  |
| ILMN_1716869 | GPM6A | glycoprotein M6A | 4 | 0.092 | 0.00147 | ✔ |
| ILMN_1775742 | RNF128 | ring finger protein 128 | X | 0.093 | 0.00326 | ✔ |
| ILMN_1690682 | B3GALT4 | UDP-Gal:betaGlcNAc beta 1,3-galactosyltransferase, polypeptide 4 | 6 | 0.094 | 0.00421 | ✔ |
| ILMN_1712673 | SASH1 | SAM and SH3 domain containing 1 | 6 | 0.094 | 0.00395 | ✔ |
| ILMN_1674874 | MFSD10 | major facilitator superfamily domain containing 10 | 4 | 0.095 | 0.00186 | ✔ |
| ILMN_1705783 | NXF1 | nuclear RNA export factor 1 | 11 | 0.095 | 0.00447 | ✔ |
| ILMN_2378257 | SDF4 | stromal cell derived factor 4 | 1 | 0.098 | 0.00492 | ✔ |
| ILMN_2278265 | PAOX | polyamine oxidase (exo-N4-amino) | 10 | 0.1 | 0.00273 | ✔ |
| ILMN_1812403 | BCAP31 | B-cell receptor-associated protein 31 | X | 0.101 | 0.00253 | ✔ |
| ILMN_1796244 | CD2BP2 | CD2 (cytoplasmic tail) binding protein 2 | 16 | 0.101 | 0.00051 | ✔ |
| ILMN_1748908 | PROSC | proline synthetase co-transcribed homolog | 8 | 0.101 | 0.00239 | ✔ |
| ILMN_1793832 | GRB14 | growth factor receptor-bound protein 14 | 2 | 0.102 | 0.00369 | ✔ |
| ILMN_1800276 | RCN1 | reticulocalbin 1, EF-hand calcium binding domain | 11 | 0.104 | 0.00144 |  |
| ILMN_1732176 | AGPAT2 | 1-acylglycerol-3-phosphate O-acyltransferase 2 | 9 | 0.105 | 0.00153 | ✔ |
| ILMN_1771651 | MON1B | MON1 homolog B | 16 | 0.105 | 0.00475 | ✔ |
| ILMN_1773850 | FXC1 | fracture callus 1 homolog | 11 | 0.108 | 0.002 | ✔ |
| ILMN_1671509 | CCL3 | chemokine (C-C motif) ligand 3 | 17 | 0.109 | 0.00357 |  |
| ILMN_2203891 | SMAD7 | SMAD family member 7 | 18 | 0.11 | 0.00056 | ✔ |
| ILMN_1703894 | BOLA2 | bolA homolog 2 | 16 | 0.113 | 0.00269 |  |
| ILMN_1730620 | ARRB1 | arrestin, beta 1 | 11 | 0.114 | 0.00465 | ✔ |
| ILMN_1782015 | FCRLB | Fc receptor-like B | 1 | 0.116 | 0.00024 |  |
| ILMN_1751886 | REC8 | REC8 homolog | 14 | 0.116 | 0.0041 | ✔ |
| ILMN_1695978 | LINGO2 | leucine rich repeat and Ig domain containing 2 | 9 | 0.119 | 0.00018 | ✔ |
| ILMN_1656315 | DMD | dystrophin (muscular dystrophy, Duchenne and Becker types) | X | 0.12 | 0.00027 | ✔ |
| ILMN_1671237 | GNGT2 | guanine nucleotide binding protein (G protein), gamma transducing activity polypeptide 2 | 17 | 0.122 | 0.00042 |  |
| ILMN_1691334 | TXNDC3 | thioredoxin domain containing 3 | 7 | 0.123 | 0.00434 |  |
| ILMN_2119535 | RIPK1 | receptor (TNFRSF)-interacting serine-threonine kinase 1 | 6 | 0.124 | 0.00191 | ✔ |
| ILMN_1783956 | ATP8B4 | ATPase, class I, type 8B, member 4 | 15 | 0.127 | 0.0044 | ✔ |
| ILMN_2305407 | ZBTB16 | zinc finger and BTB domain containing 16 | 11 | 0.127 | 0.00291 | ✔ |
| ILMN_2398926 | C17orf58 | chromosome 17 open reading frame 58 | 17 | 0.129 | 0.00008 | ✔ |
| ILMN_1723743 | ROM1 | retinal outer segment membrane protein 1 | 11 | 0.129 | 0.00365 | ✔ |
| ILMN_1790973 | CDS2 | CDP-diacylglycerol synthase (phosphatidate cytidylyltransferase) 2 | 20 | 0.13 | 0.00214 | ✔ |
| ILMN_1729287 | NMUR1 | neuromedin U receptor 1 | 2 | 0.13 | 0.00201 | ✔ |
| ILMN_1805979 | GAB3 | GRB2-associated binding protein 3 | X | 0.131 | 0.0002 |  |
| ILMN_1824666 |  | mRNA; cDNA DKFZp667A182 |  | 0.137 | 0.00054 |  |
| ILMN_1672662 | SLC20A1 | solute carrier family 20 (phosphate transporter), member 1 | 2 | 0.139 | 0.00021 | ✔ |
| ILMN_2052208 | GADD45A | growth arrest and DNA-damage-inducible, alpha | 1 | 0.14 | 0.00416 | ✔ |
| ILMN_1714393 | RAB24 | RAB24, member RAS oncogene family | 5 | 0.144 | 0.00048 | ✔ |
| ILMN_1687757 | AKR1C4 | aldo-keto reductase family 1, member C4 | 10 | 0.147 | 0.0007 |  |
| ILMN_2043816 | ARPC5L | actin related protein 2/3 complex, subunit 5-like | 9 | 0.147 | 0.00117 | ✔ |
| ILMN_2086077 | JUNB | jun B proto-oncogene | 19 | 0.148 | 0.00254 | ✔ |
| ILMN_1688775 | METRNL | PREDICTED: meteorin, glial cell differentiation regulator-like |  | 0.151 | 0.00121 | ✔ |
| ILMN_1725534 | ACTN4 | actinin, alpha 4 | 19 | 0.153 | 0.00416 | ✔ |
| ILMN_1759326 | P2RX7 | purinergic receptor P2X, ligand-gated ion channel, 7 | 12 | 0.153 | 0.00046 | ✔ |
| ILMN_1769779 | PTP4A3 | protein tyrosine phosphatase type IVA, member 3 | 8 | 0.153 | 0.0043 | ✔ |
| ILMN_2053415 | LDLR | low density lipoprotein receptor | 19 | 0.155 | 0.00169 | ✔ |
| ILMN_1780861 | LOC653506 | PREDICTED: similar to meteorin, glial cell differentiation regulator-like | 17 | 0.156 | 0.00169 |  |
| ILMN_1740466 | FAM46A | family with sequence similarity 46, member A | 6 | 0.16 | 0.00046 | ✔ |
| ILMN_2390310 | C17orf91 | chromosome 17 open reading frame 91 | 17 | 0.162 | 0.00078 | ✔ |
| ILMN_1719975 | HOXC4 | homeobox C4 | 12 | 0.162 | 0.00002 |  |
| ILMN_1792473 | AIF1 | allograft inflammatory factor 1 | 6 | 0.163 | 0.00407 | ✔ |
| ILMN_1811779 | MGC24103 | PREDICTED: hypothetical protein MGC24103 | 9 | 0.163 | 0.00024 |  |
| ILMN_1730710 | ADORA3 | adenosine A3 receptor | 1 | 0.165 | 0.00364 | ✔ |
| ILMN_1749591 | ITGAL | integrin, alpha L (antigen CD11A (p180), lymphocyte function-associated antigen 1; alpha polypeptide) | 16 | 0.165 | 0.00063 |  |
| ILMN_1781824 | FASLG | Fas ligand | 1 | 0.166 | 0.00247 |  |
| ILMN_1693552 | CD300A | CD300a molecule | 17 | 0.168 | 0.00043 | ✔ |
| ILMN_1776125 | TRK1 | tRNA lysine 1 |  | 0.172 | 0.0026 |  |
| ILMN_1907042 |  | cDNA FLJ41270 fis, clone BRAMY2036387 | 1 | 0.174 | 0.00002 |  |
| ILMN_2329114 | COLQ | collagen-like tail subunit (single strand of homotrimer) of asymmetric acetylcholinesterase |  | 0.175 | 0.00024 | ✔ |
| ILMN_1666902 | GPR114 | G protein-coupled receptor 114 | 16 | 0.176 | 0.00287 | ✔ |
| ILMN_1811387 | TFF3 | trefoil factor 3 | 21 | 0.176 | 0.0027 | ✔ |
| ILMN_1664330 | CEACAM1 | carcinoembryonic antigen-related cell adhesion molecule 1 | 19 | 0.18 | 0.00082 |  |
| ILMN_2352131 | ERBB2 | v-erb-b2 erythroblastic leukemia viral oncogene homolog 2, neuro/glioblastoma derived oncogene homolog | 17 | 0.183 | 0.00022 | ✔ |
| ILMN_2082593 | KIR3DL3 | killer cell immunoglobulin-like receptor, three domains, long cytoplasmic tail, 3 | 19 | 0.183 | 0.0042 |  |
| ILMN_1796245 | DNASE2 | deoxyribonuclease II, lysosomal | 19 | 0.187 | 0.00001 | ✔ |
| ILMN_1692517 | LOC653381 | PREDICTED: similar to Sorbitol dehydrogenase | 15 | 0.189 | 0.00051 |  |
| ILMN_1750321 | LBA1 | lupus brain antigen 1 | 3 | 0.193 | 0.00202 |  |
| ILMN_1913060 |  | cDNA FLJ44441 fis, clone UTERU2020242 | 12 | 0.193 | 0.00047 |  |
| ILMN_1693826 | HAVCR2 | hepatitis A virus cellular receptor 2 | 5 | 0.194 | 0.00007 | ✔ |
| ILMN_2062468 | IGFBP7 | insulin-like growth factor binding protein 7 | 4 | 0.204 | 0.00012 | ✔ |
| ILMN_2371724 | CEACAM1 | carcinoembryonic antigen-related cell adhesion molecule 1 | 19 | 0.207 | 0.00246 |  |
| ILMN_1721127 | HIST1H3D | histone cluster 1, H3d | 6 | 0.207 | <0.000001 | ✔ |
| ILMN_1796179 | HIST1H2BK | histone cluster 1, H2bk | 6 | 0.209 | 0.0028 | ✔ |
| ILMN_1678882 | KIR2DL1 | killer cell immunoglobulin-like receptor, two domains, long cytoplasmic tail, 1 | 19 | 0.21 | 0.00203 |  |
| ILMN_1745788 | CX3CR1 | chemokine (C-X3-C motif) receptor 1 | 3 | 0.212 | 0.00153 | ✔ |
| ILMN_1784287 | TGFBR3 | transforming growth factor, beta receptor III | 1 | 0.216 | 0.00454 | ✔ |
| ILMN_1779095 | CEBPE | CCAAT/enhancer binding protein | 14 | 0.218 | 0.001 | ✔ |
| ILMN_2131828 | KIR3DL1 | killer cell immunoglobulin-like receptor, three domains, long cytoplasmic tail, 1 | 19 | 0.222 | 0.00253 |  |
| ILMN_1771385 | GBP4 | guanylate binding protein 4 | 1 | 0.225 | 0.00050 | ✔ |
| ILMN_2088437 | CX3CR1 | chemokine (C-X3-C motif) receptor 1 | 3 | 0.235 | 0.00251 | ✔ |
| ILMN_1796409 | C1QB | complement component 1, q subcomponent, B chain | 1 | 0.242 | 0.00066 | ✔ |
| ILMN_1727567 | OLIG2 | oligodendrocyte lineage transcription factor 2 | 21 | 0.254 | 0.00267 | ✔ |
| ILMN_2060413 | CD24 | CD24 molecule | Y | 0.263 | 0.00394 | ✔ |
| ILMN_1778240 | GFOD1 | glucose-fructose oxidoreductase domain containing 1 | 6 | 0.264 | 0.00002 | ✔ |
| ILMN_1716815 | CEACAM1 | carcinoembryonic antigen-related cell adhesion molecule 1 | 19 | 0.265 | 0.00073 |  |
| ILMN_1751120 | HIST1H4H | histone cluster 1, H4h | 6 | 0.266 | 0.00012 | ✔ |
| ILMN_2114568 | GBP5 | guanylate binding protein 5 | 1 | 0.278 | 0.00207 |  |
| ILMN_1677920 | LTF | lactotransferrin | 3 | 0.3 | 0.00181 |  |
| ILMN_1742001 | CD160 | CD160 molecule | 1 | 0.311 | 0.00084 |  |
| ILMN_1682993 | NKG7 | natural killer cell group 7 sequence | 19 | 0.322 | 0.00039 | ✔ |
| ILMN_1713124 | AKR1C3 | aldo-keto reductase family 1, member C3 | 10 | 0.332 | 0.00027 | ✔ |
| ILMN_2109489 | GZMB | granzyme B | 14 | 0.344 | 0.00372 |  |
| ILMN_1758623 | HIST1H2BD | histone cluster 1, H2bd | 6 | 0.346 | 0.00005 | ✔ |
| ILMN_1655549 | SIGLEC10 | sialic acid binding Ig-like lectin 10 | 19 | 0.375 | 0.00001 | ✔ |
| ILMN_1651496 | HIST1H2BD | histone cluster 1, H2bd | 6 | 0.411 | 0.00005 | ✔ |
| ILMN_1688580 | CAMP | cathelicidin antimicrobial peptide | 3 | 0.448 | 0.00324 | ✔ |
| ILMN_2116877 | OLFM4 | olfactomedin 4 | 13 | 0.455 | 0.00049 | ✔ |
| ILMN_2113126 | RNASE3 | ribonuclease, RNase A family, 3 | 14 | 0.475 | 0.00035 |  |
