# Supplementary material for: Transcriptome Profiling of Peripheral Blood in 22q11.2 Deletion Syndrome Reveals Functional Pathways Related to Psychosis and Autism Spectrum Disorder
Source: PLoS One. 2015 Jul 22;10(7):e0132542. doi: 10.1371/journal.pone.0132542 (PMC4511766; doi:10.1371/journal.pone.0132542)
Supplement: S3 Table — (DOCX) [file pone.0132542.s014.docx]

**S3 Table. Differentially expressed probes (significant at 5% FDR) in 22q11DS-PSY+ (N=6) vs. 22q11DS-PSY- (N=40).** Column A: Illumina probe name; B: Gene Symbol; C: Gene definition; D: Chromosome location; E: Log2 fold change (22q11DS-PSY+ vs. 22q11DS-PSY-) shades of red (51-90^th^ percentile of DE genes, with darkest red =90^th^ percentile) are changes >0.2 (corresponding to an absolute fold change of 1.15), in shades of green (10-49^th^ percentile of DE genes, with darkest green=10^th^ percentile) indicate changes < -0.2 (corresponding to an absolute fold change of 0.87); F) p-value after FDR correction; and G) A “**✔** ” is placed next to genes that are brain expressed(3; 17).

| **Probe** | **Symbol** | **Definition** | **Chromo-some** | | **Log Ratio** | ***p*-value** | **Brain Expressed** |
| --- | --- | --- | --- | --- | --- | --- | --- |
| ILMN_1740010 | PCNX | pecanex homolog | | 14 | -0.372 | 0.00233 | ✔ |
| ILMN_1706531 | ABCC5 | ATP-binding cassette, sub-family C, member 5 | | 3 | -0.323 | 0.00171 | ✔ |
| ILMN_1651385 | MFN2 | mitofusin 2 | | 1 | -0.312 | 0.0048 | ✔ |
| ILMN_1657348 | LOC650909 | PREDICTED: similar to activating signal cointegrator 1 complex subunit 3-like 1 | |  | -0.298 | 0.00038 |  |
| ILMN_1784737 | S1PR4 | sphingosine-1-phosphate receptor 4 | | 19 | -0.277 | 0.0044 | ✔ |
| ILMN_1696828 | RGS14 | regulator of G-protein signaling 14 | | 5 | -0.252 | 0.00115 | ✔ |
| ILMN_1732967 | KIAA1949 | KIAA1949 | | 6 | -0.249 | 0.00464 | ✔ |
| ILMN_1705266 | RELA | v-rel reticuloendotheliosis viral oncogene homolog A | | 11 | -0.242 | 0.00374 | ✔ |
| ILMN_1808590 | GUCY1A3 | guanylate cyclase 1, soluble, alpha 3 | | 4 | -0.23 | 0.00493 | ✔ |
| ILMN_1757827 | ECOP | EGFR-coamplified and overexpressed protein | | 7 | -0.224 | 0.00246 |  |
| ILMN_1767573 | CRLF2 | PREDICTED: cytokine receptor-like factor 2 | |  | -0.218 | 0.00244 |  |
| ILMN_1746846 | TTLL4 | tubulin tyrosine ligase-like family, member 4 | | 2 | -0.217 | 0.00195 | ✔ |
| ILMN_1902278 |  | xe08f12.x1 Soares_NFL_T_GBC_S1 cDNA clone IMAGE:2606543 3, mRNA sequence | | 7 | -0.217 | 0.00199 |  |
| ILMN_2064694 | STIM1 | stromal interaction molecule 1 | | 11 | -0.205 | 0.00338 |  |
| ILMN_2152402 | BAT5 | HLA-B associated transcript 5 | | 6 | -0.202 | 0.00216 |  |
| ILMN_1668825 | DKFZp686I15217 | hypothetical protein DKFZp686I15217 | | 6 | -0.199 | 0.00319 |  |
| ILMN_1752088 | ZC4H2 | zinc finger, C4H2 domain containing | | X | -0.196 | 0.00142 | ✔ |
| ILMN_1872568 |  | EST379810 MAGE resequences, MAGJ cDNA, mRNA sequence | |  | -0.195 | 0.00121 |  |
| ILMN_1708983 | CASC1 | cancer susceptibility candidate 1 | | 12 | -0.19 | 0.00438 | ✔ |
| ILMN_2194158 | ADAM29 | ADAM metallopeptidase domain 29 | | 4 | -0.187 | 0.0017 |  |
| ILMN_1657163 | LOC641741 | PREDICTED: hypothetical protein LOC641741 | |  | -0.187 | 0.00244 |  |
| ILMN_2415157 | ARID5A | AT rich interactive domain 5A (MRF1-like) | | 2 | -0.187 | 0.00301 | ✔ |
| ILMN_1651936 | SETD8 | SET domain containing (lysine methyltransferase) 8 | | 12 | -0.185 | 0.00219 | ✔ |
| ILMN_1836570 |  | BX112170 Soares_fetal_heart_NbHH19W cDNA clone IMAGp998O07742, mRNA sequence | | 6 | -0.185 | 0.00275 |  |
| ILMN_1654165 | SLC45A2 | solute carrier family 45, member 2 | | 5 | -0.185 | 0.0035 |  |
| ILMN_2077504 | RNF160 | ring finger protein 160 | | 21 | -0.183 | 0.00384 |  |
| ILMN_1715417 | SELP | selectin P | | 1 | -0.182 | 0.00297 |  |
| ILMN_1681728 | LOC643505 | PREDICTED: similar to large subunit ribosomal protein L36a | | 7 | -0.179 | 0.00436 |  |
| ILMN_2149793 | TTTY10 | testis-specific transcript, Y-linked 10 | | Y | -0.178 | 0.00119 | ✔ |
| ILMN_1670518 | WDFY3 | WD repeat and FYVE domain containing 3 | | 4 | -0.178 | 0.00482 |  |
| ILMN_2129658 | OR2L8 | olfactory receptor, family 2, subfamily L, member 8 | | 1 | -0.17 | 0.00108 | ✔ |
| ILMN_1758871 | DAB1 | disabled homolog 1 | | 1 | -0.167 | 0.00245 | ✔ |
| ILMN_1668334 | LOC641834 | PREDICTED: hypothetical protein LOC641834 | |  | -0.165 | 0.00093 |  |
| ILMN_1701610 | LOC646892 | PREDICTED: similar to SH2 domain protein 2A (T cell-specific adapter protein) (TSAd) (VEGF receptor-associated protein) (SH2 domain-containing adapter protein) | | 15 | -0.164 | 0.00069 |  |
| ILMN_1683300 | BAX | BCL2-associated X protein | | 19 | -0.161 | 0.00275 | ✔ |
| ILMN_1698260 | CCNL2 | cyclin L2 | | 1 | -0.154 | 0.00333 | ✔ |
| ILMN_2391245 | PABPC5 | poly(A) binding protein, cytoplasmic 5 | | X | -0.153 | 0.00289 | ✔ |
| ILMN_2265654 | UBE2C | ubiquitin-conjugating enzyme E2C | | 20 | -0.149 | 0.00173 | ✔ |
| ILMN_1681189 | LOC642154 | PREDICTED: similar to Major urinary protein 5 precursor (MUP 5) | |  | -0.149 | 0.00222 |  |
| ILMN_2370296 | ENAH | enabled homolog | | 1 | -0.149 | 0.00276 | ✔ |
| ILMN_1680139 | MAFF | v-maf musculoaponeurotic fibrosarcoma oncogene homolog F | | 22 | -0.149 | 0.00497 |  |
| ILMN_2200915 | RIPPLY2 | ripply2 homolog | | 6 | -0.147 | 0.00307 |  |
| ILMN_1738233 | AMIGO3 | adhesion molecule with Ig-like domain 3 | | 3 | -0.146 | 0.0028 | ✔ |
| ILMN_1678212 | TMCC1 | transmembrane and coiled-coil domain family 1 | | 3 | -0.146 | 0.00464 | ✔ |
| ILMN_1718836 | SULT4A1 | sulfotransferase family 4A, member 1 | | 22 | -0.145 | 0.00301 | ✔ |
| ILMN_1726682 | LOC146909 | PREDICTED: hypothetical protein LOC146909 | |  | -0.145 | 0.0048 |  |
| ILMN_1749044 | PVRL4 | poliovirus receptor-related 4 | | 1 | -0.143 | 0.00293 | ✔ |
| ILMN_2396697 | GRIK2 | glutamate receptor, ionotropic, kainate 2 | | 6 | -0.142 | 0.002 | ✔ |
| ILMN_1763769 | LOC644065 | PREDICTED: hypothetical LOC644065 | | 5 | -0.142 | 0.00465 |  |
| ILMN_1788629 | CCDC46 | coiled-coil domain containing 46 | | 17 | -0.141 | 0.00293 |  |
| ILMN_1740494 | PCDHA11 | protocadherin alpha 11 | | 5 | -0.141 | 0.00386 |  |
| ILMN_1740305 | DLEC1 | deleted in lung and esophageal cancer 1 | | 3 | -0.14 | 0.00314 | ✔ |
| ILMN_1773082 | CYP21A2 | cytochrome P450, family 21, subfamily A, polypeptide 2 | | 6 | -0.139 | 0.00202 |  |
| ILMN_1706784 | H2AFV | H2A histone family, member V | | 7 | -0.137 | 0.00291 | ✔ |
| ILMN_1652872 | UBOX5 | U-box domain containing 5 | | 20 | -0.137 | 0.00377 | ✔ |
| ILMN_2341132 | TNNT2 | troponin T type 2 | | 1 | -0.137 | 0.00417 | ✔ |
| ILMN_1733023 | CCT6P1 | chaperonin containing TCP1, subunit 6 (zeta) pseudogene 1 | | 7 | -0.136 | 0.00178 |  |
| ILMN_1841564 |  | AGENCOURT_14378000 NIH_MGC_181 cDNA clone IMAGE:30399590 5, mRNA sequence | | 10 | -0.133 | 0.00493 |  |
| ILMN_1890788 |  | Human (clone Z149) retinal mRNA | | 2 | -0.132 | 0.00358 |  |
| ILMN_1813179 | LOC401074 | PREDICTED: hypothetical LOC401074 | |  | -0.131 | 0.00312 |  |
| ILMN_2251505 | ARPP-21 | cyclic AMP-regulated phosphoprotein, 21 kD | | 3 | -0.131 | 0.00323 |  |
| ILMN_1726048 | FLJ27465 | PREDICTED: FLJ27465 protein, transcript variant 1 | | 15 | -0.126 | 0.0041 |  |
| ILMN_1725204 | LOC649859 | PREDICTED: hypothetical protein LOC649859 | |  | 0.118 | 0.00459 |  |
| ILMN_1677137 | C20orf95 | PREDICTED: chromosome 20 open reading frame 95 | |  | 0.122 | 0.00239 |  |
| ILMN_1705637 | ACR | acrosin | | 22 | 0.122 | 0.00354 | ✔ |
| ILMN_1655201 | C21orf70 | chromosome 21 open reading frame 70 | | 21 | 0.129 | 0.00304 |  |
| ILMN_1794407 | LOC646303 | PREDICTED: similar to chromosome Y open reading frame 16 | | Y | 0.129 | 0.00417 |  |
| ILMN_1666656 | KCNK2 | potassium channel, subfamily K, member 2 | | 1 | 0.134 | 0.00291 | ✔ |
| ILMN_2407124 | MCM8 | minichromosome maintenance complex component 8 | | 20 | 0.14 | 0.00366 | ✔ |
| ILMN_1845347 |  | UI-E-EJ1-ajb-c-08-0-UI.r1 UI-E-EJ1 cDNA clone UI-E-EJ1-ajb-c-08-0-UI 5, mRNA sequence | | 5 | 0.14 | 0.00473 |  |
| ILMN_1675013 | MGC22014 | PREDICTED: hypothetical protein MGC22014 | |  | 0.141 | 0.00383 |  |
| ILMN_1727352 | OR4S2 | olfactory receptor, family 4, subfamily S, member 2 | | 11 | 0.143 | 0.00176 |  |
| ILMN_1812787 | LOC653717 | PREDICTED: similar to hect domain and RLD 2, transcript variant 1 | |  | 0.145 | 0.00396 |  |
| ILMN_1666599 | SNORD30 | small nucleolar RNA, C/D box 30 | | 11 | 0.145 | 0.0045 |  |
| ILMN_2207726 | FAM46D | family with sequence similarity 46, member D | | X | 0.149 | 0.00281 |  |
| ILMN_1676237 | ZNF460 | zinc finger protein 460 | | 19 | 0.153 | 0.00299 | ✔ |
| ILMN_1652660 | LOC647357 | PREDICTED: similar to CG14980-PB, transcript variant 1 | |  | 0.154 | 0.00034 |  |
| ILMN_1692696 | LOC644225 | PREDICTED: hypothetical protein LOC644225 | | 17 | 0.155 | 0.00335 |  |
| ILMN_2387742 | HNRNPA1L2 | heterogeneous nuclear ribonucleoprotein A1-like 2 | | 13 | 0.155 | 0.00368 |  |
| ILMN_2053281 | C14orf149 | chromosome 14 open reading frame 149 | | 14 | 0.156 | 0.00351 | ✔ |
| ILMN_1675346 | LOC727811 | similar to chemokine (C-C motif) receptor-like 2 | | 3 | 0.156 | 0.0046 |  |
| ILMN_1675762 | ZNF365 | zinc finger protein 365 | | 10 | 0.157 | 0.002 | ✔ |
| ILMN_1793559 | C6orf204 | chromosome 6 open reading frame 204 | | 6 | 0.158 | 0.00494 | ✔ |
| ILMN_1757038 | LOC93556 | PREDICTED: hypothetical protein BC011266, transcript variant 3 | | 3 | 0.16 | 0.00329 |  |
| ILMN_1711015 | CRYAA | crystallin, alpha A | | 21 | 0.16 | 0.00344 | ✔ |
| ILMN_1779969 | KIAA0738 | PREDICTED: KIAA0738 gene product, transcript variant 3 | |  | 0.161 | 0.00467 |  |
| ILMN_1894895 |  | BX095676 Soares_testis_NHT cDNA clone IMAGp998I154418, mRNA sequence | | 10 | 0.163 | 0.00149 |  |
| ILMN_1800179 | KCNJ4 | potassium inwardly-rectifying channel, subfamily J, member 4 | | 22 | 0.165 | 0.00087 | ✔ |
| ILMN_1685856 | FAM92A1 | family with sequence similarity 92, member A1 | | 8 | 0.165 | 0.00231 | ✔ |
| ILMN_1766757 | LOC286411 | PREDICTED: hypothetical protein LOC286411 | | X | 0.168 | 0.00054 |  |
| ILMN_2350421 | C19orf29 | chromosome 19 open reading frame 29 | | 19 | 0.169 | 0.00249 |  |
| ILMN_1706693 | LOC645689 | PREDICTED: hypothetical protein LOC645689 | | 8 | 0.169 | 0.00483 |  |
| ILMN_1698541 | LOC646997 | PREDICTED: hypothetical protein LOC646997 | | 1 | 0.17 | 0.002 |  |
| ILMN_1790309 | PINX1 | PIN2-interacting protein 1 | | 8 | 0.17 | 0.00212 | ✔ |
| ILMN_1791138 | GPR109B | PREDICTED: G protein-coupled receptor 109B, transcript variant 2 | |  | 0.171 | 0.00326 | ✔ |
| ILMN_1653567 | LOC651308 | PREDICTED: similar to hCG1991759 | |  | 0.172 | 0.00014 |  |
| ILMN_1715180 | LOC643647 | PREDICTED: similar to CG1530-PA | | 2 | 0.174 | 0.0033 |  |
| ILMN_1786172 | LOC653866 | PREDICTED: similar to TFIIH basal transcription factor complex p44 subunit (Basic transcription factor 2 44 kDa subunit) (BTF2-p44) (General transcription factor IIH polypeptide 2), transcript variant 2 | |  | 0.176 | 0.00023 |  |
| ILMN_1726752 | APTX | aprataxin | | 9 | 0.179 | 0.00484 | ✔ |
| ILMN_1670101 | CLEC2D | C-type lectin domain family 2, member D | | 12 | 0.18 | 0.00165 | ✔ |
| ILMN_2218648 | RAG1AP1 | recombination activating gene 1 activating protein 1 | | 1 | 0.18 | 0.00341 |  |
| ILMN_1656619 | LOC652553 | PREDICTED: similar to SLIT-ROBO Rho GTPase-activating protein 2 (srGAP2) (Formin-binding protein 2) | |  | 0.181 | 0.00068 |  |
| ILMN_1775569 | CRISP2 | cysteine-rich secretory protein 2 | | 6 | 0.181 | 0.00128 |  |
| ILMN_1786213 | LOC646198 | PREDICTED: hypothetical protein LOC646198 | | X | 0.181 | 0.00305 |  |
| ILMN_1679921 | CT45A1 | cancer/testis antigen family 45, member A1 | | X | 0.183 | 0.0048 |  |
| ILMN_1673746 | TIMM23 | translocase of inner mitochondrial membrane 23 homolog | | 10 | 0.184 | 0.00313 | ✔ |
| ILMN_2218780 | PPM2C | protein phosphatase 2C, magnesium-dependent, catalytic subunit | | 8 | 0.185 | 0.00365 |  |
| ILMN_2113490 | NTN4 | netrin 4 | | 12 | 0.186 | 0.00013 | ✔ |
| ILMN_1857069 | KIAA1147 | PREDICTED: KIAA1147 | |  | 0.187 | 0.00138 | ✔ |
| ILMN_1751980 | RLBP1 | retinaldehyde binding protein 1 | | 15 | 0.19 | 0.00149 | ✔ |
| ILMN_1764098 | TRNT1 | tRNA nucleotidyl transferase, CCA-adding, 1 | | 3 | 0.191 | 0.00037 | ✔ |
| ILMN_1728048 | LOC158160 | hypothetical protein LOC158160 | | 10 | 0.195 | 0.00278 |  |
| ILMN_2256894 | BTN2A2 | butyrophilin, subfamily 2, member A2 | | 6 | 0.196 | 0.00437 | ✔ |
| ILMN_1697642 | BCAP29 | B-cell receptor-associated protein 29 | | 7 | 0.196 | 0.00468 | ✔ |
| ILMN_1862365 |  | UI-CF-DU1-aao-a-03-0-UI.s1 UI-CF-DU1 cDNA clone UI-CF-DU1-aao-a-03-0-UI 3, mRNA sequence | | 5 | 0.198 | 0.00394 |  |
| ILMN_1749253 | TUBD1 | tubulin, delta 1 | | 17 | 0.199 | 0.00432 | ✔ |
| ILMN_1665192 | NUDT6 | nudix (nucleoside diphosphate linked moiety X)-type motif 6 | | 4 | 0.208 | 9.00E-05 |  |
| ILMN_2335754 | CD1E | CD1e molecule | | 1 | 0.22 | 0.00191 |  |
| ILMN_1707481 | BTBD15 | BTB (POZ) domain containing 15 | | 11 | 0.222 | 0.00253 |  |
| ILMN_1694502 | PRIM1 | primase, DNA, polypeptide 1 (49kDa) | | 12 | 0.224 | 0.00414 | ✔ |
| ILMN_1660819 | FAM7A2 | PREDICTED: family with sequence similarity 7, member A2 | | 15 | 0.23 | 0.00012 |  |
| ILMN_1671742 | UPF3A | UPF3 regulator of nonsense transcripts homolog A | | 13 | 0.234 | 0.00238 | ✔ |
| ILMN_2203768 | PRR15 | proline rich 15 | | 7 | 0.236 | 0.00158 | ✔ |
| ILMN_1799860 | PIGM | phosphatidylinositol glycan anchor biosynthesis, class M | | 1 | 0.246 | 0.00337 | ✔ |
| ILMN_2211672 | TSNAX | translin-associated factor X | | 1 | 0.251 | 0.00051 | ✔ |
| ILMN_2054233 | SENP6 | SUMO1/sentrin specific peptidase 6 | | 6 | 0.255 | 0.00226 | ✔ |
| ILMN_1769633 | CTSO | cathepsin O | | 4 | 0.263 | 0.0014 | ✔ |
| ILMN_1785336 | PMM2 | phosphomannomutase 2 | | 16 | 0.267 | 0.00021 | ✔ |
| ILMN_1756860 | TXNL1 | thioredoxin-like 1 | | 18 | 0.294 | 0.0024 | ✔ |
| ILMN_1714709 | OLFM1 | olfactomedin 1 | | 9 | 0.299 | 0.00041 | ✔ |
| ILMN_1682288 | LOC728565 | PREDICTED: similar to Beta-glucuronidase precursor | | 5 | 0.314 | 0.00233 |  |
| ILMN_1776094 | PPCS | phosphopantothenoylcysteine synthetase | | 1 | 0.317 | 0.00447 | ✔ |
| ILMN_1655195 | SMA4 | SMA4 | | 5 | 0.336 | 0.00199 |  |
| ILMN_1759628 | ATP1B3 | PREDICTED: ATPase, Na+/K+ transporting, beta 3 polypeptide, transcript variant 2 | |  | 0.341 | 0.00324 | ✔ |
| ILMN_1719749 | PTGES3 | prostaglandin E synthase 3 | | 12 | 0.375 | 0.00439 |  |
| ILMN_1720857 | GUSBL1 | glucuronidase, beta-like 1 | | 6 | 0.398 | 0.00041 |  |
| ILMN_2133534 | SMA4 | SMA4 | |  | 0.405 | 0.00013 |  |
| ILMN_1799381 | SNORD14A | small nucleolar RNA, C/D box 14A | | 11 | 0.423 | 0.00233 |  |
| ILMN_1759954 | PTMA | prothymosin, alpha | | 2 | 0.441 | 0.00342 | ✔ |
| ILMN_1742025 | OLFM1 | olfactomedin 1 | | 9 | 0.545 | 0.00012 | ✔ |
| ILMN_2136133 | PABPC1 | poly(A) binding protein, cytoplasmic 1 | | 8 | 0.696 | 0.00233 |  |
