# Supplementary material for: Transcriptome Profiling of Peripheral Blood in 22q11.2 Deletion Syndrome Reveals Functional Pathways Related to Psychosis and Autism Spectrum Disorder
Source: PLoS One. 2015 Jul 22;10(7):e0132542. doi: 10.1371/journal.pone.0132542 (PMC4511766; doi:10.1371/journal.pone.0132542)
Supplement: S4 Table — (DOCX) [file pone.0132542.s015.docx]

**S4 Table. Differentially expressed probes significant (at 5% FDR) in 22q11DS-ASD+ (N=15) vs. 22q11DS-ASD- (N=25).** Column A: Illumina probe name; B: Gene Symbol; C: Gene definition; D: Chromosome location; E: Log2 fold change (22q11DS-ASD+ vs. 22q11DS-ASD-) shades of red (51-90^th^ percentile of DE genes, with darkest red =90^th^ percentile) are changes >0.2 (corresponding to an absolute fold change of 1.15), in shades of green (10-49^th^ percentile of DE genes, with darkest green=10^th^ percentile) indicate changes < -0.2 (corresponding to an absolute fold change of 0.87); F) p-value after correction for false discovery rate and G) A “**✔** ” is placed next to genes that are brain expressed(4).

| **Probe** | **Symbol** | **Gene Name** | **Chromo-some** | **Log Ratio** | ***p*-value** | **Brain Expressed** |
| --- | --- | --- | --- | --- | --- | --- |
| ILMN_1806651 | PARP8 | poly (ADP-ribose) polymerase family, member 8 | 5 | -0.32 | 0.00001 | ✔ |
| ILMN_1762436 | UBB | ubiquitin B | 17 | -0.3 | 0.00226 | ✔ |
| ILMN_1686388 | LOC644330 | PREDICTED: similar to tropomyosin 3 isoform 2 | 19 | -0.29 | 0.00163 |  |
| ILMN_1761797 | CSTB | cystatin B | 21 | -0.25 | 0.00006 | ✔ |
| ILMN_1675386 | CES1 | carboxylesterase 1 | 16 | -0.2 | 0.00289 |  |
| ILMN_1678312 | LOC388397 | PREDICTED: hypothetical LOC388397 |  | -0.19 | 0.00014 |  |
| ILMN_1719518 | ARF4 | ADP-ribosylation factor 4 | 3 | -0.15 | 0.0044 | ✔ |
| ILMN_1686516 | CUGBP1 | CUG triplet repeat, RNA binding protein 1 | 11 | -0.15 | 0.00096 |  |
| ILMN_1804845 | OR4P4 | olfactory receptor, family 4, subfamily P, member 4 | 11 | -0.14 | 0.00014 |  |
| ILMN_1775622 | DMP1 | dentin matrix acidic phosphoprotein | 4 | -0.14 | 0.00105 |  |
| ILMN_1682054 | SRI | sorcin | 7 | -0.14 | 0.00477 | ✔ |
| ILMN_1719753 | VGLL1 | vestigial like 1 | X | -0.13 | 0.00038 | ✔ |
| ILMN_1777182 | AGC1 | aggrecan 1 (chondroitin sulfate proteoglycan 1, large aggregating proteoglycan, antigen identified by monoclonal antibody A0122) | 15 | -0.13 | 0.00285 |  |
| ILMN_2396948 | PSMC3IP | PSMC3 interacting protein | 17 | -0.13 | 0.00156 |  |
| ILMN_1697412 | RHBG | Rh family, B glycoprotein | 1 | -0.13 | 0.00148 | ✔ |
| ILMN_1751289 | DERPC | decreased expression in renal and prostate | 16 | -0.13 | 0.00479 |  |
| ILMN_1798547 | FAM71D | family with sequence similarity 71, member D | 14 | -0.13 | 0.00157 |  |
| ILMN_2288254 | UNC45A | unc-45 homolog A | 15 | -0.13 | 0.00137 | ✔ |
| ILMN_1667692 | PTGIS | prostaglandin I2 (prostacyclin) synthase | 20 | -0.12 | 0.00267 | ✔ |
| ILMN_1653372 | ATP8B3 | ATPase, class I, type 8B, member 3 | 19 | -0.12 | 0.00467 | ✔ |
| ILMN_1780143 | LOC653641 | PREDICTED: similar to Golgi autoantigen, golgin subfamily A member 6 | 15 | -0.12 | 0.00103 |  |
| ILMN_1749675 | WNT8B | wingless-type MMTV integration site family, member 8B | 10 | -0.12 | 0.00053 | ✔ |
| ILMN_2241750 | KALRN | kalirin, RhoGEF kinase | 3 | -0.12 | 0.00133 | ✔ |
| ILMN_1687400 | MAGEA4 | melanoma antigen family A, 4 | X | -0.12 | 0.00488 |  |
| ILMN_1737213 | LOC653641 | PREDICTED: similar to Golgin subfamily A member 6 | 15 | -0.12 | 0.00101 |  |
| ILMN_1673005 | C20orf194 | PREDICTED: chromosome 20 open reading frame 194 |  | -0.11 | 0.00281 |  |
| ILMN_1770260 | NFKBIZ | nuclear factor of kappa light polypeptide gene enhancer in B-cells inhibitor, zeta | 3 | -0.11 | 0.00366 | ✔ |
| ILMN_1709075 | OLA1 | Obg-like ATPase 1 | 2 | -0.11 | 0.00047 | ✔ |
| ILMN_2335319 | KCNG3 | potassium voltage-gated channel, subfamily G, member 3 | 2 | -0.11 | 0.00314 | ✔ |
| ILMN_1752884 | AADACL2 | arylacetamide deacetylase-like 2 | 3 | -0.11 | 0.00232 |  |
| ILMN_1723834 | FLJ32011 | hypothetical protein FLJ32011 | 1 | -0.11 | 0.0039 |  |
| ILMN_1765212 | LARP1B | La ribonucleoprotein domain family, member 1B | 4 | -0.11 | 0.00273 | ✔ |
| ILMN_1672765 | LOC648827 | PREDICTED: hypothetical protein LOC648827 |  | -0.11 | 0.00418 |  |
| ILMN_2283094 | FRMPD2L2 | FERM and PDZ domain containing 2 like 2 | 10 | -0.11 | 0.00133 |  |
| ILMN_1707591 | TNIP3 | TNFAIP3 interacting protein 3 | 4 | -0.11 | 0.00388 |  |
| ILMN_1680732 | B4GALT2 | UDP-Gal:betaGlcNAc beta 1,4- galactosyltransferase, polypeptide 2 | 1 | -0.11 | 0.00163 | ✔ |
| ILMN_2043126 | CSAG3A | CSAG family, member 3A | X | -0.11 | 0.00358 |  |
| ILMN_1800711 | DEPDC5 | DEP domain containing 5 | 22 | -0.11 | 0.00337 | ✔ |
| ILMN_1661903 | CYLC2 | cylicin, basic protein of sperm head cytoskeleton 2 | 9 | -0.1 | 0.00172 |  |
| ILMN_1675992 | DST | dystonin | 6 | -0.1 | 0.00499 |  |
| ILMN_1670707 | C17orf46 | chromosome 17 open reading frame 46 | 17 | -0.1 | 0.00251 | ✔ |
| ILMN_1727144 | BIRC8 | baculoviral IAP repeat-containing 8 | 19 | -0.1 | 0.00243 |  |
| ILMN_1681242 | BLOC1S3 | biogenesis of lysosome-related organelles complex-1, subunit 3 | 19 | -0.1 | 0.00446 | ✔ |
| ILMN_1810131 | FAM123A | family with sequence similarity 123A | 13 | -0.1 | 0.00273 | ✔ |
| ILMN_2078995 | CSRNP3 | cysteine-serine-rich nuclear protein 3 | 2 | -0.1 | 0.00385 |  |
| ILMN_2091978 | FREM2 | FRAS1 related extracellular matrix protein 2 | 13 | -0.1 | 0.00276 | ✔ |
| ILMN_1667933 | LOC646428 | PREDICTED: hypothetical protein LOC646428 | 9 | -0.1 | 0.00226 |  |
| ILMN_2140191 | OR2M3 | olfactory receptor, family 2, subfamily M, member 3 | 1 | -0.1 | 0.00471 |  |
| ILMN_1728894 | OR4A5 | olfactory receptor, family 4, subfamily A, member 5 | 11 | -0.1 | 0.00397 |  |
| ILMN_1707957 | LOC644738 | PREDICTED: hypothetical protein LOC644738 | 3 | -0.09 | 0.00104 |  |
| ILMN_1662824 | MADCAM1 | mucosal vascular addressin cell adhesion molecule 1 | 19 | -0.09 | 0.00187 | ✔ |
| ILMN_1812256 | GANC | glucosidase, alpha; neutral C | 15 | -0.09 | 0.00336 |  |
| ILMN_1779488 | RFX3 | regulatory factor X, 3 (influences HLA class II expression) | 9 | -0.09 | 0.00291 | ✔ |
| ILMN_1678627 | RUFY4 | RUN and FYVE domain containing 4 | 2 | -0.08 | 0.00377 | ✔ |
| ILMN_1786654 | LOC644251 | PREDICTED: similar to splicing coactivator subunit SRm300 | 10 | 0.087 | 0.00437 |  |
| ILMN_2058570 | XGPY2 | Xg pseudogene, Y-linked 2 | Y | 0.091 | 0.00328 |  |
| ILMN_1741168 | SPRY4 | sprouty homolog 4 | 5 | 0.093 | 0.00441 | ✔ |
| ILMN_1759708 | LOC643959 | PREDICTED: hypothetical protein LOC643959 | 15 | 0.097 | 0.00252 |  |
| ILMN_1888330 |  | wf30f02.x1 Soares_NFL_T_GBC_S1 cDNA clone IMAGE:2357115 3, mRNA sequence | 11 | 0.098 | 0.00351 |  |
| ILMN_1731349 | HOXA13 | homeobox A13 |  | 0.099 | 0.00212 |  |
| ILMN_1824196 |  | UI-H-BI4-apj-b-11-0-UI.s1 NCI_CGAP_Sub8 cDNA clone IMAGE:3087333 3, mRNA sequence | X | 0.105 | 0.00287 |  |
| ILMN_1656865 | LOC645602 | PREDICTED: hypothetical LOC645602 |  | 0.106 | 0.00252 |  |
| ILMN_2205622 | MUC2 | mucin 2, oligomeric mucus/gel-forming | 11 | 0.106 | 0.00426 |  |
| ILMN_1777608 | LOC646145 | PREDICTED: similar to cis-Golgi matrix protein GM130 | 15 | 0.107 | 0.00493 |  |
| ILMN_2355004 | HMGN3 | high mobility group nucleosomal binding domain 3 | 6 | 0.108 | 0.00353 | ✔ |
| ILMN_1745529 | KCNH1 | potassium voltage-gated channel, subfamily H (eag-related), member 1 | 1 | 0.11 | 0.00226 | ✔ |
| ILMN_1683849 | LOC644567 | PREDICTED: similar to Glutamate dehydrogenase 1, mitochondrial precursor (GDH) | 10 | 0.11 | 0.00115 |  |
| ILMN_1850240 |  | xq93b01.x1 NCI_CGAP_Brn53 cDNA clone IMAGE:2758153 3, mRNA sequence | 14 | 0.11 | 0.00074 |  |
| ILMN_1682831 | NEB | nebulin | 2 | 0.111 | 0.00393 | ✔ |
| ILMN_1889884 | LOC730118 | PREDICTED: hypothetical LOC730118 | 14 | 0.113 | 0.0043 |  |
| ILMN_1752415 | LOC644714 | PREDICTED: hypothetical protein LOC644714 |  | 0.114 | 0.00308 |  |
| ILMN_1727916 | FLJ46380 | FLJ46380 protein | 1 | 0.116 | 0.00173 |  |
| ILMN_1717706 | PLK2 | polo-like kinase 2 | 5 | 0.116 | 0.00167 | ✔ |
| ILMN_1682724 | FANCF | Fanconi anemia, complementation group F | 11 | 0.117 | 0.00325 | ✔ |
| ILMN_1734550 | SLC9A3 | solute carrier family 9 (sodium/hydrogen exchanger), member 3 | 5 | 0.117 | 0.00359 | ✔ |
| ILMN_1789410 | ZSCAN21 | zinc finger and SCAN domain containing 21 | 7 | 0.117 | 0.00466 | ✔ |
| ILMN_2077160 | C9orf41 | chromosome 9 open reading frame 41 | 9 | 0.118 | 0.00499 |  |
| ILMN_1652602 | C11orf35 | chromosome 11 open reading frame 35 | 11 | 0.119 | 0.002 |  |
| ILMN_1734234 | LILRB2 | leukocyte immunoglobulin-like receptor, subfamily B |  | 0.119 | 0.00298 | ✔ |
| ILMN_1794452 | LOC652331 | PREDICTED: similar to complement factor H-related 1 |  | 0.119 | 0.00346 |  |
| ILMN_1678459 | VAPB | VAMP (vesicle-associated membrane protein)-associated protein B and C | 20 | 0.12 | 0.00284 | ✔ |
| ILMN_1916311 |  | DB069547 TESTI4 cDNA clone TESTI4011813 5, mRNA sequence | 1 | 0.121 | 0.00197 |  |
| ILMN_1685446 | NARG1L | NMDA receptor regulated 1-like | 13 | 0.124 | 0.00195 |  |
| ILMN_2314140 | PAX6 | paired box 6 | 11 | 0.124 | 0.00337 | ✔ |
| ILMN_1653670 | CUL3 | cullin 3 | 2 | 0.125 | 0.00085 | ✔ |
| ILMN_1743301 | MSR1 | macrophage scavenger receptor 1 | 8 | 0.125 | 0.0046 |  |
| ILMN_1810166 | SERPINA2 | PREDICTED: serpin peptidase inhibitor, clade A (alpha-1 antiproteinase, antitrypsin), member 2 | 14 | 0.126 | 0.00202 |  |
| ILMN_1703973 | LOC654002 | PREDICTED: similar to BMS1-like, ribosome assembly protein, transcript variant 3 |  | 0.127 | 0.00256 |  |
| ILMN_1710329 | MYEF2 | myelin expression factor 2 | 15 | 0.128 | 0.00232 | ✔ |
| ILMN_1769580 | PRSS35 | protease, serine, 35 | 6 | 0.128 | 0.00117 |  |
| ILMN_1850892 |  | BX102680 Soares infant brain 1NIB cDNA clone IMAGp998H0374, mRNA sequence | 19 | 0.128 | 0.0007 |  |
| ILMN_1662766 | LOC647439 | PREDICTED: hypothetical protein LOC647439 |  | 0.13 | 0.00292 |  |
| ILMN_1818562 |  | cDNA clone IMAGE:5267453 | 8 | 0.13 | 0.00218 |  |
| ILMN_1683296 | LOC650836 | PREDICTED: similar to T-cell receptor alpha chain V region PHDS58 precursor |  | 0.131 | 0.00042 |  |
| ILMN_1791067 | TESK1 | testis-specific kinase 1 | 9 | 0.133 | 0.00458 | ✔ |
| ILMN_1675671 | ICOSLG | inducible T-cell co-stimulator ligand | 21 | 0.134 | 0.00406 | ✔ |
| ILMN_1751280 | LOC651905 | PREDICTED: hypothetical protein LOC651905 |  | 0.134 | 0.00043 |  |
| ILMN_1676689 | PPT2 | palmitoyl-protein thioesterase 2 | 6 | 0.135 | 0.00061 | ✔ |
| ILMN_1889669 |  | DB160069 THYMU3 cDNA clone THYMU3040068 5, mRNA sequence | 17 | 0.135 | 0.00064 |  |
| ILMN_1884015 |  | 603183417F1 NIH_MGC_121 cDNA clone IMAGE:5247269 5, mRNA sequence | 6 | 0.137 | 0.00079 |  |
| ILMN_1846799 |  | zo26g12.x5 Stratagene colon (#937204) cDNA clone IMAGE:588070 3, mRNA sequence | 11 | 0.137 | 0.0046 |  |
| ILMN_1765082 | RBM10 | RNA binding motif protein 10 | X | 0.139 | 0.0009 | ✔ |
| ILMN_1795564 | C11orf84 | chromosome 11 open reading frame 84 | 11 | 0.14 | 0.00484 | ✔ |
| ILMN_1783676 | CCDC15 | coiled-coil domain containing 15 | 11 | 0.14 | 0.0046 |  |
| ILMN_1787366 | ZNF335 | zinc finger protein 335 | 20 | 0.141 | 0.00404 | ✔ |
| ILMN_2388539 | C17orf101 | chromosome 17 open reading frame 101 | 17 | 0.143 | 0.00047 |  |
| ILMN_1739779 | LOC652742 | PREDICTED: similar to Low affinity immunoglobulin gamma Fc region receptor II-c precursor (Fc-gamma RII-c) (FcRII-c) (IgG Fc receptor II-c) (Fc-gamma-RIIc) (CD32 antigen) (CDw32) |  | 0.143 | 0.00001 |  |
| ILMN_1838667 |  | ws92f09.x1 NCI_CGAP_Co3 cDNA clone IMAGE:2505449 3, mRNA sequence | 1 | 0.15 | 0.00011 |  |
| ILMN_1814661 | PHLPP1 | PH domain and leucine rich repeat protein phosphatase 1 | 18 | 0.156 | 0.00412 | ✔ |
| ILMN_2218002 | LIPC | lipase, hepatic | 15 | 0.163 | 0.00102 |  |
| ILMN_2211790 | ADAMTS7 | ADAM metallopeptidase with thrombospondin type 1 motif, 7 | 15 | 0.169 | 0.00005 | ✔ |
| ILMN_1891109 |  | BX118761 Soares_testis_NHT cDNA clone IMAGp998O061863, mRNA sequence | 20 | 0.17 | 0.00001 |  |
| ILMN_2203768 | PRR15 | proline rich 15 | 7 | 0.173 | 0.00186 | ✔ |
| ILMN_1782861 | C10orf82 | chromosome 10 open reading frame 82 | 10 | 0.175 | 0.0009 | ✔ |
| ILMN_1720513 | SETBP1 | SET binding protein 1 | 18 | 0.18 | 0.00051 | ✔ |
| ILMN_1898723 |  | cDNA clone IMAGE:3079901 | X | 0.19 | 0.00388 |  |
| ILMN_1676003 | PNOC | prepronociceptin | 8 | 0.23 | 0.00469 | ✔ |
| ILMN_1742544 | MEF2C | myocyte enhancer factor 2C | 5 | 0.25 | 0.00244 | ✔ |
| ILMN_1773567 | LAMA5 | laminin, alpha 5 | 20 | 0.262 | 0.00127 | ✔ |
| ILMN_2414762 | TLR10 | toll-like receptor 10 | 4 | 0.265 | 0.00024 |  |
| ILMN_1719905 | TLR10 | toll-like receptor 10 | 4 | 0.27 | 0.00112 |  |
| ILMN_1724295 | LOC643007 | PREDICTED: similar to large subunit ribosomal protein L36a |  | 0.277 | 0.0023 |  |
| ILMN_2374352 | DBNDD1 | dysbindin (dystrobrevin binding protein 1) domain containing 1 | 16 | 0.278 | 0.00434 | ✔ |
| ILMN_1723004 | CD72 | CD72 molecule | 9 | 0.32 | 0.00112 |  |
| ILMN_1794927 | LOC90925 | hypothetical protein LOC90925 |  | 0.363 | 0.00377 |  |
| ILMN_2337928 | CXCR5 | chemokine (C-X-C motif) receptor 5 | 11 | 0.374 | 0.00201 |  |
| ILMN_1700428 | HLA-DOB | major histocompatibility complex, class II, DO beta | 6 | 0.398 | 0.00097 |  |
| ILMN_2366212 | CD79B | CD79b molecule, immunoglobulin-associated beta | 17 | 0.407 | 0.00483 | ✔ |
| ILMN_1710017 | CD79B | CD79B antigen, immunoglobulin-associated beta | 17 | 0.439 | 0.00228 | ✔ |
| ILMN_1691071 | FCRLA | Fc receptor-like A | 1 | 0.439 | 0.00211 | ✔ |
| ILMN_1662451 | FCER2 | Fc fragment of IgE, low affinity II, receptor for (CD23) | 19 | 0.452 | 0.00097 |  |
