# Supplementary material for: Transcriptome Profiling of Peripheral Blood in 22q11.2 Deletion Syndrome Reveals Functional Pathways Related to Psychosis and Autism Spectrum Disorder
Source: PLoS One. 2015 Jul 22;10(7):e0132542. doi: 10.1371/journal.pone.0132542 (PMC4511766; doi:10.1371/journal.pone.0132542)
Supplement: S5 Table — (DOCX) [file pone.0132542.s016.docx]

**S5 Table. List of genes in the Purple module, which was significantly associated with psychotic disorder diagnosis in 22q11DS.** Column A: Illumina probe name; B: Gene Symbol; C: Gene definition; D: Chromosome location; E: Eigengene correlation value (range 0-1); F: Eigengene *p*-value; G) A “**✔** ” is placed next to genes that are considered to be brain expressed (12; 18). Probes that are highlighted in bold and have an asterisk after the Gene Symbol are probes that overlapped between the Purple module and DE genes in 22q11DS-PSY+ (as shown in eTable 4).

| **Probe** | **Symbol** | **Gene Name** | **Chromo-some** | **Eigen-gene Corre-lation** | **Eigengene**  **p-value** | **Brain Expressed** |
| --- | --- | --- | --- | --- | --- | --- |
| ILMN_2361570 | SNX14 | sorting nexin 14 | 6 | 0.899 | 2.41E-17 | ✔ |
| ILMN_2367428 | FAM96A | family with sequence similarity 96, member A | 15 | 0.887 | 2.51E-16 | **✔** |
| ILMN_1776337 | CHORDC1 | cysteine and histidine-rich domain (CHORD)-containing 1 | 11 | 0.886 | 2.86E-16 | ✔ |
| ILMN_2135272 | GIMAP2 | GTPase, IMAP family member 2 | 7 | 0.884 | 3.93E-16 | ✔ |
| **ILMN_2396996** | **PPCS*** | **phosphopantothenoylcysteine synthetase** | **1** | **0.882** | **6.08E-16** | **✔** |
| ILMN_2169761 | CPNE8 | copine VIII | 12 | 0.867 | 6.37E-15 | ✔ |
| ILMN_2104784 | FKBP3 | FK506 binding protein 3, 25kDa | 14 | 0.866 | 7.63E-15 | ✔ |
| ILMN_1747630 | DEK | DEK oncogene (DNA binding) | 6 | 0.863 | 1.16E-14 | ✔ |
| ILMN_1687785 | PPA2 | pyrophosphatase (inorganic) 2 | 4 | 0.862 | 1.34E-14 | ✔ |
| ILMN_1761120 | TM2D3 | TM2 domain containing 3 | 15 | 0.862 | 1.42E-14 | ✔ |
| ILMN_1810838 | MTDH | metadherin | 8 | 0.855 | 4.15E-14 | ✔ |
| ILMN_1734696 | FRG1 | FSHD region gene 1 | 4 | 0.854 | 4.75E-14 | ✔ |
| ILMN_1725244 | HAT1 | histone acetyltransferase 1 | 2 | 0.853 | 5.46E-14 | ✔ |
| ILMN_1771835 | NUP54 | nucleoporin 54kDa | 4 | 0.846 | 1.31E-13 | ✔ |
| ILMN_1734740 | GPR65 | G protein-coupled receptor 65 | 14 | 0.845 | 1.62E-13 | ✔ |
| ILMN_1756104 | LARP7 | La ribonucleoprotein domain family, member 7 | 4 | 0.845 | 1.64E-13 | ✔ |
| ILMN_2061043 | CD48 | CD48 molecule | 1 | 0.840 | 2.78E-13 |  |
| ILMN_2232121 | GPR65 | G protein-coupled receptor 65 | 14 | 0.840 | 3.05E-13 | ✔ |
| ILMN_1732216 | NARS | asparaginyl-tRNA synthetase | 18 | 0.839 | 3.17E-13 | ✔ |
| ILMN_2331163 | CUL4A | cullin 4A | 13 | 0.839 | 3.42E-13 | ✔ |
| ILMN_1724230 | LOC642236 | PREDICTED: similar to FRG1 protein (FSHD region gene 1 protein) |  | 0.835 | 5.73E-13 |  |
| ILMN_1658182 | MEX3C | mex-3 homolog C | 18 | 0.833 | 7.05E-13 | ✔ |
| ILMN_1683888 | SRP72 | signal recognition particle 72kDa | 4 | 0.832 | 7.64E-13 | ✔ |
| ILMN_1670439 | FYTTD1 | forty-two-three domain containing 1 | 3 | 0.832 | 7.77E-13 | ✔ |
| ILMN_2151541 | DNAJC10 | DnaJ (Hsp40) homolog, subfamily C, member 10 | 2 | 0.832 | 7.92E-13 | ✔ |
| ILMN_2205211 | LOC134997 | peptidylprolyl isomerase A processed pseudogene | 6 | 0.831 | 9.28E-13 |  |
| ILMN_1814113 | ZFR | zinc finger RNA binding protein | 5 | 0.829 | 1.06E-12 | ✔ |
| ILMN_2226324 | BRP44L | brain protein 44-like | 6 | 0.829 | 1.07E-12 | ✔ |
| ILMN_2056167 | OSTC | oligosaccharyltransferase complex subunit | 4 | 0.829 | 1.15E-12 | ✔ |
| ILMN_1696708 | C14orf166 | chromosome 14 open reading frame 166 | 14 | 0.828 | 1.29E-12 |  |
| ILMN_2091375 | KRCC1 | lysine-rich coiled-coil 1 | 2 | 0.825 | 1.82E-12 | ✔ |
| ILMN_2231242 | HMGB1 | high-mobility group box 1 | 13 | 0.824 | 2.11E-12 |  |
| ILMN_1763539 | IER3IP1 | immediate early response 3 interacting protein 1 | 18 | 0.823 | 2.27E-12 | ✔ |
| ILMN_2192694 | EIF3M | eukaryotic translation initiation factor 3, subunit M | 11 | 0.820 | 2.99E-12 | ✔ |
| ILMN_2358277 | RSRC2 | arginine/serine-rich coiled-coil 2 | 12 | 0.818 | 3.77E-12 | ✔ |
| ILMN_1759983 | DR1 | down-regulator of transcription 1, TBP-binding (negative cofactor 2) | 1 | 0.816 | 4.82E-12 | ✔ |
| ILMN_1695640 | PTPN22 | protein tyrosine phosphatase, non-receptor type 22 | 1 | 0.815 | 5.64E-12 | ✔ |
| ILMN_1698487 | SDHD | succinate dehydrogenase complex, subunit D, integral membrane protein | 11 | 0.812 | 7.36E-12 | ✔ |
| ILMN_1675669 | IBTK | inhibitor of Bruton agammaglobulinemia tyrosine kinase | 6 | 0.812 | 7.39E-12 | ✔ |
| ILMN_2322935 | MAPKAPK5 | mitogen-activated protein kinase-activated protein kinase 5 | 12 | 0.812 | 7.43E-12 | ✔ |
| ILMN_2246328 | PTPN22 | protein tyrosine phosphatase, non-receptor type 22 | 1 | 0.810 | 9.00E-12 | ✔ |
| ILMN_2059294 | RTCD1 | RNA terminal phosphate cyclase domain 1 | 1 | 0.810 | 9.09E-12 | ✔ |
| ILMN_2108339 | THUMPD1 | THUMP domain containing 1 | 16 | 0.810 | 9.35E-12 | ✔ |
| ILMN_2387553 | PSMA3 | proteasome (prosome, macropain) subunit, alpha type, 3 | 14 | 0.806 | 1.35E-11 | ✔ |
| ILMN_2204297 | MUDENG | MU-2/AP1M2 domain containing, death-inducing | 14 | 0.806 | 1.45E-11 | ✔ |
| ILMN_2186482 | TMED7 | transmembrane emp24 protein transport domain containing 7 | 5 | 0.806 | 1.47E-11 |  |
| ILMN_1694177 | PCNA | proliferating cell nuclear antigen | 20 | 0.805 | 1.58E-11 | ✔ |
| ILMN_1767837 | GOLT1B | golgi transport 1 homolog B | 12 | 0.802 | 2.07E-11 | ✔ |
| ILMN_2167011 | ECHDC1 | enoyl Coenzyme A hydratase domain containing 1 | 6 | 0.802 | 2.20E-11 | ✔ |
| ILMN_2318011 | PSMA3 | proteasome (prosome, macropain) subunit, alpha type, 3 | 14 | 0.801 | 2.22E-11 | ✔ |
| ILMN_1749821 | MED28 | mediator complex subunit 28 | 4 | 0.800 | 2.63E-11 | ✔ |
| ILMN_2170353 | PTPLB | protein tyrosine phosphatase-like (proline instead of catalytic arginine), member b | 3 | 0.799 | 2.70E-11 | ✔ |
| ILMN_1737396 | PSMD14 | proteasome (prosome, macropain) 26S subunit, non-ATPase, 14 | 2 | 0.799 | 2.85E-11 | ✔ |
| ILMN_2344650 | N4BP2L1 | NEDD4 binding protein 2-like 1 | 13 | 0.798 | 3.15E-11 |  |
| ILMN_1814213 | PQLC3 | PQ loop repeat containing 3 | 2 | 0.798 | 3.16E-11 | ✔ |
| ILMN_1702265 | HDHD2 | haloacid dehalogenase-like hydrolase domain containing 2 | 18 | 0.797 | 3.30E-11 | ✔ |
| ILMN_1738150 | SUMO2 | SMT3 suppressor of mif two 3 homolog 2 | 17 | 0.796 | 3.67E-11 | ✔ |
| ILMN_1745423 | UTP3 | UTP3, small subunit (SSU) processome component, homolog | 4 | 0.795 | 4.35E-11 | ✔ |
| ILMN_2230672 | MRPL18 | mitochondrial ribosomal protein L18 | 6 | 0.794 | 4.65E-11 | ✔ |
| ILMN_2356838 | CEPT1 | choline/ethanolamine phosphotransferase 1 | 1 | 0.792 | 5.54E-11 | ✔ |
| ILMN_2223130 | SMARCA5 | SWI/SNF related, matrix associated, actin dependent regulator of chromatin, subfamily a, member 5 | 4 | 0.791 | 5.95E-11 | ✔ |
| ILMN_1767747 | HDAC2 | histone deacetylase 2 | 6 | 0.789 | 7.04E-11 | ✔ |
| ILMN_1729019 | SEPT7 | septin 7 | 7 | 0.789 | 7.06E-11 | **✔** |
| ILMN_1713178 | FAM116A | PREDICTED: family with sequence similarity 116, member A |  | 0.785 | 1.05E-10 | ✔ |
| ILMN_1768117 | RBM25 | RNA binding motif protein 25 | 14 | 0.783 | 1.30E-10 | ✔ |
| ILMN_1803799 | LOC649555 | PREDICTED: similar to eukaryotic translation initiation factor 4E |  | 0.782 | 1.37E-10 |  |
| ILMN_1782488 | RNASEH2B | ribonuclease H2, subunit B | 13 | 0.781 | 1.48E-10 | ✔ |
| ILMN_1735453 | FAM98A | family with sequence similarity 98, member A | 2 | 0.780 | 1.69E-10 | ✔ |
| ILMN_1708936 | EXOSC3 | exosome component 3 | 9 | 0.780 | 1.72E-10 | ✔ |
| ILMN_2347917 | EED | embryonic ectoderm development | 11 | 0.779 | 1.76E-10 | ✔ |
| ILMN_2211800 | HMGB1L1 | high-mobility group box 1-like 1 | 20 | 0.778 | 1.91E-10 |  |
| ILMN_1712766 | ERGIC2 | ERGIC and golgi 2 | 12 | 0.778 | 1.96E-10 | ✔ |
| ILMN_1718853 | UQCRC2 | ubiquinol-cytochrome c reductase core protein II | 16 | 0.777 | 2.19E-10 | ✔ |
| ILMN_1733680 | AZI2 | 5-azacytidine induced 2 | 3 | 0.776 | 2.39E-10 | ✔ |
| ILMN_1664577 | DLD | dihydrolipoamide dehydrogenase | 7 | 0.775 | 2.55E-10 | ✔ |
| ILMN_1814526 | ADD3 | adducin 3 | 10 | 0.773 | 3.10E-10 | ✔ |
| ILMN_1769810 | ARL6IP5 | ADP-ribosylation-like factor 6 interacting protein 5 | 3 | 0.772 | 3.23E-10 | ✔ |
| ILMN_1745620 | KRCC1 | lysine-rich coiled-coil 1 | 2 | 0.772 | 3.45E-10 | ✔ |
| ILMN_1691097 | HSP90AA1 | heat shock protein 90kDa alpha (cytosolic), class A member 1 | 14 | 0.771 | 3.64E-10 | ✔ |
| ILMN_1701434 | RAP1B | RAP1B, member of RAS oncogene family | 12 | 0.770 | 3.82E-10 | ✔ |
| ILMN_1661485 | RBM34 | RNA binding motif protein 34 | 1 | 0.770 | 3.83E-10 |  |
| ILMN_2113074 | UFM1 | ubiquitin-fold modifier 1 | 13 | 0.770 | 4.10E-10 | ✔ |
| ILMN_1751234 | C1GALT1C1 | C1GALT1-specific chaperone 1 | X | 0.769 | 4.35E-10 | ✔ |
| ILMN_1754839 | DHX15 | DEAH (Asp-Glu-Ala-His) box polypeptide 15 | 4 | 0.768 | 4.68E-10 | ✔ |
| ILMN_1898682 |  | mRNA; cDNA DKFZp779F0411 | 6 | 0.767 | 5.13E-10 |  |
| ILMN_2187830 | CCNH | cyclin H | 5 | 0.765 | 5.93E-10 | **✔** |
| ILMN_1657436 | FGFR1OP2 | FGFR1 oncogene partner 2 | 12 | 0.765 | 5.94E-10 | ✔ |
| ILMN_2094942 | MAR1 | membrane-associated ring finger (C3HC4) 1 | 4 | 0.764 | 6.50E-10 | ✔ |
| ILMN_1696975 | USP1 | ubiquitin specific peptidase 1 | 1 | 0.763 | 7.35E-10 | ✔ |
| ILMN_1755926 | DBI | diazepam binding inhibitor (GABA receptor modulator, acyl-Coenzyme A binding protein) | 2 | 0.760 | 8.70E-10 | ✔ |
| **ILMN_1719749** | **PTGES3*** | **prostaglandin E synthase 3 (cytosolic)** | **12** | **0.760** | **8.92E-10** |  |
| ILMN_1661346 | LOC648210 | PREDICTED: similar to Heterogeneous nuclear ribonucleoprotein A1 (Helix-destabilizing protein) (Single-strand RNA-binding protein) (hnRNP core protein A1) |  | 0.760 | 9.40E-10 |  |
| ILMN_2299072 | CROP | cisplatin resistance-associated overexpressed protein | 17 | 0.759 | 9.57E-10 |  |
| ILMN_2096191 | AASDHPPT | aminoadipate-semialdehyde dehydrogenase-phosphopantetheinyl transferase | 11 | 0.759 | 9.67E-10 | ✔ |
| ILMN_2142117 | LYPLAL1 | lysophospholipase-like 1 | 1 | 0.755 | 1.37E-09 | ✔ |
| ILMN_2067607 | TMEM106B | transmembrane protein 106B | 7 | 0.753 | 1.61E-09 | ✔ |
| ILMN_1780058 | DEGS1 | degenerative spermatocyte homolog 1, lipid desaturase | 1 | 0.743 | 3.30E-09 | ✔ |
| ILMN_1676159 | MST4 | serine/threonine protein kinase MST4 | X | 0.741 | 3.94E-09 | ✔ |
| ILMN_1807234 | ZNF700 | zinc finger protein 700 | 19 | 0.741 | 3.95E-09 | ✔ |
| ILMN_2381397 | HSPD1 | heat shock 60kDa protein 1 (chaperonin) | 2 | 0.741 | 3.99E-09 | ✔ |
| ILMN_2086095 | ID2 | inhibitor of DNA binding 2, dominant negative helix-loop-helix protein | 2 | 0.740 | 4.18E-09 | ✔ |
| ILMN_1758811 | IMPA1 | inositol(myo)-1(or 4)-monophosphatase 1 | 8 | 0.739 | 4.39E-09 | ✔ |
| ILMN_2407082 | SEP15 | 15 kDa selenoprotein | 1 | 0.738 | 4.96E-09 | ✔ |
| ILMN_1809439 | HMGB1L1 | high-mobility group box 1-like 1 | 20 | 0.737 | 5.10E-09 |  |
| ILMN_1778611 | GBAS | glioblastoma amplified sequence | 7 | 0.734 | 6.63E-09 | ✔ |
| ILMN_2284744 | HNMT | histamine N-methyltransferase | 2 | 0.733 | 6.87E-09 | ✔ |
| ILMN_1680223 | PNPLA8 | patatin-like phospholipase domain containing 8 | 7 | 0.733 | 6.95E-09 | ✔ |
| ILMN_1742813 | TMEM167A | transmembrane protein 167A | 5 | 0.733 | 7.17E-09 | ✔ |
| ILMN_2257665 | PARL | presenilin associated, rhomboid-like | 3 | 0.733 | 7.19E-09 | ✔ |
| ILMN_1798254 | ACTR10 | actin-related protein 10 homolog | 14 | 0.732 | 7.26E-09 | ✔ |
| ILMN_1682233 | ESCO1 | establishment of cohesion 1 homolog 1 | 18 | 0.730 | 8.85E-09 | ✔ |
| ILMN_1690999 | MED23 | mediator complex subunit 23 | 6 | 0.727 | 1.04E-08 | ✔ |
| ILMN_2181883 | C14orf129 | chromosome 14 open reading frame 129 | 14 | 0.725 | 1.22E-08 |  |
| **ILMN_1759954** | **PTMA*** | **prothymosin, alpha** | **2** | **0.724** | **1.33E-08** | **✔** |
| **ILMN_1756860** | **TXNL1*** | **thioredoxin-like 1** | **18** | **0.724** | **1.34E-08** | **✔** |
| ILMN_2147517 | CD58 | CD58 molecule | 1 | 0.721 | 1.55E-08 | ✔ |
| ILMN_1686811 | LOC402644 | PREDICTED: similar to peptidylprolyl isomerase A isoform 1 |  | 0.720 | 1.71E-08 |  |
| ILMN_1701855 | PPP1CC | protein phosphatase 1, catalytic subunit, gamma isoform | 12 | 0.719 | 1.77E-08 | ✔ |
| ILMN_2395974 | PRDX3 | peroxiredoxin 3 | 10 | 0.711 | 3.16E-08 | ✔ |
| ILMN_1799487 | N4BP2L1 | NEDD4 binding protein 2-like 1 | 13 | 0.711 | 3.18E-08 |  |
| ILMN_1694491 | CCNG1 | cyclin G1 | 5 | 0.705 | 4.66E-08 | ✔ |
| ILMN_2411264 | BTBD1 | BTB (POZ) domain containing 1 | 15 | 0.697 | 7.31E-08 | ✔ |
| ILMN_1731194 | STRAP | serine/threonine kinase receptor associated protein | 12 | 0.688 | 1.28E-07 | ✔ |
| ILMN_1681101 | MAR1 | membrane-associated ring finger (C3HC4) 1 | 4 | 0.687 | 1.39E-07 |  |
| ILMN_1770127 | DNAJA2 | DnaJ (Hsp40) homolog, subfamily A, member 2 | 16 | 0.682 | 1.79E-07 | ✔ |
| ILMN_1714759 | CNIH4 | cornichon homolog 4 | 1 | 0.680 | 1.99E-07 | ✔ |
| ILMN_1729509 | C1orf43 | chromosome 1 open reading frame 43 | 1 | 0.677 | 2.45E-07 |  |
| ILMN_1734229 | SPPL2A | signal peptide peptidase-like 2A | 15 | 0.676 | 2.50E-07 | ✔ |
| ILMN_1691402 | LOC644162 | PREDICTED: similar to septin 7 | 10 | 0.669 | 3.81E-07 |  |
| ILMN_1711617 | GMFG | glia maturation factor | 19 | 0.668 | 3.84E-07 | ✔ |
| ILMN_1769895 | CCR2 | chemokine (C-C motif) receptor 2 | 3 | 0.668 | 4.01E-07 |  |
| ILMN_1679280 | LOC643997 | PREDICTED: similar to peptidylprolyl isomerase A isoform 1 | 2 | 0.663 | 5.07E-07 |  |
| ILMN_1811367 | MAT2B | methionine adenosyltransferase II, beta | 5 | 0.662 | 5.32E-07 | ✔ |
| ILMN_1703005 | IFP38 | IFP38 |  | 0.658 | 6.57E-07 |  |
| ILMN_1677440 | ATP6AP2 | ATPase, H+ transporting, lysosomal accessory protein 2 | X | 0.656 | 7.31E-07 | ✔ |
| ILMN_1702946 | THUMPD1 | THUMP domain containing 1 | 16 | 0.652 | 9.23E-07 | ✔ |
| ILMN_2386100 | BUB3 | BUB3 budding uninhibited by benzimidazoles 3 homolog | 10 | 0.651 | 9.80E-07 | ✔ |
| ILMN_1772369 | PDHA1 | pyruvate dehydrogenase (lipoamide) alpha 1 | X | 0.646 | 1.23E-06 | ✔ |
| ILMN_1721868 | KPNA2 | karyopherin alpha 2 (RAG cohort 1, importin alpha 1) | 17 | 0.645 | 1.28E-06 |  |
| ILMN_2091347 | IDH1 | isocitrate dehydrogenase 1 (NADP+), soluble | 2 | 0.645 | 1.29E-06 | ✔ |
| **ILMN_1783304** | **ATP1B3*** | **PREDICTED: ATPase, Na+/K+ transporting, beta 3 polypeptide** |  | **0.644** | **1.35E-06** |  |
| ILMN_1740749 | RPL7A | ribosomal protein L7a | 9 | 0.638 | 1.84E-06 |  |
| ILMN_1655827 | COPS2 | COP9 constitutive photomorphogenic homolog subunit 2 | 15 | 0.632 | 2.42E-06 | ✔ |
| ILMN_1733176 | LIMS1 | PREDICTED: LIM and senescent cell antigen-like domains 1 |  | 0.632 | 2.46E-06 | ✔ |
| ILMN_1703229 | HIATL1 | hippocampus abundant transcript-like 1 | 9 | 0.629 | 2.84E-06 | ✔ |
| ILMN_1666178 | TP53I13 | tumor protein p53 inducible protein 13 | 17 | 0.626 | 3.23E-06 | ✔ |
| ILMN_1813240 | EIF1AX | eukaryotic translation initiation factor 1A, X-linked | X | 0.624 | 3.64E-06 | ✔ |
| ILMN_2323992 | CLEC7A | C-type lectin domain family 7, member A | 12 | 0.621 | 4.21E-06 |  |
| ILMN_1770053 | RBBP7 | retinoblastoma binding protein 7 | X | 0.607 | 7.71E-06 | ✔ |
| ILMN_1759628 | ATP1B3 | PREDICTED: ATPase, Na+/K+ transporting, beta 3 polypeptide |  | 0.607 | 7.85E-06 |  |
| ILMN_1769931 | SFPQ | splicing factor proline/glutamine-rich (polypyrimidine tract binding protein associated) | 1 | 0.604 | 8.63E-06 | ✔ |
| ILMN_1791792 | C12orf5 | chromosome 12 open reading frame 5 | 12 | 0.597 | 1.19E-05 |  |
| ILMN_1747759 | WSB1 | WD repeat and SOCS box-containing 1 | 17 | 0.590 | 1.61E-05 | ✔ |
| ILMN_1677906 | LOC643287 | PREDICTED: similar to prothymosin alpha | 12 | 0.586 | 1.86E-05 |  |
| ILMN_2096372 | ALDH1A1 | aldehyde dehydrogenase 1 family, member A1 | 9 | 0.585 | 1.98E-05 | ✔ |
| ILMN_1682312 | CYBB | cytochrome b-245, beta polypeptide (chronic granulomatous disease) | X | 0.583 | 2.14E-05 | ✔ |
| ILMN_1705876 | NAP1L1 | nucleosome assembly protein 1-like 1 | 12 | 0.574 | 3.02E-05 | ✔ |
| ILMN_1657993 | ADNP | activity-dependent neuroprotector homeobox | 20 | 0.571 | 3.36E-05 | ✔ |
| ILMN_2311761 | AP3S1 | adaptor-related protein complex 3, sigma 1 subunit | 5 | 0.569 | 3.66E-05 | ✔ |
| ILMN_1776939 | MS4A1 | membrane-spanning 4-domains, subfamily A, member 1 | 11 | 0.561 | 4.91E-05 | ✔ |
| ILMN_1778957 | PNRC2 | proline-rich nuclear receptor coactivator 2 | 1 | 0.556 | 6.01E-05 |  |
| ILMN_2371590 | DDX17 | DEAD (Asp-Glu-Ala-Asp) box polypeptide 17 | 22 | 0.556 | 6.04E-05 | ✔ |
| ILMN_1746171 | H2AFY | H2A histone family, member Y | 5 | 0.552 | 6.94E-05 | ✔ |
| ILMN_2224103 | PAPSS1 | 3'-phosphoadenosine 5'-phosphosulfate synthase 1 | 4 | 0.549 | 7.72E-05 | ✔ |
| ILMN_1677827 | TLR7 | toll-like receptor 7 | X | 0.547 | 8.37E-05 | ✔ |
| ILMN_1683792 | LAP3 | leucine aminopeptidase 3 | 4 | 0.547 | 8.40E-05 | ✔ |
| ILMN_2380946 | EIF4G2 | eukaryotic translation initiation factor 4 gamma, 2 | 11 | 0.544 | 9.21E-05 | ✔ |
| ILMN_2367070 | ACOT9 | acyl-CoA thioesterase 9 | X | 0.542 | 9.98E-05 | **✔** |
| ILMN_1769245 | GLIPR1 | GLI pathogenesis-related 1 | 12 | 0.541 | 1.05E-04 | ✔ |
| ILMN_1769546 | RIN2 | Ras and Rab interactor 2 | 20 | 0.538 | 1.16E-04 | ✔ |
| ILMN_2285568 | NAAA | N-acylethanolamine acid amidase | 4 | 0.533 | 1.37E-04 | ✔ |
| ILMN_1713163 | SMARCA5 | SWI/SNF related, matrix associated, actin dependent regulator of chromatin, subfamily a, member 5 | 4 | 0.527 | 1.67E-04 | ✔ |
| ILMN_2368713 | TMEM189-UBE2V1 | TMEM189-UBE2V1 readthrough transcript | 20 | 0.523 | 1.91E-04 |  |
| ILMN_2390310 | C17orf91 | chromosome 17 open reading frame 91 | 17 | 0.523 | 1.92E-04 |  |
| ILMN_1805992 | KIAA1598 | KIAA1598 | 10 | 0.522 | 2.01E-04 | ✔ |
| ILMN_1682928 | CPVL | carboxypeptidase, vitellogenic-like | 7 | 0.520 | 2.13E-04 | ✔ |
| ILMN_1719611 | CCT6A | chaperonin containing TCP1, subunit 6A (zeta 1) | 7 | 0.517 | 2.34E-04 | ✔ |
| ILMN_2255133 | BCL11A | B-cell CLL/lymphoma 11A (zinc finger protein) | 2 | 0.517 | 2.34E-04 | ✔ |
| ILMN_2186806 | HLA-F | major histocompatibility complex, class I, F | 6 | 0.513 | 2.65E-04 | ✔ |
| ILMN_1708604 | C7orf28A | chromosome 7 open reading frame 28A | 7 | 0.512 | 2.80E-04 |  |
| ILMN_1693009 | FGL2 | fibrinogen-like 2 | 7 | 0.508 | 3.17E-04 | ✔ |
| ILMN_1703263 | SP140 | SP140 nuclear body protein | 2 | 0.499 | 4.10E-04 |  |
| ILMN_1777461 | CCR2 | chemokine (C-C motif) receptor 2 |  | 0.495 | 4.67E-04 |  |
| ILMN_1751034 | ITPRIPL2 | inositol 1,4,5-triphosphate receptor interacting protein-like 2 | 16 | 0.494 | 4.84E-04 |  |
| ILMN_2103362 | ARHGAP27 | Rho GTPase activating protein 27 | 17 | 0.492 | 5.08E-04 | ✔ |
| ILMN_1730685 | MRPL16 | mitochondrial ribosomal protein L1 | 11 | 0.491 | 5.35E-04 | ✔ |
| ILMN_1811117 | LOC400986 | PREDICTED: protein immuno-reactive with anti-PTH polyclonal antibodies |  | 0.487 | 6.01E-04 |  |
| ILMN_1687824 | SEPHS2 | selenophosphate synthetase 2 | 16 | 0.487 | 6.03E-04 | ✔ |
| ILMN_1769734 | NT5C3 | 5'-nucleotidase, cytosolic III | 7 | 0.485 | 6.31E-04 | ✔ |
| ILMN_2041161 | DENND4A | DENN/MADD domain containing 4A | 15 | 0.479 | 7.56E-04 | ✔ |
| ILMN_1688780 | S100A4 | S100 calcium binding protein A4 | 1 | 0.476 | 8.20E-04 |  |
| ILMN_1780189 | PSMC5 | proteasome (prosome, macropain) 26S subunit, ATPase, 5 | 17 | 0.467 | 1.07E-03 | ✔ |
| ILMN_1784774 | P2RY10 | purinergic receptor P2Y, G-protein coupled, 10 | X | 0.466 | 1.10E-03 | ✔ |
| ILMN_1690342 | LTA4H | leukotriene A4 hydrolase | 12 | 0.463 | 1.21E-03 | ✔ |
| ILMN_1719433 | CD1D | CD1d molecule | 1 | 0.457 | 1.41E-03 | ✔ |
| ILMN_1662846 | GPR160 | G protein-coupled receptor 160 | 3 | 0.456 | 1.46E-03 | ✔ |
| ILMN_2259119 | PRMT2 | protein arginine methyltransferase 2 | 21 | 0.455 | 1.47E-03 | ✔ |
| ILMN_1783247 | C10orf11 | chromosome 10 open reading frame 11 | 10 | 0.445 | 1.94E-03 |  |
| ILMN_1658911 | LOC647349 | PREDICTED: similar to AP-3 complex subunit sigma-1 |  | 0.443 | 2.04E-03 |  |
| ILMN_2401714 | MS4A1 | membrane-spanning 4-domains, subfamily A, member 1 | 11 | 0.433 | 2.64E-03 | ✔ |
| ILMN_2219556 | ISCA1 | iron-sulfur cluster assembly 1 homolog | 9 | 0.432 | 2.70E-03 | ✔ |
| ILMN_1765222 | CARS2 | cysteinyl-tRNA synthetase 2, mitochondrial (putative), | 13 | 0.432 | 2.72E-03 | ✔ |
| ILMN_1701947 | GPR34 | G protein-coupled receptor 34 | X | 0.429 | 2.93E-03 | ✔ |
| ILMN_1719392 | FH | fumarate hydratase | 1 | 0.427 | 3.10E-03 | ✔ |
| ILMN_1660661 | TCP1 | t-complex 1 | 6 | 0.424 | 3.30E-03 | ✔ |
| ILMN_2400759 | CPVL | carboxypeptidase, vitellogenic-like | 7 | 0.423 | 3.43E-03 | ✔ |
| ILMN_1669062 | CCR2 | chemokine (C-C motif) receptor 2 |  | 0.416 | 4.05E-03 |  |
| ILMN_1760779 | ENSA | endosulfine alpha | 1 | 0.412 | 4.41E-03 | ✔ |
| ILMN_1665235 | CRTAP | cartilage associated protein | 3 | 0.404 | 5.36E-03 | ✔ |
| ILMN_1651828 | CCT3 | chaperonin containing TCP1, subunit 3 | 1 | 0.403 | 5.53E-03 | ✔ |
| ILMN_1708110 | TMEM144 | transmembrane protein 144 | 4 | 0.402 | 5.60E-03 | ✔ |
| ILMN_2058141 | HMGN2 | high-mobility group nucleosomal binding domain 2 | 1 | 0.394 | 6.77E-03 | ✔ |
| ILMN_1703335 | LACTB | lactamase, | 15 | 0.393 | 6.93E-03 | ✔ |
| ILMN_2086222 | BRWD2 | bromodomain and WD repeat domain containing 2 | 10 | 0.391 | 7.24E-03 |  |
| ILMN_2352121 | NT5C3 | 5'-nucleotidase, cytosolic III | 7 | 0.389 | 7.55E-03 | ✔ |
| ILMN_2331087 | MS4A7 | membrane-spanning 4-domains, subfamily A, member 7 | 11 | 0.372 | 1.09E-02 |  |
| ILMN_1717261 | HLA-DRB3 | major histocompatibility complex, class II, DR beta 3 | 6 | 0.350 | 1.70E-02 |  |
| ILMN_1744914 | FUCA2 | fucosidase, alpha-L- 2, plasma | 6 | 0.344 | 1.91E-02 | ✔ |
| ILMN_1691574 | LOC646064 | PREDICTED: hypothetical protein LOC646064 | 16 | 0.344 | 1.92E-02 |  |
| ILMN_1691693 | FCRL3 | Fc receptor-like 3 | 1 | 0.338 | 2.14E-02 |  |
| ILMN_1766200 | CALHM2 | calcium homeostasis modulator 2 | 10 | 0.337 | 2.22E-02 | ✔ |
| ILMN_2137789 | KLF4 | Kruppel-like factor 4 | 9 | 0.336 | 2.24E-02 | ✔ |
| ILMN_1793461 | LOC653604 | PREDICTED: similar to H3 histone, family 2 isoform 2 | 1 | 0.335 | 2.31E-02 |  |
| ILMN_1731001 | ERICH1 | glutamate-rich 1 | 8 | 0.334 | 2.32E-02 | ✔ |
| ILMN_1691104 | PGAM4 | phosphoglycerate mutase family member 4 | X | 0.332 | 2.43E-02 |  |
| ILMN_2051519 | RPL37A | ribosomal protein L37a | 2 | 0.316 | 3.27E-02 | ✔ |
| ILMN_1790555 | CCDC146 | coiled-coil domain containing 146 | 7 | 0.302 | 4.13E-02 | ✔ |
| ILMN_2154053 | FVT1 | follicular lymphoma variant translocation 1 | 18 | 0.278 | 6.16E-02 |  |
| ILMN_2222688 | TMSB4X | thymosin, beta 4, X-linked | X | 0.269 | 7.09E-02 |  |
| ILMN_2355953 | LILRB4 | leukocyte immunoglobulin-like receptor, subfamily B (with TM and ITIM domains), member 4 | 19 | 0.266 | 7.45E-02 | ✔ |
| ILMN_2364062 | THOC4 | THO complex 4 | 17 | 0.264 | 7.65E-02 | ✔ |
| ILMN_2173294 | FLJ10916 | hypothetical protein FLJ10916 | 2 | 0.252 | 9.14E-02 |  |
| ILMN_1708248 | LILRB1 | leukocyte immunoglobulin-like receptor, subfamily B (with TM and ITIM domains), member 1 | 19 | 0.246 | 9.97E-02 |  |
| ILMN_2246083 | C7orf28B | chromosome 7 open reading frame 28B | 7 | 0.245 | 1.01E-01 |  |
| ILMN_1788237 | LOC652755 | PREDICTED: similar to Baculoviral IAP repeat-containing protein 1 (Neuronal apoptosis inhibitory protein) |  | 0.242 | 1.05E-01 |  |
| ILMN_1781236 | LOC554223 | PREDICTED: hypothetical LOC55422 |  | 0.161 | 2.85E-01 |  |
| ILMN_2258982 | XAGE1 | X antigen family, member 1 | X | 0.127 | 4.01E-01 |  |
