# Supplementary material for: Transcriptome Profiling of Peripheral Blood in 22q11.2 Deletion Syndrome Reveals Functional Pathways Related to Psychosis and Autism Spectrum Disorder
Source: PLoS One. 2015 Jul 22;10(7):e0132542. doi: 10.1371/journal.pone.0132542 (PMC4511766; doi:10.1371/journal.pone.0132542)
Supplement: S6 Table — (DOCX) [file pone.0132542.s017.docx]

**S6 Table. List of genes in the Royal Blue module, which was significantly associated with an ASD diagnosis in 22q11DS.** Column A: Illumina probe name; B: Gene Symbol; C: Gene definition; D: Chromosome location; E: Eigengene correlation value (range 0-1); F: Eigengene *p*-value; G) A “**✔** ” is placed next to genes that are considered to be brain expressed(4). Probes that are highlighted in bold and have an asterisk after the Gene Symbol are probes that significantly overlapped between the Royal Blue module and DE genes in 22q11DS-ASD+.

| **Probe** | **Symbol** | **Gene Name** | **Chromo-some** | **Eigen-gene Corre-lation** | **Eigengene p-value** | **Brain Expressed** |
| --- | --- | --- | --- | --- | --- | --- |
| ILMN_1734878 | CD79A | CD79a molecule, immunoglobulin-associated alpha | 19 | 0.954 | 1.20E-24 |  |
| ILMN_1782704 | CD19 | CD19 molecule | 16 | 0.937 | 1.20E-21 |  |
| **ILMN_1785439** | **CD79B*** | **CD79B antigen (immunoglobulin-associated beta)** | **17** | **0.929** | **1.33E-20** | **✔** |
| **ILMN_2366212** | **CD79B*** | **CD79b molecule, immunoglobulin-associated beta** | **17** | **0.923** | **6.51E-20** | **✔** |
| **ILMN_1710017** | **CD79B*** | **CD79B antigen (immunoglobulin-associated beta)** | **17** | **0.923** | **6.58E-20** | **✔** |
| ILMN_1659227 | CD79A | CD79a molecule, immunoglobulin-associated alpha | 19 | 0.901 | 1.44E-17 |  |
| **ILMN_2337928** | **CXCR5*** | **chemokine (C-X-C motif) receptor 5** | **11** | **0.890** | **1.20E-16** |  |
| **ILMN_1794927** | **LOC90925*** | **hypothetical protein LOC90925** |  | **0.886** | **2.65E-16** |  |
| ILMN_1700147 | VPREB3 | pre-B lymphocyte gene 3 | 22 | 0.887 | 2.25E-16 |  |
| **ILMN_1723004** | **CD72*** | **CD72 molecule** | **9** | **0.865** | **8.79E-15** |  |
| ILMN_1668277 | BLK | B lymphoid tyrosine kinase | 8 | 0.869 | 5.06E-15 |  |
| ILMN_1778681 | EBF1 | early B-cell factor 1 | 5 | 0.862 | 1.40E-14 | ✔ |
| ILMN_1752899 | BCL11A | B-cell CLL/lymphoma 11A (zinc finger protein) | 2 | 0.862 | 1.47E-14 |  |
| **ILMN_1691071** | **FCRLA*** | **Fc receptor-like A** | **1** | **0.847** | **1.24E-13** | **✔** |
| ILMN_1659075 | HLA-DOA | major histocompatibility complex, class II, DO alpha | 6 | 0.838 | 3.59E-13 | ✔ |
| ILMN_1669497 | OSBPL10 | oxysterol binding protein-like 10 | 3 | 0.845 | 1.60E-13 | ✔ |
| ILMN_1792075 | CD22 | CD22 molecule | 19 | 0.838 | 3.78E-13 | ✔ |
| **ILMN_1700428** | **HLA-DOB*** | **major histocompatibility complex, class II, DO beta** | **6** | **0.843** | **2.02E-13** |  |
| **ILMN_1676003** | **PNOC*** | **prepronociceptin** | **8** | **0.840** | **2.90E-13** | **✔** |
| ILMN_1734276 | PMEPA1 | prostate transmembrane protein, androgen induced 1 | 20 | 0.836 | 4.74E-13 | **✔** |
| ILMN_1785175 | SWAP70 | SWAP switching B-cell complex 70kDa subunit | 11 | 0.816 | 4.92E-12 | ✔ |
| ILMN_1719998 | C9orf45 | PREDICTED: chromosome 9 open reading frame 45 | 9 | 0.804 | 1.79E-11 |  |
| **ILMN_1662451** | **FCER2*** | **Fc fragment of IgE, low affinity II, receptor for (CD23)** | **19** | **0.809** | **1.03E-11** |  |
| ILMN_1782551 | E2F5 | E2F transcription factor 5, p130-binding | 8 | 0.775 | 2.53E-10 | ✔ |
| ILMN_1731742 | TNFRSF13C | tumor necrosis factor receptor superfamily, member 13C | 22 | 0.799 | 2.84E-11 | ✔ |
| ILMN_1898723 |  | cDNA clone IMAGE:3079901 | X | 0.793 | 5.16E-11 |  |
| ILMN_2296950 | APOBEC3F | apolipoprotein B mRNA editing enzyme, catalytic polypeptide-like 3F | 22 | 0.789 | 7.39E-11 | ✔ |
| ILMN_1662038 | LARGE | like-glycosyltransferase | 22 | 0.796 | 3.83E-11 | ✔ |
| ILMN_1664063 | FAM129C | family with sequence similarity 129, member C | 19 | 0.790 | 6.89E-11 |  |
| ILMN_1811049 | POU2AF1 | POU class 2 associating factor 1 (POU2AF1) | 11 | 0.781 | 1.52E-10 |  |
| ILMN_1707491 | KIAA0125 | KIAA0125 | 14 | 0.782 | 1.38E-10 | ✔ |
| **ILMN_1773567** | **LAMA5*** | **laminin, alpha 5** | **20** | **0.771** | **3.60E-10** | **✔** |
| ILMN_1788841 | TCL1A | T-cell leukemia/lymphoma 1A | 14 | 0.765 | 6.04E-10 | ✔ |
| ILMN_2129505 | CYBASC3 | cytochrome b, ascorbate dependent 3 | 11 | 0.738 | 4.69E-09 | ✔ |
| ILMN_1728107 | GNG7 | guanine nucleotide binding protein (G protein), gamma 7 | 19 | 0.769 | 4.33E-10 | ✔ |
| ILMN_1797822 | SEL1L3 | sel-1 suppressor of lin-12-like 3 | 4 | 0.758 | 1.06E-09 | ✔ |
| ILMN_1727045 | RASGRP3 | RAS guanyl releasing protein 3 (calcium and DAG-regulated) | 2 | 0.755 | 1.35E-09 | ✔ |
| ILMN_1730917 | KMO | kynurenine 3-monooxygenase (kynurenine 3-hydroxylase) | 1 | 0.756 | 1.28E-09 | ✔ |
| ILMN_1686152 | GGA2 | golgi associated, gamma adaptin ear containing, ARF binding protein 2 | 16 | 0.727 | 1.04E-08 | ✔ |
| ILMN_1775235 | AFF3 | AF4/FMR2 family, member 3 | 2 | 0.748 | 2.26E-09 | **✔** |
| ILMN_1684724 | CR2 | complement component (3d/Epstein Barr virus) receptor 2 | 1 | 0.751 | 1.89E-09 |  |
| **ILMN_2414762** | **TLR10*** | **toll-like receptor 10** | **4** | **0.743** | **3.31E-09** |  |
| ILMN_1661646 | BANK1 | B-cell scaffold protein with ankyrin repeats 1 | 4 | 0.724 | 1.28E-08 | ✔ |
| ILMN_1693242 | ZNF296 | zinc finger protein 296 | 19 | 0.722 | 1.52E-08 | ✔ |
| ILMN_1761260 | COBLL1 | COBL-like 1 | 2 | 0.704 | 4.83E-08 | ✔ |
| ILMN_1690907 | CCR6 | chemokine (C-C motif) receptor 6 | 6 | 0.729 | 9.40E-09 |  |
| ILMN_1814194 | TCF4 | transcription factor 4 | 18 | 0.721 | 1.56E-08 | ✔ |
| ILMN_1669881 | TSPAN13 | tetraspanin 13 | 7 | 0.686 | 1.46E-07 | ✔ |
| ILMN_1675406 | PPAPDC1B | phosphatidic acid phosphatase type 2 domain containing 1B | 8 | 0.700 | 6.02E-08 | ✔ |
| ILMN_1761968 | PPP1R14A | protein phosphatase 1, regulatory (inhibitor) subunit 14A | 19 | 0.692 | 1.00E-07 | ✔ |
| ILMN_2099528 | BTLA | B and T lymphocyte associated | 3 | 0.657 | 7.26E-07 |  |
| ILMN_1719905 | TLR10 | toll-like receptor 10 | 4 | 0.695 | 8.60E-08 |  |
| ILMN_1812721 | LOC728014 | PREDICTED: similar to huntingtin interacting protein 1 related | 12 | 0.706 | 4.26E-08 |  |
| ILMN_1684445 | FCRL5 | Fc receptor-like 5 | 1 | 0.695 | 8.39E-08 |  |
| ILMN_1661264 | SHMT2 | serine hydroxymethyltransferase 2, nuclear gene encoding mitochondrial protein | 12 | 0.687 | 1.32E-07 | ✔ |
| ILMN_2363058 | PAOX | polyamine oxidase (exo-N4-amino) | 10 | 0.679 | 2.11E-07 | ✔ |
| ILMN_1779257 | CD40 | CD40 molecule, TNF receptor superfamily member 5 | 20 | 0.694 | 8.71E-08 | ✔ |
| ILMN_1745256 | CXXC5 | CXXC finger 5 | 5 | 0.692 | 9.74E-08 | ✔ |
| ILMN_1662878 | PACAP | proapoptotic caspase adaptor protein | 5 | 0.687 | 1.32E-07 |  |
| ILMN_2342271 | BCL11A | B-cell CLL/lymphoma 11A (zinc finger protein) | 2 | 0.661 | 5.66E-07 | ✔ |
| ILMN_1791593 | DENND5B | DENN/MADD domain containing 5B | 12 | 0.669 | 3.82E-07 | ✔ |
| ILMN_1711699 | LOC728014 | PREDICTED: similar to huntingtin interacting protein 1 related | 12 | 0.665 | 4.60E-07 |  |
| ILMN_1810852 | LAMC1 | laminin, gamma 1 | 1 | 0.677 | 2.35E-07 | ✔ |
| ILMN_2369666 | CR2 | complement component (3d/Epstein Barr virus) receptor 2 | 1 | 0.675 | 2.61E-07 |  |
| ILMN_1772876 | ZNF395 | zinc finger protein 395 | 8 | 0.656 | 7.47E-07 | ✔ |
| ILMN_2337931 | CXCR5 | chemokine (C-X-C motif) receptor 5 | 11 | 0.629 | 2.78E-06 |  |
| ILMN_1690064 | RUNDC2C | RUN domain containing 2C | 16 | 0.634 | 2.28E-06 |  |
| ILMN_1699644 | MAR3 | PREDICTED: membrane-associated ring finger (C3HC4) 3 |  | 0.626 | 3.23E-06 | ✔ |
| ILMN_1655796 | MAR3 | PREDICTED: membrane-associated ring finger (C3HC4) 3 |  | 0.621 | 4.05E-06 | ✔ |
| ILMN_1773363 | CIITA | class II, major histocompatibility complex, transactivator | 16 | 0.610 | 6.86E-06 | ✔ |
| ILMN_1759075 | TNFRSF13B | tumor necrosis factor receptor superfamily, member 13B | 17 | 0.567 | 4.06E-05 |  |
| ILMN_1665152 | FCRL2 | Fc receptor-like 2 | 1 | 0.615 | 5.47E-06 |  |
| ILMN_2230683 | CDCA7L | cell division cycle associated 7-like | 7 | 0.615 | 5.43E-06 | ✔ |
| ILMN_2117323 | PIK3C2B | phosphoinositide-3-kinase, class 2, beta polypeptide | 1 | 0.568 | 3.90E-05 | ✔ |
| ILMN_1685124 | TCTN1 | tectonic family member 1 | 12 | 0.561 | 5.09E-05 | **✔** |
| ILMN_1651610 | LOC730525 | PREDICTED: hypothetical protein LOC730525 |  | 0.589 | 1.65E-05 |  |
| ILMN_1741356 | PRICKLE1 | prickle homolog 1 | 12 | 0.557 | 5.90E-05 | ✔ |
| **ILMN_2374352** | **DBNDD1*** | **dysbindin (dystrobrevin binding protein 1) domain containing 1** | **16** | **0.578** | **2.63E-05** | **✔** |
| **ILMN_1742544** | **MEF2C*** | **myocyte enhancer factor 2C** | **5** | **0.566** | **4.14E-05** | **✔** |
| ILMN_1690223 | CNTNAP2 | contactin associated protein-like 2 | 7 | 0.562 | 4.79E-05 | ✔ |
| ILMN_1714364 | PTK2 | PTK2 protein tyrosine kinase 2 | 8 | 0.559 | 5.32E-05 | ✔ |
| ILMN_1736567 | CD74 | CD74 molecule, major histocompatibility complex, class II invariant chain | 5 | 0.539 | 1.12E-04 | **✔** |
| ILMN_1687213 | C8orf13 | chromosome 8 open reading frame 13 | 8 | 0.525 | 1.78E-04 |  |
| ILMN_1673769 | KCNG1 | potassium voltage-gated channel, subfamily G, member 1 | 20 | 0.502 | 3.80E-04 | ✔ |
| ILMN_2382309 | TCL1B | T-cell leukemia/lymphoma 1B | 14 | 0.421 | 3.61E-03 |  |
| ILMN_1699599 | FCRL3 | Fc receptor-like 3 | 1 | 0.387 | 7.83E-03 |  |
